# Conductive Self-Assembled Monolayers of Paramagnetic {Co^II^${\text{Co}}_{4}^{\text{III}}$} and {${\text{Co}}_{4}^{\text{II}}$${\text{Co}}_{2}^{\text{III}}$} Coordination Clusters on Gold Surfaces

**DOI:** 10.3389/fchem.2019.00681

**Published:** 2019-11-05

**Authors:** Sebastian Schmitz, Xinkai Qiu, Maria Glöß, Jan van Leusen, Natalya V. Izarova, Muhammad Arif Nadeem, Jan Griebel, Ryan C. Chiechi, Paul Kögerler, Kirill Yu. Monakhov

**Affiliations:** ^1^Institut für Anorganische Chemie, RWTH Aachen University, Aachen, Germany; ^2^Leibniz Institute of Surface Engineering (IOM), Leipzig, Germany; ^3^Stratingh Institute for Chemistry, Zernike Institute for Advanced Materials, University of Groningen, Groningen, Netherlands; ^4^Jülich-Aachen Research Alliance (JARA-FIT), Peter Grünberg Institute, Forschungszentrum Jülich GmbH, Jülich, Germany; ^5^Department of Chemistry, Quaid-i-Azam University, Islamabad, Pakistan

**Keywords:** cobalt, magnetochemistry, gold surface, molecular conductivity, eutectic gallium–indium electrode

## Abstract

Two polynuclear cobalt(II,III) complexes, [Co_5_(N_3_)_4_(*N*-*n*-bda)_4_(bza·SMe)_2_] (**1**) and [Co_6_(N_3_)_4_(*N*-*n*-bda)_2_(bza·SMe)_5_(MeOH)_4_]Cl (**2**), where Hbza·SMe = 4-(methylthio)benzoic acid and *N*-*n*-H_2_bda = *N*-*n*-butyldiethanolamine, were synthesized and fully characterized by various techniques. Compound **1** exhibits an unusual, approximately *C*_2_-symmetric {Co^II^${\text{Co}}_{4}^{\text{III}}$} core of two isosceles Co_3_ triangles with perpendicularly oriented planes, sharing a central, high-spin Co^II^ ion residing in a distorted tetrahedral coordination environment. This central Co^II^ ion is connected to four outer, octahedrally coordinated low-spin Co^III^ ions *via* oxo bridges. Compound **2** comprises a semi-circular {${\text{Co}}_{4}^{\text{II}}$${\text{Co}}_{2}^{\text{III}}$} motif of four non-interacting high-spin Co^II^ and two low-spin Co^III^ centers in octahedral coordination environments. Self-assembled monolayers (SAMs) of **1** and **2** were physisorbed on template-stripped gold surfaces contacted by an eutectic gallium-indium (EGaIn) tip. The acquired current density-voltage (*I-V*) data revealed that the cobalt-based SAMs are more electrically robust than those of the previously reported dinuclear {Cu^II^Ln^III^} complexes with Ln = Gd, Tb, Dy, or Y (Schmitz et al., 2018a). In addition, between 170 and 220°C, the neutral, mixed-valence compound **1** undergoes a redox modification, yielding a {Co_5_}-based coordination cluster (**1-A**) with five non-interacting, high-spin octahedral Co^II^ centers as indicated by SQUID magnetometry analysis in combination with X-ray photoelectron spectroscopy and infrared spectroscopy. Solvothermal treatment of **1** results in a high-nuclearity coordination cluster, [Co_10_(N_3_)_2_(*N*-*n*-bda)_6_(bza·SMe)_6_] (**3**), containing 10 virtually non-interacting high-spin Co^II^ centers.

**Graphical Abstract:**
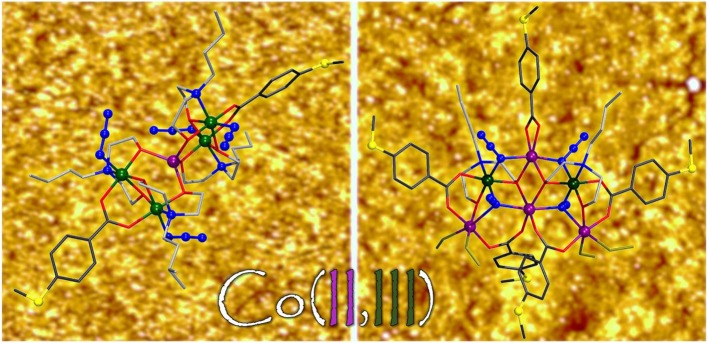
The synthesis, structure, magnetochemistry and adsorption behavior of several new cobalt coordination clusters on gold surfaces are reported.

## Introduction

The implementation of metal coordination complexes with specific paramagnetic and/or redox properties as single molecules or as constituents of two-dimensional molecular monolayers (Yao et al., 2019) into computer memory cells (Linnenberg et al., 2018) is a promising way to keep the miniaturization and sustainability of electronic components from colliding with the so-called “quantum limit” (sub-10-nm regime) valid. At both these levels of molecule–surface interfaces, preservation of the main molecular characteristics (Mitcov et al., 2019) as identified in the bulk state, and avoidance of the agglomerative behavior of coordination compounds after their immobilization on solid support constitute important milestones in the controlled (micro-)spectroscopic addressing of the tunnel junction structures of molecule–electrode hybrid devices. The eventual step to practical devices (Sun et al., 2014) mandates an in-depth understanding of the adsorption, autonomous self-organization, electron charge-/spin-transport characteristics (Al-Owaedi et al., 2017; Bu et al., 2018) and switching mechanisms of mono- and polynuclear complexes of transition metals (Higgins and Nichols, 2018), lanthanides (Dreiser, 2015), and their mixed-metal derivatives (Schmitz et al., 2018a) on conductive and semi-conductive surfaces (Cornia et al., 2011).

Based on our ongoing interest in investigating the large-area charge transport properties of moisture-stable polynuclear transition metal complexes, we describe herein the synthesis, structure, and magnetochemistry of novel mixed-valent Co^II/III^ and mono-valent Co^II^ coordination clusters and assess their abilities to exhibit reproducible electrical conductivity features when deposited on a metallic substrate. The present study is motivated by the appealing electron transport features and the Kondo effect (Yan and Seminario, 2005; Parks et al., 2010) of mononuclear complexes of an octahedrally coordinated cobalt ion attached to gold electrodes *via* thiol bonds. The augmentation of our polynuclear complexes with ancillary groups like thioethers (Dreiser et al., 2015; Schmitz et al., 2018c) aims to contribute to their conformational and charge stabilization on metallic surfaces and, furthermore, to reduce their possible agglomeration due to steric repulsion between the structurally exposed –SR anchor points. It is however noteworthy that metal complexes derivatized by thiane functionalities as, for example, mononuclear copper(II) pyridyl–alcohol complexes are capable of featuring close intermolecular S···S contacts in the crystal lattice (Schmitz et al., 2019).

In the elaborated two-terminal model cell setup the prepared, solution-processable cobalt compounds were adsorbed on an air-stable gold surface as bottom electrode and contacted by a Ga_2_O_3_-terminated EGaIn top electrode (Reus et al., 2012), a method that allows the formation of self-assembled monolayers (Wang et al., 2011) (hereinafter referred to as SAMs) and the examination of electron transport through electrically accessible metal centers. The SAM's morphology, thickness, and current density–voltage characteristics were effectively interpreted using a combination of atomic force microscopy (AFM), scanning tunneling microscopy (STM), and ellipsometry. The findings obtained by these analyses are compared to those reported by us for thioether-Schiff base (L) supported [CuLn(L·SMe)_2_(OOCMe)_2_(NO_3_)] complexes with Ln = Gd, Tb, Dy, or Y (Schmitz et al., 2018a).

## Experimental

### Materials and Methods

The synthesis of compounds **1**–**3** was carried out under aerobic conditions. All starting materials were commercial and used as received. Solvents were used without further purification. Elemental analyses were performed using a Vario EL elemental analyzer to determine the C, H, N, and S (deviation ±0.05) composition in the polycrystalline samples of compounds **1**, **1-A**, and **2** and using an Elementar Vario EL III for the C, H, and N determination of the polycrystalline sample of compound **3**. IR spectra of solid samples were recorded on Nicolet Avatar 360 (**1**, **1-A**, and **2**) and Bruker TENSOR II (**3**) FTIR spectrometers using KBr pellets (*m*_KBr_ ≈ 250 mg) in the range ṽ = 4000–400 cm^−1^. TG/DTA curves of compounds **1**–**3** were obtained under a nitrogen flux (**1** and **2**) and under a flow of dry air (**1** and **3**) with a heating rate of 5 K min^−1^ in the temperature range 25–800°C by using a Mettler Toledo TGA/SDTA 851e instrument. The thermally dependent EI-MS of compound **1** was recorded on a Shimadzu GCMS-QP2010S equipped with a Direct Sample Inlet Device DI-2010. The ESI mass spectrometry patterns of compounds **1** and **2** in the positive ion mode were recorded on a 4000 QTRAP mass spectrometer system by using the LC/LC-MS method with direct infusion.

### Synthesis of [Co^II^${\text{Co}}_{4}^{\text{III}}$(N_3_)_4_(*N*-*n*-bda)_4_(bza·SMe)_2_]·H_2_O (1)

Co(OOCMe)_2_·4H_2_O (0.250 g, 1.0 mmol) (alternatively, 1.0 mmol of CoSO_4_·7H_2_O), NaN_3_ (0.065 g, 1.0 mmol), and 4-(methylthio)benzoic acid (0.168 g, 1.0 mmol) were dissolved in 6 ml of MeOH to afford the violet mixture. 0.170 ml (1.0 mmol) of *N*-*n*-butyldiethanolamine was then added. The reaction mixture was stirred under reflux for 3 h and filtered off, and the MeOH filtrate was kept in a capped vial at room temperature. The color of the solution changed from violet to green, and dark-green block-shaped crystals of compound **1** were formed after 3 weeks. The crystals were isolated and washed with ice-cold MeOH. Yield of air-dried crystals: 0.122 g (42% based on Co). **Elemental analysis**: calcd. for [Co^II^${\text{Co}}_{4}^{\text{III}}$(N_3_)_4_(*N*-*n*-bda)_4_(bza·SMe)_2_]·1.75H_2_O (C_48_H_85.5_Co_5_N_16_O_13.75_S_2_, 1,465.58 g mol^−1^): C, 39.34; H, 5.88; N, 15.29 and S, 4.38%. Found: C, 39.30; H, 5.85; N, 15.50 and S, 4.20%. **IR** (KBr pellet, ṽ_max_/cm^−1^): 3,440 (s, br), 2,955 (m), 2,927 (m, sh), 2,867 (m), 2,020 (s), 1,632 (w, sh), 1,580 (m), 1,539 (m), 1,439 (w), 1,408 (s), 1,384 (w, sh), 1,287 (w), 1,185 (w), 1,089 (w, sh), 1,067 (m), 1,030 (w), 1,013 (w), 979 (w), 917 (m), 863 (w), 845 (w), 767 (m), 684 (w), 653 (w), 602 (w), 574 (w), 519 (m), 483 (w). **MS** (MeOH, ESI, *m/z*): 1,456.231 (measured), 1,456.229 (calcd. for C_48_Co_5_H_82_N_16_NaO_12_${\text{S}}_{2}^{+}$, 100%, [NaCo^II^${\text{Co}}_{4}^{\text{III}}$(N_3_)_4_(*N*-*n*-bda)_4_(bza·SMe)_2_]^+^+Na^+^).

### Preparation of [${\text{Co}}_{5}^{\text{II}}$(N_3_)_2_(*N*-*n*-bda)_2_(bae)_2_(bza·SMe)_2_]·1.5H_2_O (1-A)

The dark-green polycrystalline compound **1** was gradually heated up to 220°C for 15 min in a N_2_ flow, which resulted in a violet microcrystalline powder that was subjected to further characterization. The resulting product was however not suitable for X-ray diffraction analysis. **Elemental analysis**: calcd. for [${\text{Co}}_{5}^{\text{II}}$(N_3_)_2_(*N*-*n*-bda)_2_(bae)_2_(bza·SMe)_2_]·1.5H_2_O (Hbae = 2-(butylamino)ethan-1-ol) (C_44_Co_5_H_76_N_10_O_10_S_2_·1.5H_2_O, 1,290.95 g mol^−1^): C, 40.94; H, 6.17; N, 10.85 and S, 4.97%. Found: C, 41.10; H, 5.63; N, 10.13; and S, 4.84%. **IR** (KBr pellet, ṽ_max_/cm^−1^): 3,424 (m, br), 2,954 (s), 2,926 (m, sh), 2,871 (m, sh), 2,057 (s), 1,593 (s), 1,534 (s), 1,495 (w), 1,422 (s), 1,317 (w), 1,270 (w), 1,182 (m), 1,076 (s), 1,039 (w, sh), 1,016 (w, sh), 985 (w), 892 (m), 858 (m), 769 (m), 692 (w), 619 (w), 580 (w), 533 (w), 508 (w), 471 (w).

### Synthesis of [${\text{Co}}_{4}^{\text{II}}$${\text{Co}}_{2}^{\text{III}}$(N_3_)_4_(*N*-*n*-bda)_2_(bza·SMe)_5_(MeOH)_4_]Cl·2.25MeOH (2)

Compound **2** was prepared following the protocol for the synthesis of compound **1**, but using CoCl_2_·6H_2_O (0.238 g, 1.0 mmol) instead of Co(OOCMe)_2_·4H_2_O. Yield of air-dried crystals: 0.057 g (19% based on Co). **Elemental analysis**: calcd. for [${\text{Co}}_{4}^{\text{II}}$${\text{Co}}_{2}^{\text{III}}$(N_3_)_4_(*N*-*n*-bda)_2_(bza·SMe)_5_]Cl·8H_2_O (C_56_Cl_1_Co_6_H_85_N_14_O_22_S_5_, 1,855.73 g mol^−1^): C, 36.24; H, 4.62; N, 10.57 and S, 8.64%. Found: C, 35.99; H, 4.10; N, 10.65; and S, 8.35%. **IR** (KBr pellet, ṽ_max_/cm^−1^): 3,427 (s, br), 2,959 (m), 2,924 (m), 2,871 (w), 2,087 (vs), 1,593 (s), 1,540 (m), 1,403 (s), 1,286 (m), 1,257 (w, sh), 1,216 (w), 1,184 (m), 1,089 (m), 1,014 (w), 967 (w), 925 (w), 847 (w), 771 (m), 693 (w), 633 (w), 581 (w), 517 (w), 479 (m). **MS** (MeOH, ESI, *m/z*): 1,675.019 (measured), 1,674.974 (calcd. for C_56_Co_6_H_69_N_14_O_14_${\text{S}}_{5}^{+}$, 100%; [Co_6_(N_3_)_4_(*N*-*n*-bda)_2_(bza·SMe)_5_]^+^–4MeOH).

### Synthesis of [${\text{Co}}_{10}^{\text{II}}$(N_3_)_2_(*N*-*n*-bda)_6_(bza·SMe)_6_] (3)

Freshly prepared and dried polycrystalline compound **1** (0.050 g, 0.035 mmol) was dissolved in 5 ml of DMSO and pyrazine was added as a crystallization reagent (0.030 g, 0.375 mmol). The resulting green solution was placed in an autoclave-stable glass vial and reacted in an oven at 100°C for 120 h under autogenous pressure. The reaction solution was cooled down to room temperature over 4 h. Pink single crystals of **3** were collected by filtration, washed with ice-cold EtOH, and dried in air. Yield of air-dried crystals: 0.011 g (24% based on Co). Please note that the synthesis is also successful in the absence of pyrazine, but the yield of crystalline product is much lower. **Elemental analysis**: calcd. for [${\text{Co}}_{10}^{\text{II}}$(N_3_)_2_(*N*-*n*-bda)_6_(bza·SMe)_6_]·0.7pyrazine·6H_2_O (Co_10_C_96_H_144_N_12_O_24_S_6_·0.7pyrazine·6H_2_O, 2,631.95 g mol^−1^, disregarding solvent molecules): C, 42.44; H, 5.72; and N, 6.71%. Found: C, 42.32; H, 5.35 and N, 7.04%. **IR** (KBr pellet, ṽ_max_/cm^−1^): 3,442 (m, br), 2,954 (m), 2,926 (m, sh), 2,871 (m), 2,853 (m, sh), 2,058 (vs), 1,593 (s), 1,534 (s), 1,496 (w), 1,463 (w, sh), 1,422 (vs), 1,384 (w, sh), 1,317 (w), 1,270 (w), 1,182 (w), 1,136 (vw), 1,091 (m), 1,076 (s), 1,040 (m, sh), 1,017 (m, sh), 985 (w), 968 (vw), 909 (w), 888 (w), 857 (m), 769 (m), 713 (vw), 693 (w), 669 (vw), 631 (vw), 579 (w), 534 (w), 508 (vw), 474 (w), 419 (w).

### X-Ray Crystallography

Single-crystal diffraction data for **1**–**3** were collected on a SuperNova (Agilent Technologies) diffractometer with MoKa radiation (*l* = 0.71073 ) at 120 K (for **1** and **2**) and on a Bruker APEX II CCD diffractometer at 100 K (for **3**). The crystals were mounted in a Hampton cryoloop with Paratone-N oil to prevent water loss. Absorption corrections were applied either analytically based on multifaceted crystal model using CrysAlis software (**1**, **2**)^1^ or empirically using the SADABS program (**3**)^2^. The structures were solved by direct methods and refined by full-matrix least-squares method against |F|^2^ with anisotropic thermal parameters for all non-hydrogen atoms (Co, S, O, N, C, and Cl) using the SHELXTL software package (Sheldrick, 2015). Hydrogen atoms were placed in geometrically calculated positions. Hydrogen atoms of the disordered solvent molecules (e.g., H_2_O in **1** and MeOH in **2**), OH groups of the MeOH ligands in **2**, and the disordered –CH_2_-CH_3_ moiety of one of the *N*-*n*-bda^2−^ ligands in **1** and the disordered –Me group of one of the methanol ligands in **2** were not located.

The structure of **1** was refined as a two-component twin (−1 0 0; 0 −1 0; 0 0 −1; BASF 0.52). The relative site occupancy factors for the disordered positions of carbon and oxygen atoms in **1** and in **2** were refined using a combination of PART/EADP or PART/EADP/SUMP instructions.

Additional crystallographic data are summarized in Table S1. Further details on the crystal structure investigation can be obtained, free of charge, on application to CCDC, 12 Union Road, Cambridge CB2 1EZ, UK: http://www.ccdc.cam.ac.uk/, e-mail: data_request@ccdc.cam.ac.uk, or fax: +441223 336033 upon quoting 1936117 (**1**), 1936118 (**2**), and 1936119 (**3**) numbers.

### XPS Measurements

XPS measurements were carried out using a PHI 5000 Versa Probe (Physical Electronics Inc., USA) under ultra-high vacuum conditions (1 × 10^−9^ mbar). The samples were excited with monochromatic Al K_α_ X-rays (1486.6 eV). The energy analyzer operates with a pass energy of 29.35 eV. The take-off angle between the analyzer and the sample was 45°. CasaXPS was used to analyze the spectra. To compensate charging effects, the binding energies were referenced to the C 1s peak at 284.8 eV. The spectra were performed by fitting a convolution of a Lorentzian profile and a Gaussian profile after subtraction of a Shirley background.

### Magnetic Susceptibility Measurements

Magnetic susceptibility data of compounds **1**, **1-A**, **2**, and **3** were recorded using a Quantum Design MPMS-5XL SQUID magnetometer for direct current (dc) and alternating current (ac) measurements. The polycrystalline samples were compacted and immobilized into PTFE capsules. The dc susceptibility data were acquired as a function of the field (0.1–5.0 T) and temperature (2.0–290 K). The ac susceptibility data were measured in the absence of a static bias field in the frequency range 3–1,000 Hz (*T* = 2.0–50.0 K, *B*_ac_ = 3 G), but no out-of-phase signals were observed. The data were corrected for diamagnetic contributions from the sample holders and the compounds [χ_m, dia_/10^−4^ cm^3^ mol^−1^ = −7.17 (**1**); −6.41 (**1-A**); −9.92 (**2**); −13.2 (**3**)]. For the compounds containing octahedrally coordinated, low-spin Co^III^ centers, data were additionally corrected for their TIP contributions (Cossee, 1958) [χ_m, TIP_/10^−4^ cm^3^ mol^−1^ = +1.59 (**1**); +0.80 (**2**)].

### Preparation of the Self-Assembled Monolayers

SAMs of compounds **1** and **2** were prepared by incubating freshly cleaved 1 × 1 cm^2^ Au^TS^ substrates in 5 ml of 0.1 mM methanolic solution of each metal complex overnight. The substrates were then rinsed with methanol (3 × 1 ml) and the residual solvent on the surface was removed under a stream of dry N_2_. The samples were used for measurements and analysis immediately.

### EGaIn Measurements

Electrical measurements with EGaIn were performed under ambient conditions. In the measurements, each sample was grounded and the EGaIn was biased. At least three samples were examined for the SAMs of compounds **1** and **2**. The potential windows include the following: 0 V →1 V→−1 V→ 0 V, steps of 0.05 V. A total of 5 trace/retrace cycles were recorded for each junction, and shorts that occurred during the measurement (short upon contact with a bias of 1 V or during the cycle) were counted for the failure of a junction.

### AFM Measurements

PeakForce Tapping AFM measurements were performed on a Bruker AFM multimode MMAFM-2 model. Pure SAMs of compounds **1** and **2** and freshly cleaved Au^TS^ substrates were characterized by AFM on morphology. The PeakForce Tapping AFM was performed with a ScanAsyst-Air probe (resonant frequency 70 kHz, spring constant 0.4 N/m, Bruker) to characterize the surface morphology of the samples at a scan rate of 0.8 Hz and 768 samples per line. The data were analyzed with Nanoscope Analysis 1.5 provided by Bruker.

### Ellipsometry

Ellipsometry measurements were carried out in air by using a V-Vase Rotating Analyzer equipped with a HS-190 monochromator ellipsometer from J.A. Woollam Co., Inc. A two-layer model consisting of a bottom Au layer, for which optical constants were calculated from freshly prepared template stripped Au surfaces, and a Cauchy layer was used for the fit of the measurements on the SAMs. A chosen value of *n* = 1.45 and *k* = 0 at all wavelengths (i.e., Cauchy parameter An = 1.45, Bn = Cn = 0) was used to fit the thickness.

## Results and Discussion

### Synthesis and Mass-Spectrometric Characterization

**Scheme 1:**
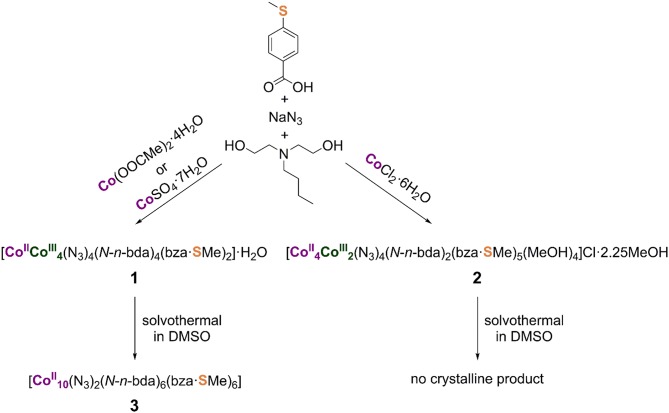
Synthesis of the mixed-valence pentanuclear and hexanuclear cobalt(II,III) complexes and the reduced decanuclear cobalt(II) complex, all stable against air and moisture. The coordination modes of the ligands in the synthesized metal complexes are illustrated in the Supporting Information. Note that the *N*-*n*-H_2_bda ligand is not often present in homonuclear cobalt complexes (see e.g., Scheurer et al., 2010).

Compounds **1** and **2** were synthesized *via* one-pot aerobic reactions summarized in Scheme 1. The choice of cobalt precursor under otherwise identical reaction conditions is key to the isolation of the desired compound. In the general reaction procedure, a cobalt(II) salt and 4-(methylthio)benzoic acid (Hbza·SMe) were dissolved in methanol together with *N*-*n*-butyldiethanolamine (*N*-*n*-H_2_bda) and sodium azide (NaN_3_) as co-ligands. All reactants were used in an equimolar ratio. The resulting violet solution was stirred under reflux for 2 h and then worked up (see Experimental section for more details).

Compound **1**, [Co^II^${\text{Co}}_{4}^{\text{III}}$(N_3_)_4_(*N*-*n*-bda)_4_(bza·SMe)_2_]·H_2_O, forms in the presence of cobalt(II) acetate-tetrahydrate (Co(OOCMe)_2_·4H_2_O) or cobalt(II) sulfate-heptahydrate (CoSO_4_·7H_2_O). The dark-green, block-shaped crystals of neutral compound **1** are highly soluble in CHCl_3_, CH_2_Cl_2_, and tetrahydrofuran (THF); moderately soluble in acetone; and poorly soluble in MeOH, EtOH, and MeCN. The positive-mode electrospray ionization (ESI) mass spectrum of a methanol solution of **1** (see Supporting Information for details) contains peaks that correspond to the singly and quadruply charged sodium adduct ions (Maloth et al., 2015) [NaCo^II^${\text{Co}}_{4}^{\text{III}}$(N_3_)_4_(*N*-*n*-bda)_4_(bza·SMe)_2_]^+^ at *m*/*z* 1,456 and [Na_4_Co^II^${\text{Co}}_{4}^{\text{III}}$(N_3_)_4_ (*N*-*n*-bda)_4_(bza·SMe)_2_]^4+^ with 100% intensity at *m*/*z* 381, respectively. The signal of [${\text{Co}}_{4}^{\text{II}}$Co^III^(N_3_)_4_(*N*-*n*-bda)_3_(bza·SMe)_2_(H_2_O)(H^+^)_3_]^2+^ at *m*/*z* 647 and those of the coordination cluster ions with different amounts of azide groups [Co^II^${\text{Co}}_{4}^{\text{III}}$(N_3_)_3_(*N*-*n*-bda)_4_(bza·SMe)_2_]^+^ at *m*/*z* 1,391, [${\text{Co}}_{2}^{\text{II}}$${\text{Co}}_{3}^{\text{III}}$(N_3_)_2_(*N*-*n*-bda)_4_(bza·SMe)_2_]^+^ at *m*/*z* 1,349, [${\text{Co}}_{3}^{\text{II}}$${\text{Co}}_{2}^{\text{III}}$(N_3_)(*N*-*n*-bda)_4_(bza·SMe)_2_]^+^ at *m*/*z* 1,307, and [${\text{Co}}_{4}^{\text{II}}$Co^III^(*N*-*n*-bda)_4_(bza·SMe)_2_]^+^ at *m*/*z* 1,265 are also observed. As can be seen for the singly charged ions, cobalt(III) centers undergo partial reduction to Co^II^ upon loss of azides during the ionization process. This indicates that the [Co^II^${\text{Co}}_{4}^{\text{III}}$(N_3_)_4_(*N*-*n*-bda)_4_(bza·SMe)_2_] complex (**1**) could, in principle, be converted to a reduced {Co_5_}-based derivative. Elemental analysis of compound **1** emphasizes the stability of the complex against air and moisture, although **1** takes up a small amount of water during drying and storing under ambient conditions ([Co^II^${\text{Co}}_{4}^{\text{III}}$(N_3_)_4_(*N*-*n*-bda)_4_(bza·SMe)_2_]·1.75H_2_O vs. [Co^II^${\text{Co}}_{4}^{\text{III}}$(N_3_)_4_(*N*-*n*-bda)_4_(bza·SMe)_2_]·H_2_O as determined crystallographically).

Compound **2**, [${\text{Co}}_{4}^{\text{II}}$${\text{Co}}_{2}^{\text{III}}$(N_3_)_4_(*N*-*n*-bda)_2_ (bza·SMe)_5_(MeOH)_4_]Cl·2.25MeOH, forms when cobalt(II) chloride-hexahydrate (CoCl_2_·6H_2_O) was utilized. The dark-green, plate-like crystals of ionic compound **2** are soluble in CH_2_Cl_2_, THF, acetone, and MeCN; moderately soluble in MeOH and EtOH; and insoluble in water. The positive-mode ESI mass spectrometry pattern of a methanol solution of **2** (see Supporting Information for details) shows the signal at *m*/*z* 1,675 with 100% intensity, which is assigned to the singly charged ion of [${\text{Co}}_{4}^{\text{II}}$${\text{Co}}_{2}^{\text{III}}$(N_3_)_4_(*N*-*n*-bda)_2_(bza·SMe)_5_]^+^ after the loss of four coordinated MeOH molecules. Isotopic patterns reflecting reduction processes as in neutral complex **1** with terminal azide groups were not observed for ionic complex **2** with bridging azide groups, thus revealing **2** as less reactive. Elemental analysis of **2** indicates the stability of the central [${\text{Co}}_{4}^{\text{II}}$${\text{Co}}_{2}^{\text{III}}$(N_3_)_4_(*N*-*n*-bda)_2_(bza·SMe)_5_]^+^ molecular framework against air and moisture, although **2** loses the four weakly coordinated MeOH molecules and the uncoordinated crystal solvent by an uptake of water molecules during drying and storing under ambient conditions ([${\text{Co}}_{4}^{\text{II}}$${\text{Co}}_{2}^{\text{III}}$(N_3_)_4_(*N*-*n*-bda)_2_(bza·SMe)_5_]Cl·8H_2_O vs. [${\text{Co}}_{4}^{\text{II}}$${\text{Co}}_{2}^{\text{III}}$(N_3_)_4_(*N*-*n*-bda)_2_(bza·SMe)_5_(MeOH)_4_]Cl·2.25MeOH as determined crystallographically). The loss of the neutral coordinated solvent molecules was also identified in the ESI-MS pattern of **2**.

The reactivity of compounds **1** and **2** was tested under solvothermal conditions (Scheme 1). Each compound was dissolved in DMSO and the resulting green solutions were heated up to 100°C for 120 h in sealed autoclavable glass vials, followed by slow cooling to room temperature. Contrary to the reaction solution containing **1** yielding pink needle-shaped crystals of [${\text{Co}}_{10}^{\text{II}}$(N_3_)_2_(*N*-*n*-bda)_6_(bza·SMe)_6_] (**3**), that of compound **2** gave no crystalline product, although **2** can be strikingly compared to the previously synthesized coordination cluster [${\text{Co}}_{3}^{\text{II}}$${\text{Co}}_{2}^{\text{III}}$(*N*-*n*-bda)_2_(*N*-*n*-Hbda)_2_(ib)_6_] (ib^−^ = isobutyrate) (Schmitz et al., 2016a). Interestingly, the latter complex acts as an indispensable precursor material for the preparation of the previously reported complex [${\text{Co}}_{10}^{\text{II}}$(OH)_2_(*N*-*n*-bda)_6_(ib)_6_] (Schmitz et al., 2018b) that shows similarities to **3** (see Discussion of their molecular structures below). Thus, both ${\text{Co}}_{10}^{\text{II}}$ complexes can be solvothermally formed from mixed-valence ${\text{Co}}_{5}^{\text{II}/\text{III}}$ precursors in DMSO.

### X-Ray Crystal Structures

**Figure 1:**
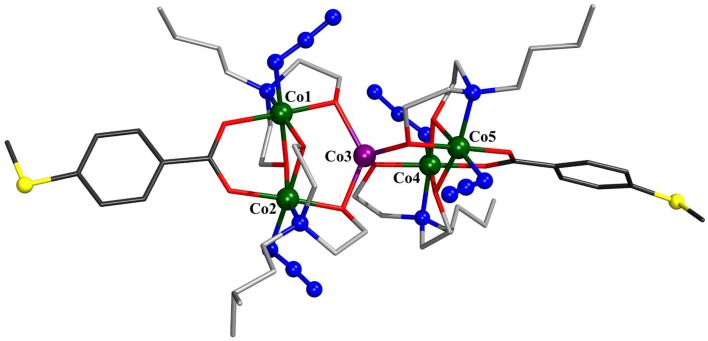
Molecular structure of [Co^II^${\text{Co}}_{4}^{\text{III}}$(N_3_)_4_(*N*-*n*-bda)_4_(bza·SMe)_2_] (**1**). Hydrogen atoms and co-crystallized water molecule are omitted for clarity. The cobalt, sulfur, and the nitrogen atoms of azide groups are represented as ball-and-stick models. Color code: C (bza·SMe^−^) = dark gray; C (*N*-*n*-bda^2−^) = gray; N = blue; O = red; S = yellow; Co^II^ = violet, Co^III^ = dark green. See Supporting Information for detailed crystal packing of the complexes.

The solid-state molecular structures of **1**–**3** were established by single-crystal X-ray diffraction (see Supporting Information for details). Compound **1** crystallizes in the monoclinic space group *P*2_1_/*c* (Figure 1). The structure consists of five cobalt atoms, where four of them reside in an octahedral N_2_O_4_ environment and exhibit a formal oxidation state of +III as established by bond valence sum (BVS) analysis [*Σ*_bv_(Co^III^) = 3.08–3.12]. The fifth cobalt site, located at the center of the molecule, displays a highly distorted tetrahedral geometry and is assigned the formal oxidation state +II [*Σ*_bv_(Co^II^) = 1.72]. The mixed-valent {Co_5_} core is thus similar to that observed in the other known Co_5_ complexes (Englert and Strähle, 1987; Ferguson et al., 2006; Funes et al., 2015; Li et al., 2017) and comprises two {${\text{Co}}_{2}^{\text{III}}$Co^II^} triangles that share a common Co^II^ vertex, an ~90° angle between the triangle planes. The Co^III^ coordination spheres are composed of one bza·SMe^−^, two *N*-*n*-bda^2−^, and two azide ligands. The bza·SMe^−^ ligand bridges two Co^III^ ions [Co^III^–O_bza·*SMe*_: 1.919(3)−1.925(3) ], and each azide coordinates terminally to every Co^III^ ion [Co^III^–N_azide_: 1.930(4)−1.940(3) ]. These Co^III^ centers are additionally bridged by oxygen atoms of ethoxide residues of two *N*-*n*-bda^2−^ ligands [Co^III^–O_bda_: 1.896(3)−1.932(3) ]. The other ethoxide groups of each *N*-*n*-bda^2−^ ligand link the peripheral Co^III^ ions with the central Co^II^ ion through their oxygen atoms [Co^III^–O_bda_: 1.867(3)−1.875(3); Co^II^–O_bda_: 1.996(3)−2.010(3) ]. The non-bonding Co^III^···Co^II^ distances range from 3.0834(0) to 3.1116(0), the nearest Co^III^···Co^III^ distance is 2.8499(7) (Co1···Co2), and the longest Co^III^···Co^III^ is 5.8833(1) (Co1···Co4). The intramolecular distance between two sulfur atoms is *ca*. 22.061. The thioether groups are not involved in any intermolecular coordinative bonds as it was also observed for other complexes with similar ligands (Ghisolfi et al., 2014; Schmitz et al., 2015, 2016b,c).

**Figure 2:**
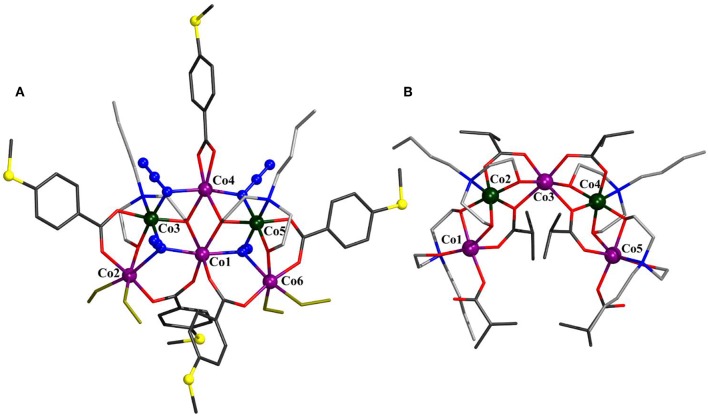
Molecular structure of [${\text{Co}}_{4}^{\text{II}}$${\text{Co}}_{2}^{\text{III}}$(N_3_)_4_(*N*-*n*-bda)_2_(bza·SMe)_5_(MeOH)_4_]^+^ in **2 (A)** and comparison to [${\text{Co}}_{3}^{\text{II}}$${\text{Co}}_{2}^{\text{III}}$(*N*-*n*-bda)_2_(*N*-*n*-Hbda)_2_(ib)_6_] (Schmitz et al., 2016a) **(B)**. Hydrogen atoms, Cl^−^ counterion, and co-crystallized solvent molecules are omitted for clarity. The cobalt, sulfur, and the nitrogen atoms of azides are represented as ball-and-stick models. Color code: C (carboxylates) = dark gray; C (*N*-*n*-Hbda^−^ and *N*-*n*-bda^2−^) = gray; N = blue; O = red; S = yellow; coordinated MeOH molecules = yellow sticks; Co^II^ = violet; Co^III^ = dark green. See Supporting Information for detailed crystal packing of the complexes.

Compound **2** crystallizes in the orthorombic space group *Pbca* and represents an ionic compound consisting of the hexanuclear [Co_6_(N_3_)_4_(*N*-*n*-bda)_2_(bza·SMe)_5_(MeOH)_4_]^+^ complex charge-balanced by a Cl^−^ ion (Figure 2). Five cobalt atoms exhibiting alternating oxidation states +II and +III [*Σ*_bv_(Co^II^) = 1.9–2.0, *Σ*_bv_(Co^III^) = 3.1] form a semicircular or horseshoe-shaped metal core structure reminiscent of that previously described complex [${\text{Co}}_{3}^{\text{II}}$${\text{Co}}_{2}^{\text{III}}$(*N*-*n*-bda)_2_(*N*-*n*-Hbda)_2_(ib)_6_] (Schmitz et al., 2016a) (see Supporting Information for a comparative analysis). However, in **2**, this structural motif is complemented with a centrally accommodated, sixth cobalt(II) atom. The cationic part of compound **2** is supported by five 4-(methylthio)benzoate ligands, two fully deprotonated *N*-*n*-butyldiethanolamine ligands, four azide ions, and four methanol molecules. The presence of the latter suggests interesting derivatization perspectives for this type of cobalt structural motifs unlike the reported [${\text{Co}}_{3}^{\text{II}}$${\text{Co}}_{2}^{\text{III}}$(*N*-*n*-bda)_2_(*N*-*n*-Hbda)_2_(ib)_6_] complex. All cobalt ions in **2** adopt an octahedral geometry. The reduced Co2 and Co6 reside in a NO_5_, the oxidized Co3 and Co5 reside in a N_3_O_3_, and the reduced Co1 and Co4 reside in a N_2_O_4_ environment. The central Co1 center is connected to Co2 and Co6 through the μ_3_-azide groups [Co1–μ_3_-N_azide_: 2.203(6) and 2.216(7) ] and the carboxylate μ_2_-COO^−^ groups of the bza·SMe^−^ ligand [Co1–O_bza·*SMe*_: 1.974(5) and 2.005(5) ]. It is further bridged to Co3 and Co5 *via* the abovementioned μ_3_-azide groups [Co3–μ_3_-N_azide_: 1.982(6) and Co5–μ_3_-N_azide_: 1.970(6) ] and the μ_3_-oxygen atoms of the ethoxide groups of the *N*-*n*-bda^2−^ ligands, which also coordinate to Co4 [Co–μ_3_-O_bda_: 1.907(5)−2.148(5) ]. In addition, Co4 is linked to Co3 and Co5 by bridging azide groups [Co–μ_2_-N_azide_: 1.947(6)−2.063(7) ]. The coordination sphere of Co4 is saturated by one chelating bza·SMe^−^ ligand [Co4–O_bza·*SMe*_: 2.078(5) and 2.164(5) ]. The coordination sphere of each Co3 and Co5 is completed by a μ_2_-oxygen atom of the ethoxide group of the *N*-*n*-bda^2−^ ligand that also connects to Co2 or Co6 [Co–μ_2_-O_bda_: 1.882(5)−2.013(5) ], and by a nitrogen atom of this ligand [Co3–N_bda_: 1.991(6) and Co5–N_bda_: 1.994(6) ]. The structure displays four coordinated methanol molecules where two of them are attached terminally to Co2 and the other two are attached to Co6 [Co–O_MeOH_: 2.060(6)−2.122(5) ]. The peripheral thioether groups are not involved in any intermolecular coordinative bonds (Ghisolfi et al., 2014; Schmitz et al., 2015, 2016b,c).

**Figure 3:**
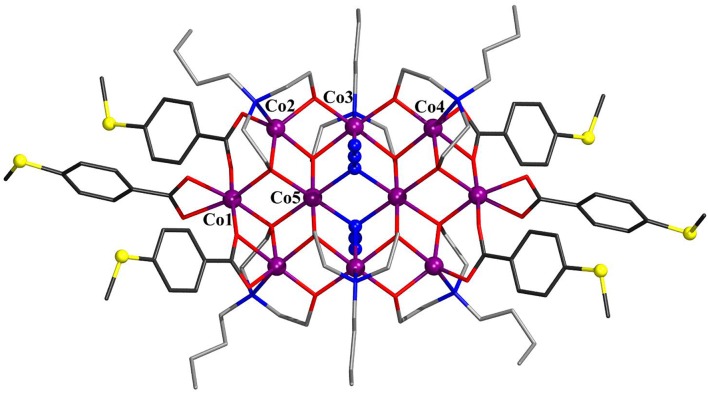
Molecular structure of [${\text{Co}}_{10}^{\text{II}}$(N_3_)_2_(*N*-*n*-bda)_6_(bza·SMe)_6_] (**3**) with Co numbering scheme for one asymmetric unit. Hydrogen atoms are omitted for clarity. The cobalt, sulfur, and the nitrogen atoms of azides are represented as ball-and-stick models. Color code: C (carboxylates) = dark gray; C (*N*-*n*-bda^2−^) = gray; N = blue; O = red; S = yellow; Co^II^ = violet. See Supporting Information for detailed crystal packing of the complexes.

Compound **3** crystallizes in the monoclinic space group *P*2_1_/*n* (Figure 3) and displays strong structural similarities to the previously reported [${\text{Co}}_{10}^{\text{II}}$(OH)_2_(*N*-*n*-bda)_6_(ib)_6_] (Schmitz et al., 2018b). The main differences between these two neutral coordination clusters concern the two discrete OH^−^ groups that in the latter are replaced by two bridging ${\text{N}}_{3}^{-}$ ligands [Co–N_azide_: 2.212(3) and 2.293(4) ] and the ib ligands, replaced by bza·SMe^−^ in **3** [Co–O_bza·SMe_: 2.028(3)−2.296(3) ]. Contrary to [${\text{Co}}_{10}^{\text{II}}$(OH)_2_(*N*-*n*-bda)_6_(ib)_6_] with only octahedral cobalt centers, compound **3** features eight octahedral and two (Co2 and Co2′) NO_4_-trigonal bipyramidal Co environments. These five-coordinated Co^II^ centers bind only one carboxylic oxygen of bza·SMe^−^ [2.028(3) ], while the Co···O distance for the second center is too long for the bond [2.722(3) ]. For comparison, the corresponding distances in the known [${\text{Co}}_{10}^{\text{II}}$(OH)_2_(*N*-*n*-bda)_6_(ib)_6_] complex are about 0.3 shorter and constitute 2.363(3) and 2.466(3). In contrast, the similar Co–O_bza·SMe_ distance for Co4 is much shorter [2.296(3) ] and is just slightly above the Co4–O_bza·SMe_ distance to the second O of the same ligand [2.160(3) ]. Other structurally characterized decanuclear cobalt coordination clusters are also available (Canaj et al., 2014).

### Thermal Treatment of 1

**Figure 4:**
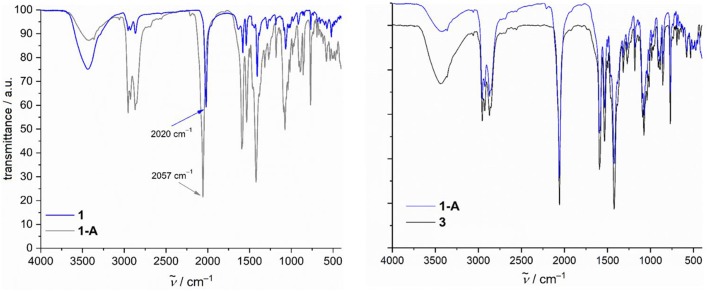
Comparison of IR spectra of compounds **1** vs. **1-A** (left) and **1-A** vs. **3** (right).

Guided by the ESI-MS spectrum and the TG/DTA curve of compound **1** measured in the temperature range 25–800°C (see Supporting Information for details), this coordination cluster was grinded into the dark-green powder that was selectively heated up to 220°C for 15 min under a nitrogen flow. An exothermic step in the range 170–220°C was observed, corresponding to a weight loss (Δ*m*_found_) of 11.95% that is assigned to two C_2_H_3_O^−^ groups (two ethynol and one H_2_) from the *N*-*n*-bda^2−^ ligands and to two terminal azide groups (Δ*m*_calcd._ = 11.86%). The loss of not only N_2_ from azides is also supported by thermal mass-spectrometry measurements (see Supporting Information for details). The IR spectrum of the thermally-generated, dark-violet compound **1-A** illustrated in Figure 4 still shows the presence of azide groups, which points out that not all of these ligands were released during the thermal processing of compound **1**. The band shift from 2,020 cm^−1^ (asymmetric stretching vibrations of the terminal coordinated –N = N^+^ = N^−^ groups in **1**) to 2,057 cm^−1^ (in **1-A**) indicates that the remaining azide ligands change their coordination mode from terminal to bridging (see Supporting Information for detailed description of IR spectra). This Δṽ shift of 37 cm^−1^ is in line with the reported Δṽ values describing the terminal vs. bridging ${\text{N}}_{3}^{-}$ coordination (Ray et al., 2003; Mandal et al., 2017). In addition to this, the elemental analysis of compound **1-A** reveals *ca*. 10% of nitrogen (see Experimental section), which reflects the loss of only two azide groups. Although the IR spectra of compounds **1-A** and **3** are almost identical as seen in Figure 4, these compounds exhibit different colors (dark violet vs. pink, respectively) and magnetochemical behavior (see Discussion below). Given that the asymmetric stretching vibration band of azides in **3** appears at 2,058 cm^−1^, the rearrangement of these ligands in **1-A** thus are likely.

**Scheme 2:**
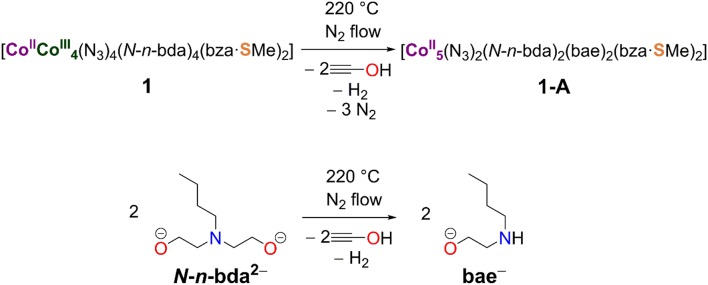
Structural redox transformation of compound **1** to compound **1-A** accompanied by the release of two azide groups and the conversion of the *N*-*n*-bda^2−^ ligand to bae^−^.

The acquired data from TG/DTA, IR, and CHNS analyses were used to propose the structural redox modification route toward **1-A** (Scheme 2). The electronic and magnetic characteristics of this compound were further studied by X-ray photoelectron spectroscopy (XPS) and the SQUID method.

### XPS Spectrum of Compounds 1 and 1-A

**Figure 5:**
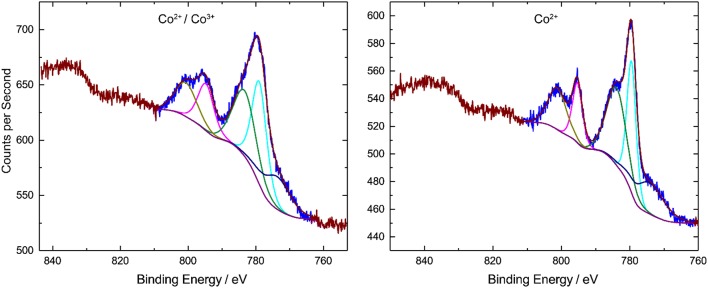
XPS spectra of compounds **1** (left) and **1-A** (right) measured in the solid state. Compound **1** features the central, high-spin Co(II) ion and the peripheral, low-spin Co(III) ions. In compound **1-A**, the portion of high-spin Co(II) ions is however significantly increased.

Figure 5 shows the XPS spectra of bulk **1** and **1-A**. The binding energy of Co 2p_3/2_ peak is 779.0 eV for **1** and 779.5 eV for **1-A**. The Co 2p_1/2_ peak is shifted by 15.8 eV to higher energies. These values are in line with literature data (Oku and Hirokawa, 1976; Shi et al., 2012). An Auger peak was detected at ~774 eV (Wagner, 1972). The XPS spectra of both compounds shows satellite features of Co 2p_3/2_ (shifted by 4.4 eV) and Co 2p_1/2_ (shifted by 5.9 eV), respectively. The satellite peaks in the Co 2p region are due to high-spin Co^2+^ ions, whereas low-spin Co^3+^ ions have no or very weak satellite peaks (Frost et al., 1974; Shi et al., 2012). To distinguish the configuration of the Co centers, the ratio between areas of Co 2p peak and the satellite is determined. For compound **1**, the Co 2p:satellite area ratio is around 1, for compound **1-A** the ratio decreases to 0.8. This means that, for compound **1-A**, the satellite peak is stronger and more high-spin Co^2+^ ions are present.

### Magnetism and Magnetochemical Modeling

**Figure 6:**
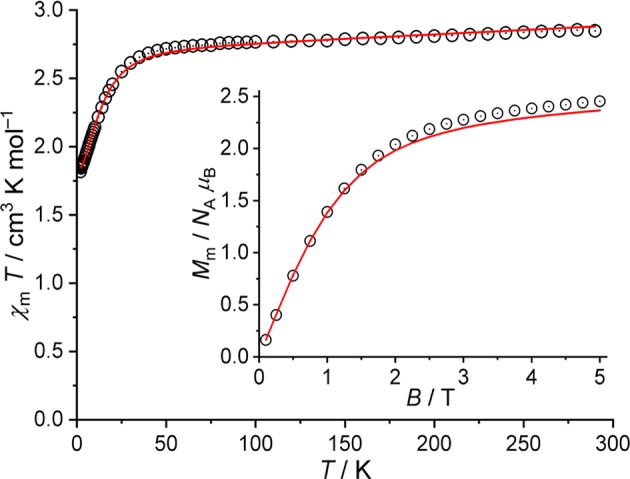
Magnetic data of **1**: Temperature dependence of χ_m_*T* at *B* = 0.1 T; inset: molar magnetization *M*_m_ vs. applied field *B* at *T* = 2.0 K (open circles: experimental data, straight lines: calculated data).

As the magnetic ac susceptibility data did not reveal significant out-of-phase signals at low temperatures, the magnetic properties of compounds **1** and **2** are analyzed in terms of their magnetic dc susceptibility. The magnetic data of **1** are shown as χ_m_*T* vs. *T* and *M*_m_ vs. *B* plots in Figure 6. These data are corrected for diamagnetic contributions as well as the temperature-independent paramagnetic (TIP) contributions originating from the four octahedrally coordinated low-spin Co^III^ centers. The value of χ_m_*T* = 2.87 cm^3^ K mol^−1^ at 290 K is within the range 2.31–3.38 cm^3^ K mol^−1^ expected for a single high-spin Co^II^ center (Lueken, 1999). Upon lowering the temperature, χ_m_*T* remains almost constant and marginally decreases down to a temperature of 50 K. This spin-like behavior is due to the orbital singlet ^4^*A*_2_ ground term of tetrahedrally coordinated 3d^7^ centers. The strong distortion of the tetrahedral coordination geometry is revealed at temperatures smaller than 50 K, at which χ_m_*T* rapidly decreases, reaching 1.83 cm^3^ K mol^−1^ at 2.0 K. The characteristics of this interval are due thermal depopulation of the energy states, namely, the splitting of the ^4^*A*_2_ ground term (into two Kramers doublets) caused primarily by ligand field effects and spin-orbit coupling. This is also reflected in the dependence of the molar magnetization on the applied magnetic field at 2.0 K. The molar magnetization *M*_m_ is a linear function of *B* up to *ca*. 1 T, and reaches ~2.5 *N*_A_ μ_B_ at 5 T without being saturated. Therefore, magnetic data are in agreement with a single high-spin Co^II^ center (3d^7^) in a distorted tetrahedral ligand field.

The computational framework CONDON (Schilder and Lueken, 2004; Speldrich et al., 2011, 2018) was used to analyze the magnetic data of **1** in more detail. Standard values (Griffith, 1971) for the Racah parameters (*B* = 1,115 cm^−1^, *C* = 4,366 cm^−1^) and the one-electron spin-orbit coupling constant (ζ_3d_ = 533 cm^−1^) are used to analyze the Co^II^ center using the “full” basis of microstates of a d^7^ valence electron configuration (120 microstates). The least-squares fit with an *SQ* of 1.5% (relative root mean square error) yields the ligand field parameters $B_{0}^{2}$ = (−15,460 ± 250) cm^−1^, $B_{0}^{4}$ = (−9,782 ± 411) cm^−1^, $B_{4}^{4}$ = (−4,634 ± 512) cm^−1^ (Wybourne notation, *D*_2d_ symmetric ligand field) and λ_mf_ = (65 ± 1) × 10^−3^ mol cm^−3^. The magnitude of the molecular field parameter λ_mf_ corresponds to minimal intermolecular ferromagnetic exchange interactions (*zJ* ≈ 0.02–0.03 cm^−1^), which has been introduced due to the slight change of the curvature of χ_m_*T* for *T* < 4 K, and can be considered as negligible. The modeled data are displayed in Figure 6 as solid lines. According to the fit parameters, the ligand field is represented by a strongly compressed tetrahedron. The tetrahedral ^4^*A*_2_ ground term of the free ion splits into two Kramers doublets, which are separated by an energy gap of 32 cm^−1^. The second excited doublet (derived from the ^4^*T*_2_ term of the undistorted tetrahedral symmetry) is located at 2,100 cm^−1^ relative to the ground doublet, revealing a good isolation of the two ground doublets in agreement with the observed spin-like behavior of χ_m_*T* in the temperature range 50–290 K.

**Figure 7:**
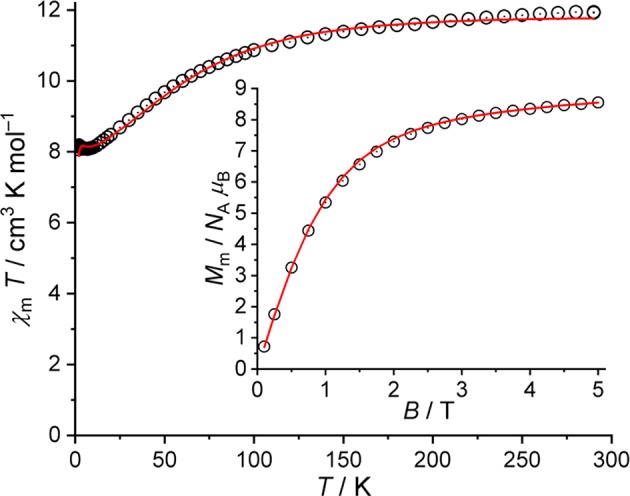
Magnetic data of **2**: Temperature dependence of χ_m_*T* at *B* = 0.1 T; inset: molar magnetization *M*_m_ vs. applied field *B* at *T* = 2.0 K (open circles: experimental data, straight lines: calculated data).

The χ_m_*T* vs. *T* and *M*_m_ vs. *B* plots of **2** are shown in Figure 7. These data are corrected for diamagnetic contributions and TIP contributions of the two octahedrally coordinated low-spin Co^III^ centers. At 290 K, the value of χ_m_*T* is 11.93 cm^3^ K mol^−1^, which is well within the range 9.25–13.53 cm^3^ K mol^−1^ expected (Lueken, 1999) for four non-interacting high-spin Co^II^ centers. Upon cooling the compound, χ_m_*T* gradually decreases with a distinct change of the slope at about 100 K, reaching a minimum of 8.08 cm^3^ K mol^−1^ at 7.0 K. Subsequently, χ_m_*T* slightly increases to a maximum of 8.18 cm^3^ K mol^−1^ at 2.4 K, and drops to 8.07 cm^3^ K mol^−1^ at 2.0 K. At this temperature, the molar magnetization rapidly increases for fields below 2 T, and subsequently slowly grows to 8.6 *N*_A_ μ_B_ at 5.0 T without reaching saturation. (Distorted) octahedral Co^II^ centers are not spin-like, but show a temperature-dependent χ_m_*T* behavior due to considerable contributions from the unquenched orbital momentum. This is mainly due to a significant mixing of the ^4^*T*_1g_(^4^*F*) ground term with the excited ^4^*T*_1g_(^4^*P*) term caused by spin-orbit coupling. Therefore, the decrease of χ_m_*T* with decreasing temperatures is basically due to these single-ion effects, and only to a minor degree due to potential exchange interactions. The existence of the latter can be derived from the occurrence of the minimum, which is indicative of ferromagnetic exchange interactions. However, these interactions are weak, since the minimum is at quite low temperatures, and the increase upon further cooling is rather small. Additionally, even weaker antiferromagnetic exchange interactions could be present based on the small drop off of χ_m_*T* at *T* ≤ 2.4 K, although also the Zeeman effect yields a relevant contribution to χ_m_*T* at 0.1 T in this temperature range.

For **2**, we adopt the same values for the Racah parameters *B* and *C* as well as the one-electron spin-orbit coupling constant ζ_3d_ as for **1**, using the “full” basis of microstates of the d^7^ valence electron configuration per Co^II^ center. Due to structural information, the ligand field of each center is approximated to *C*_4v_ symmetry, and all four centers are taken to be roughly the same. For the exchange interactions pathways, we set the exchange interactions between Co6–Co1 and Co6–Co5 to *J*_1_ (Co6 is the central Co^II^ center, Co1 and Co5 are the peripheral Co^II^ in a NO_5_-coordination environment), and between Co6–Co3 to *J*_2_. For the calculation of the exchange interactions, the basis is reduced to the 12 energy states of the ^4^*T*_1g_ term for each center. The least-squares fit (*SQ* = 0.9%) yields the ligand field parameters $B_{0}^{2}$ = (−7,957 ± 136) cm^−1^, $B_{0}^{4}$ = (24,378 ± 126) cm^−1^, $and\;B_{4}^{4}$ = (19,403 ± 139) cm^−1^, and the exchange interaction parameters *J*_1_ = (0.38 ± 0.04) cm^−1^ and *J*_2_ = (−0.29 ± 0.03) cm^−1^ (Heisenberg-Hamiltonian in “−2*J*” notation). Hence, the ligand field parameters describe the field of an elongated octahedron as an approximation for all four Co^II^ centers. Two exchange pathways are characterized by weak ferromagnetic exchange interactions (*J*_1_), while the remaining pathway is characterized by a weak antiferromagnetic exchange interaction (*J*_2_). The corresponding fit is shown as a solid line in Figure 7.

**Figure 8:**
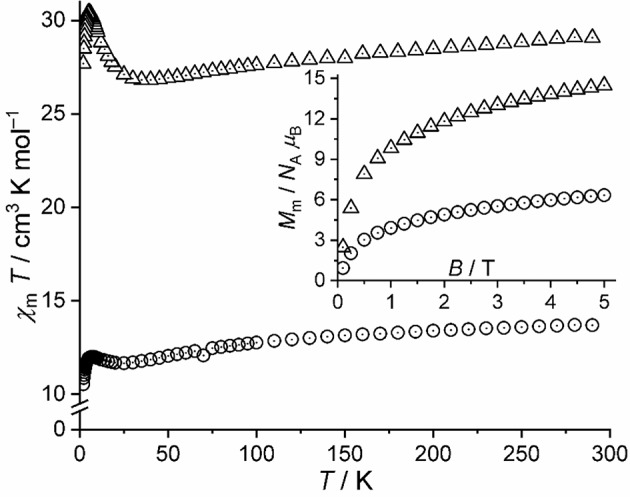
Magnetic data of **1-A** and **3**, for comparison: Temperature dependence of χ_m_*T* at *B* = 0.1 T; inset: molar magnetization *M*_m_ vs. applied field *B* at *T* = 2.0 K (open circles: **1-A**, open triangles: **3**).

The magnetic properties of **1-A** (compound **1** after thermal treatment) are shown as χ_m_*T* vs. *T* and *M*_m_ vs. *B* plots in Figure 8 (open circles). The curves of **1** and **1-A** distinctly differ from each other: The χ_m_*T* value of **1-A** at 290 K is 13.69 cm^3^ K mol^−1^, which represents a value well within the expected range 11.56–16.91 cm^3^ K mol^−1^ for five non-interacting high-spin Co^II^ centers (Lueken, 1999) assuming a similar or nearly similar structure of **1-A** as for **1**. Upon decreasing temperature, χ_m_*T* continuously decreases with a distinct change of the slope at about 100 K characteristic for octahedrally coordinated high-spin Co^II^ centers. At lower temperatures, we observe a minimum at 25 K, a maximum of 12.00 cm^3^ K mol^−1^ at 7.0 K, and subsequently a sharp drop to 10.53 cm^3^ K mol^−1^ at 2.0 K. Therefore, the χ_m_*T* vs. *T* curve below 30 K reveals the presence of weak or moderate exchange interactions of both kinds: Ferromagnetic due to the observed minimum, and antiferromagnetic due to the pronounced drop off for *T* < 7.0 K. The molar magnetization at 2.0 K rapidly increases at fields below 0.5 T and continues to increase to 6.3 *N*_A_ μ_B_ at 5.0 T without reaching saturation, which is characteristic of high-spin octahedral Co^II^ centers.

Given the striking similarities in the IR spectra of **1-A** and **3** (Figure 4), we show the χ_m_*T* vs. *T* curve and *M*_m_ vs. *B* curve of **3** in addition to the data of **1-A** in Figure 8. At 290 K, χ_m_*T* of **3** is 29.08 cm^3^ K mol^−1^, well with the expected (Lueken, 1999) range of 23.12–33.81 cm^3^ K mol^−1^ for 10 non-interacting high-spin Co^II^ centers. Upon cooling the compound, the values of χ_m_*T* almost linearly decrease to a minimum at 40 K, sharply increases to a maximum of 30.44 cm^3^ K mol^−1^ at 5.0 K, and subsequently drops to 27.69 cm^3^ K mol^−1^ at 2.0 K. The molar magnetization at this temperature sharply increases at fields up to 0.8 T, and subsequently increases to 14.5 *N*_A_ μ_B_ at 5.0 T. The data of **3** are, thus, in agreement with 10 high-spin Co^II^ centers, which interact predominantly ferromagnetic, while a few exchange pathways are characterized by antiferromagnetic exchange interactions. In comparison to **1-A**, the data of **3**, however, differ significantly by more than a factor of two, show the characteristic minimum and maximum of the χ_m_*T* curves at different temperatures with differing intensities, and have different slopes of the *M*_m_ curves at *B* > 2 T. Since also the maxima and slopes differ by a factor of two, compounds **1-A** and **3** have most likely slightly different structures and comprise different ligands, or a combination of both alternatives is present from a magnetochemical point of view.

### Large-Area Charge Transport Measurements

We succeeded in growing SAMs of the target compounds **1** and **2** by immersing freshly cleaved template-stripped gold substrates (atomically smooth Au^TS^) in a ~0.1 mM methanolic solution of each metal complex overnight. After rinsing with pure methanol and drying in a gentle stream of nitrogen, the SAMs were contacted with EGaIn tips to form junctions of the structure Au^TS^//SAM//Ga_2_O_3_/EGaIn where “/” denotes interface defined by chemisorption and “//” denotes interface defined by physisorption. Note that EGaIn is a eutectic alloy of Ga and In (75.5% Ga and 24.5% In by weight), the surface of which is covered by thin, conductive, self-limiting layer of Ga_2_O_3_.

**Figure 9:**
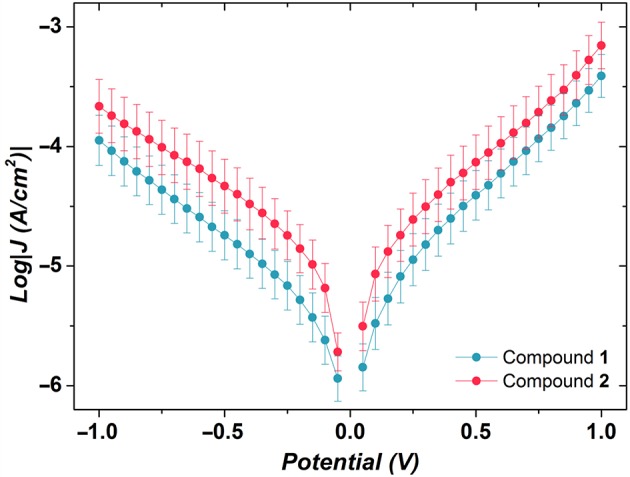
Plots of logarithmic current-density against applied potential for the SAMs of compounds **1** and **2**. Values of log|*J*| at *V* = 0 V are omitted for clarity. The error bars are the 95% confidence intervals. Despite the minor difference in the shape of the *J*/*V* curves, the two SAMs are indistinguishable.

We performed an analysis of the current density–voltage (*J–V*) characteristics of the thus-obtained SAMs. The *J–V* results are illustrated in Figure 9. Apart from minor differences in the shape of *J*/*V* curves (*J* = *I*/*A*, where *A* is the area of the junction), the two SAMs are indistinguishable. By replotting *J–V* data in Fowler-Nordheim coordinates (transition voltage spectroscopy), it is possible to obtain information about energy level alignment inside the junction. All values of transition voltages (*V*_T_) coalesce to ~0.6 V (Table 1), ~0.3 V higher than those reported recently for the SAMs of {CuLn} complexes (Schmitz et al., 2018a). The featureless *J–V* curves shown in Figure 9 point out single-level, non-resonant tunneling, i.e., a single potential barrier and comparably weak coupling to either electrode.

**Table 1:** Transition voltages of compounds **1** and **2** calculated at negative and positive biases.

	**${\text{V}}_{\text{T}}^{-}$ (V)**	**${\text{V}}_{\text{T}}^{+}$ (V)**
Compound **1**	−0.56 ± 0.06	0.43 ± 0.05
Compound **2**	−0.61 ± 0.07	0.50 ± 0.04

The errors are the 95% confidence intervals.

*V*_T_ is an approximate measure of the height of the potential barrier, which is related to level alignment, for example, the difference between the energy of the accessible frontier orbital of a molecule and the Fermi level of the electrode in an assembled junction. If a rectangular tunneling barrier is considered, the heights of the barriers in the SAMs of Co complexes are higher than those of {CuLn} complexes (Schmitz et al., 2018a).

**Figure 10:**
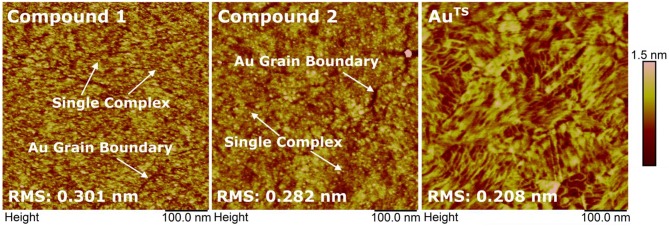
Surface morphology of the SAMs of compounds **1** and **2** and of the template-stripped Au substrate (from left to right) measured by AFM.

Similar to the SAMs of {CuLn} complexes, Co complexes described herein can only weakly interact with the metal surface through physisorption. Thus, we expect the SAMs to be poorly ordered and the molecules are weakly coupled to the bottom electrode that is to exhibit high resistance. AFM study of the surface morphology clearly showed the presence of compounds **1** and **2**. All molecules formed monolayers on the surface of Au^TS^ and there was no sign of the multilayer formation (Figure 10). Moreover, the formation of SAMs increases the surface roughness of the samples, from 0.208 nm for bare Au^TS^ substrate to 0.301 nm for compound **1** and 0.282 nm for compound **2**. The obtained monolayers with an average yield of working EGaIn junctions of 91% are more electrically robust than the SAMs of previously reported {CuLn} complexes (Schmitz et al., 2018a). This could be due to the fact that more Co complexes are generated on the surface of Au, largely reducing the space between the molecules, which contributes short contacts.

### Thickness Measured by Using Ellipsometry

To further characterize the SAMs grown from the target compounds, we measured the thickness of the SAMs by using ellipsometry. Three freshly prepared samples for each compound were analyzed. The average thickness is 1.50 ± 0.03 nm for compound **1** and 1.62 ± 0.03 nm for compound **2** (Table 2). It is thus in good agreement with the dimensions of the complexes (Table 2) and suggests the formation of monolayers from the studied compounds. Moreover, no presence of aggregates that exceed the size of a single complex from the morphology measured by AFM and STM was observed. Although we could not conclude the orientation of the complexes on the Au surface due to the relatively flexible ligands, the formation of SAMs from these two complexes can be ascertained.

**Table 2:** Thickness of the SAMs of compounds **1** and **2** by ellipsometry, in comparison to the dimensions of the complexes.

	**Thickness** **(nm)**	**Length** **(nm)**	**Width** **(nm)**	**Height** **(nm)**
Compound **1**	1.50 ± 0.03	2.319	1.291	1.291
Compound **2**	1.62 ± 0.03	1.916	2.132	1.337

## Conclusion

Two novel coordination clusters, pentanuclear **1** and hexanuclear **2**, comprising high-spin Co^II^ and low-spin Co^III^ ions in the ligand environment of 4-(methylthio)benzoate ligands, fully deprotonated *N*-*n*-butyldiethanolamine ligands and azide ligands were synthesized and structurally characterized. Contrary to **2**, the charge-neutral compound **1** was shown to undergo reduction under solvothermal conditions to decanuclear {${\text{Co}}_{10}^{\text{II}}$}-based compound **3**. Another derivative **1-A** with only high-spin Co^II^ ions was isolated after thermogravimetric treatment of **1**, although its structure could not be elucidated due to the lack of availability of single crystals suitable for X-ray diffraction. The molecular deposition of compounds **1** and **2** from solution on gold surfaces resulted in the formation of physisorbed SAMs that revealed higher electrical robustness than SAMs consisting of the previously reported {Cu^II^Ln^III^} complexes (Schmitz et al., 2018a). Further studies are however needed to showcase the potential of polynuclear cobalt complexes to switch between different stable metal-based oxidation or spin states by application of external stimuli (Xin et al., 2019) as temperature, light, or magnetic fields.

## Data Availability Statement

All datasets generated for this study are included in the article/Supplementary Material.

## Author Contributions

All authors listed have made a substantial, direct and intellectual contribution to the work, and approved it for publication.

### Conflict of Interest

The authors declare that the research was conducted in the absence of any commercial or financial relationships that could be construed as a potential conflict of interest.

## Footnotes

^1^CrysAlisPro, Agilent Technologies, Version 1.171.36.28 (release 01-02-2013 CrysAlis171.NET) (compiled Feb 1 2013, 16:14:44) (Clark and Reid, 1995).

^2^APEX2 (Version 1.08), SAINT (Version 7.03), SADABS (Version 2.11). Bruker Advanced X-Ray Solutions, Madison (WI, USA).
