# Supplementary material for: Conductive Self-Assembled Monolayers of Paramagnetic {CoIICo4III} and {Co4IICo2III} Coordination Clusters on Gold Surfaces
Source: Front Chem. 2019 Nov 5;7:681. doi: 10.3389/fchem.2019.00681 (PMC6848059; doi:10.3389/fchem.2019.00681)
Supplement: Supplementary file 1 [file Data_Sheet_2.pdf]

Supporting Information

Conductive Self-Assembled Monolayers of Paramagnetic $\{Co^{II}Co^{III}_4\}$ and $\{Co^{II}_4Co^{III}_2\}$ Coordination Clusters on Gold Surfaces

Sebastian Schmitz,[1,5] Xinkai Qiu,[2] Maria Glöß,[1,3] Jan van Leusen,[1] Natalya V. Izarova,[3] Muhammad Arif Nadeem,[4] Jan Griebel,[5] Ryan C. Chiechi,[2*] Paul Kögerler,[1,3*] and Kirill Yu. Monakhov[5*]

[1] Institut für Anorganische Chemie, RWTH Aachen University, Landoltweg 1, 52074 Aachen, Germany

[2] Stratingh Institute for Chemistry & Zernike Institute for Advanced Materials, University of Groningen, Nijenborgh 4, Groningen 9747 AG, Netherlands

[3] Jülich-Aachen Research Alliance (JARA-FIT), Peter Grünberg Institute, Forschungszentrum Jülich GmbH, Wilhelm-Johnen-Straße, 52425 Jülich, Germany

[4] Department of Chemistry, Quaid-i-Azam University, 45320 Islamabad, Pakistan

[5] Leibniz Institute of Surface Engineering (IOM), Permoserstr. 15, 04318 Leipzig, Germany

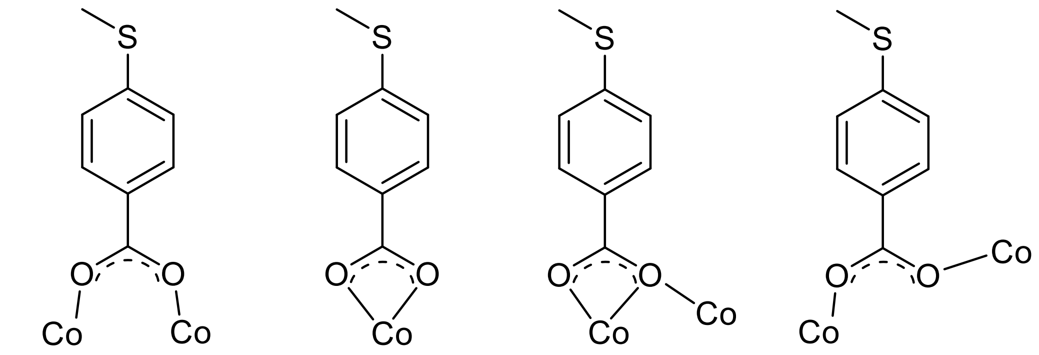

in **1** and **2** — *syn*,*syn*-$\eta^1:\eta^1:\mu_2$

in **2** and **3** — chelating

in **3** — $\eta^2:\eta^1:\mu_2$

in **3** — *syn*,*anti*-$\eta^1:\eta^1:\mu_2$

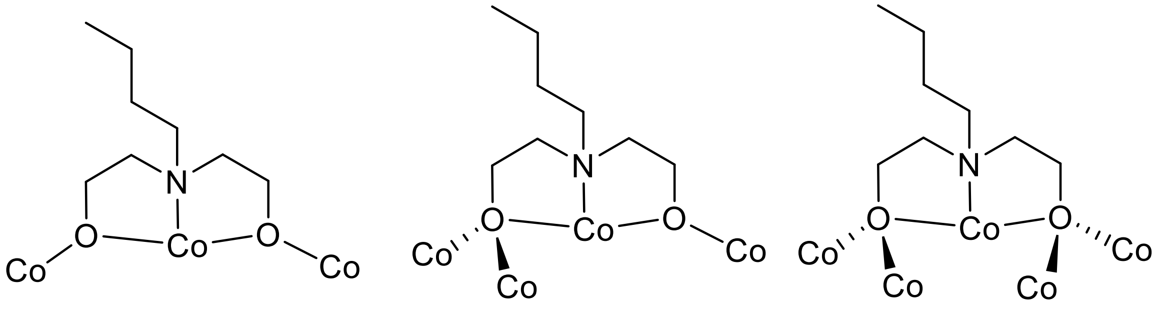

in **1** — $\eta^2:\eta^2:\eta^1:\mu_3$

in **2** and **3** — $\eta^3:\eta^2:\eta^1:\mu_4$

in **3** — $\eta^3:\eta^3:\eta^1:\mu_5$

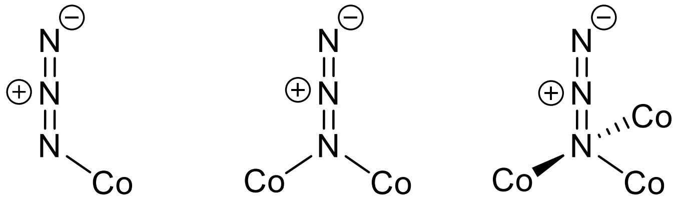

in **1** — μ-1

in **2** — μ_2-1,1

in **2** and **3** — μ_3-1,1,1

Scheme S1. Coordination modes of the ligands in compounds **1**–**3**.

Table S1. Crystal data and structure refinement details for compounds **1–3.**

Sample	**1**	**2**	**3**
Empirical formula	$C_{48}H_{84}Co_5N_{16}O_{13}S_2$	$C_{62.25}H_{94}ClCo_6N_{14}O_{20.25}S_5$	$C_{96}H_{144}Co_{10}N_{12}O_{24}S_6$
Formula weight / g mol^{-1}	1452.08	1911.84	2631.88
Crystal system	Monoclinic	Orthorhombic	Monoclinic
Space group	$P2_1/c$	*Pbca*	$P2_1/n$
a / Å	20.2361(2)	16.2281(2)	8.9930(6)
b / Å	22.46255(17)	31.8717(8)	16.5801(11)
c / Å	14.04427(13)	34.3157(8)	37.429(3)
β	104.889(1)°	90°	90.3330(10)°
Volume / Å^3	6169.55(10)	17748.7(7)	5580.7(6)
Z	4	8	2
D_{calc} / g cm^{-3}	1.563	1.431	1.566
Absorption coefficient / mm^{-1}	1.456	1.312	1.631
F(000)	3020	7892	2724
Crystal size / mm^3	0.143 × 0.245 × 0.306	0.054 × 0.214 × 0.265	0.080 × 0.090 × 0.240
Theta range for data collection	2.913° – 25.242°	3.456° – 25.027°	2.043° – 25.398°
Completeness to Θ_{max}	99.8 %	99.7 %	95.0 %
Index ranges	$-27 < h < 28$, $-31 < k < 30$, $-19 < l < 19$	$-19 < h < 19$, $-37 < k < 37$, $-39 < l < 40$	$-10 < h < 10$, $-20 < k < 19$, $-45 < l < 45$
Reflections collected	273651	88713	54893
Independent reflections	16943	15629	9754
R_{int}	0.0886	0.1498	0.1247
Observed ($I > 2\sigma(I)$)	11919	9158	5273
Absorption correction	Analytical numeric using a multifaceted crystal model		Semi-empirical from equivalents
T_{min} / T_{max}	0.5030 / 0.7230	0.6040 / 0.8750	0.5579 / 0.7452
Data / restraints / parameters	16943 / 56 / 765	15629 / 12 / 1013	9754 / 33 / 667
Goodness-of-fit on F^2	1.029	1.043	0.776
R_1, wR_2 ($I > 2\sigma(I)$)	R_1 = 0.0660, wR_2 = 0.1622	R_1 = 0.0786, wR_2 = 0.1772	R_1 = 0.0423, wR_2 = 0.0767
R_1, wR_2 (all data)	R_1 = 0.1040, wR_2 = 0.1888	R_1 = 0.1441, wR_2 = 0.2116	R_1 = 0.0884, wR_2 = 0.0828
Largest diff. peak and hole / e Å^{-3}	1.603 / –1.819	1.289 / –0.885	1.039 / –0.650

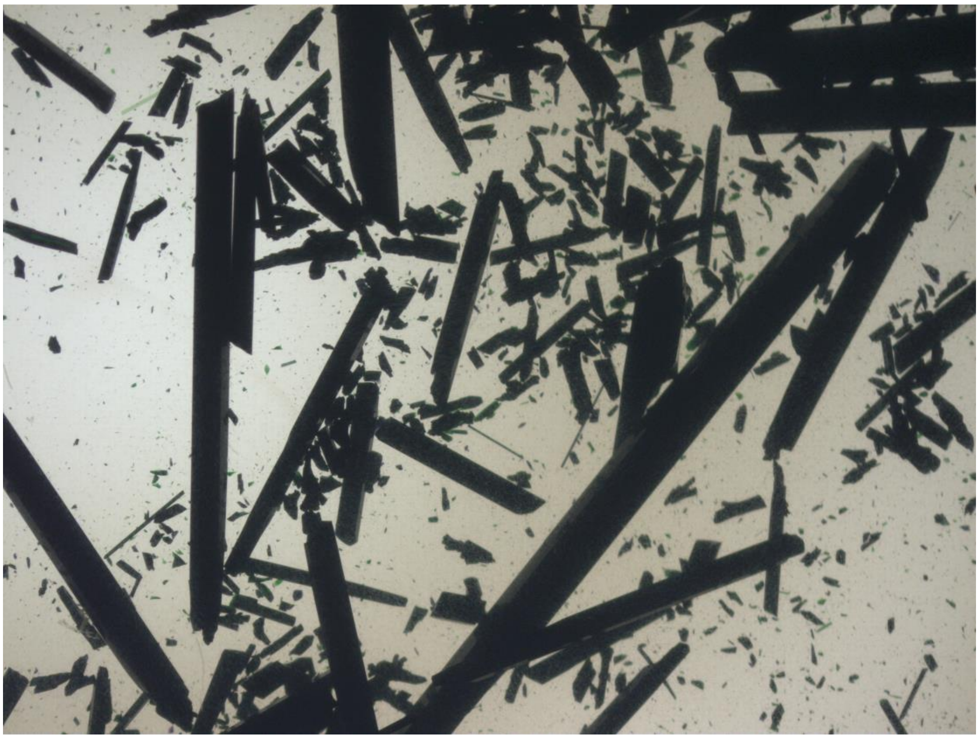

Figure S1. Photograph of the dark-green crystals of compound **1**.

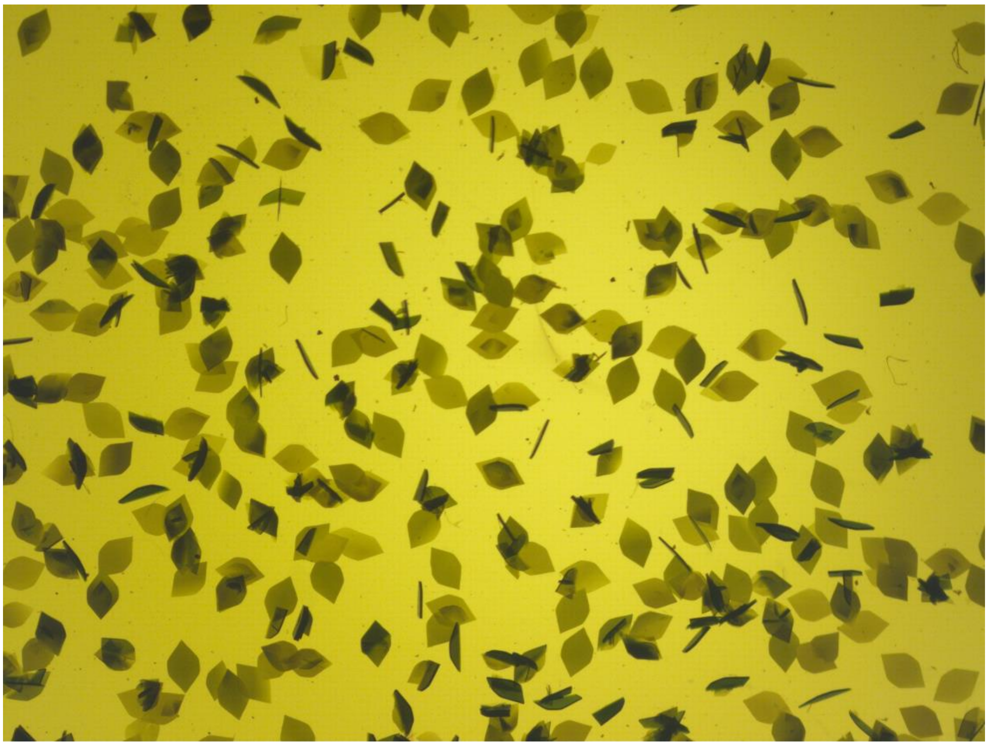

Figure S2. Photograph of the dark-green crystals of compound **2**.

Complexes [$Co_5(N_3)_4$(*N*-*n*-bda)$_4$(bza·SMe)$_2$] in the crystals of **1** are linked to each other by a complex network of hydrogen bonds and form imaginary chains directed along the crystallographic *a* axis (**Figure S3**), which are further packed in a hexagonal ABABA-mode (**Figure S6**). The connection of the molecules within the chains is achieved by hydrogen bonding between the CH_2 groups of the *N*-*n*-bda^{2-} ligands and the aromatic rings of the 4-(methylthio)benzoate groups (see the dashed cyan-blue lines in **Figures S3** and **S4**) as well as their sulfur atoms (shown as the brown dashed lines in **Figures S3** and **S4**). The distances between the C atom of the corresponding CH_2 group and the center of the π-system are in the range from 3.879 Å to 4.033 Å, and the corresponding C···S distances are 3.668–3.738 Å. The intermolecular interactions between the neighboring chains include both C–H···π bonds between the terminal CH_3 groups of the *N*-*n*-bda^{2-} ligands and the benzene rings of the 4-(methylthio)benzoate groups (3.558–4.362 Å) as well as the hydrogen bonds formed by hydrogen atoms of the CH_2 groups of the same *N*-*n*-bda^{2-} ligands and the nitrogen atoms of azides (see the green dashed lines in **Figures S4** and **S5**, C···N 3.652–3.907 Å). There are no close S···S contacts or π-π stacking interactions in this structure.

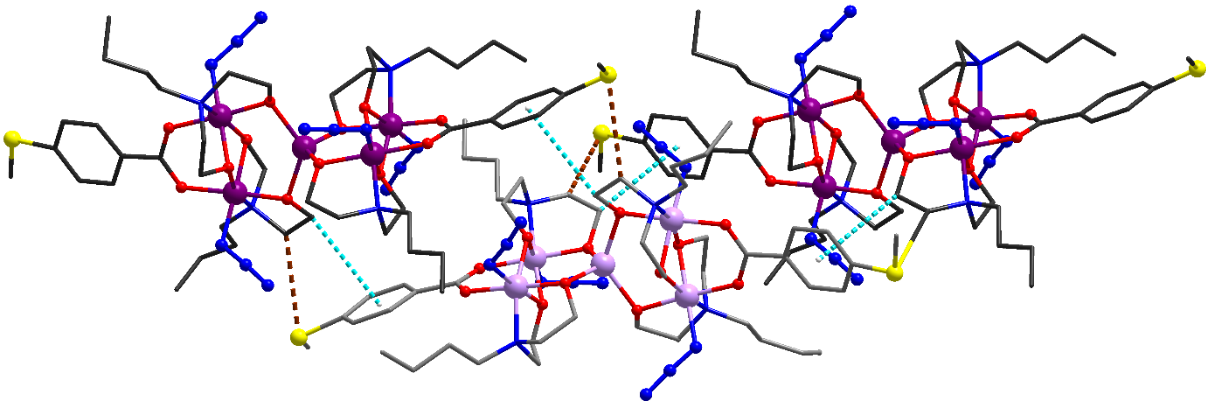

Figure S3. Intermolecular interactions within the chains formed along the crystallographic *a* axis in **1**. Color code: Co = violet/lavanda; O = red; N = blue; S = yellow spheres; C = black or gray (to better distinguish the organic groups of the neighboring species). C–H···π and C–H···S interactions are highlighted by dashed cyan-blue and brown lines, respectively.

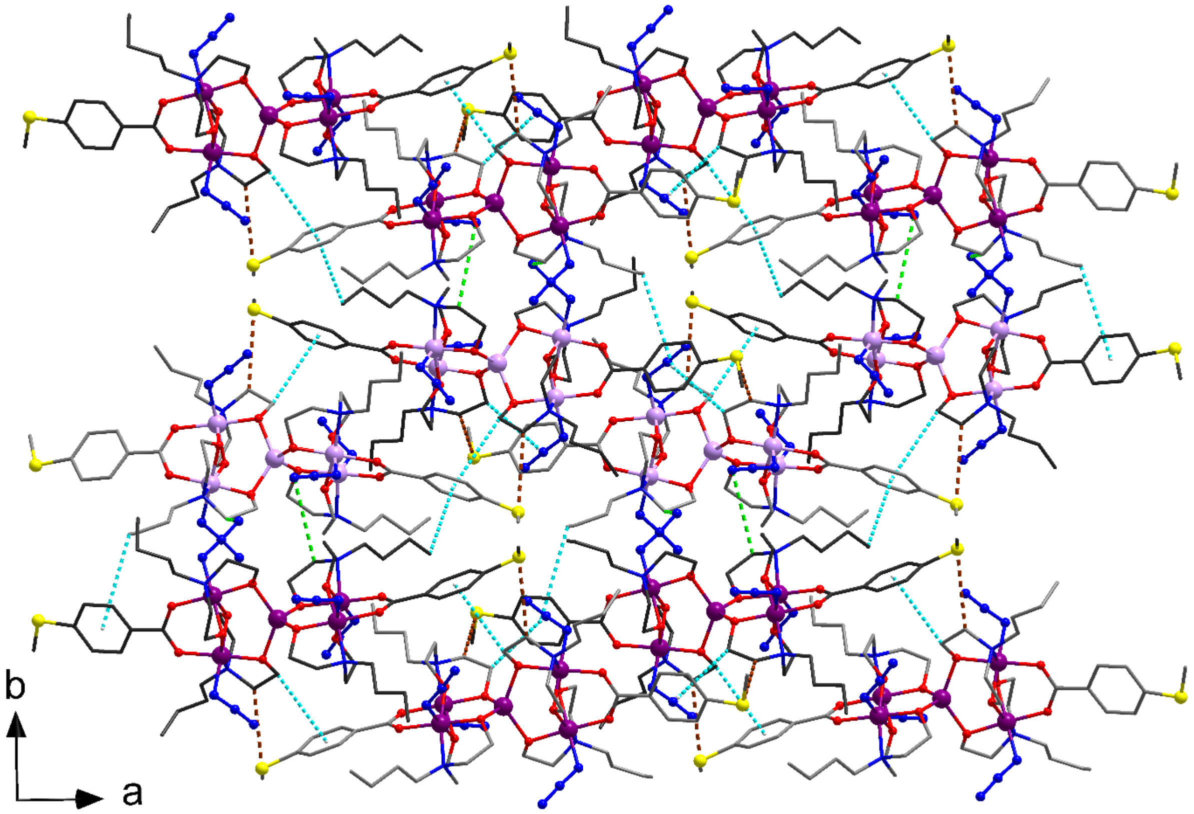

Figure S4. Crystal packing of the [$Co^{II}Co^{III}_4(N_3)_4$(*N*-*n*-bda)$_4$(bza·SMe)$_2$] complexes in **1**, view along the crystallographic *c* axis. Color code as in **Figure S3**. The Co atoms within one chain have the same color. C−H···π, C−H···S and C−H···N interactions are highlighted by dashed cyan-blue, brown and green lines, respectively. Solvent molecules are omitted for clarity.

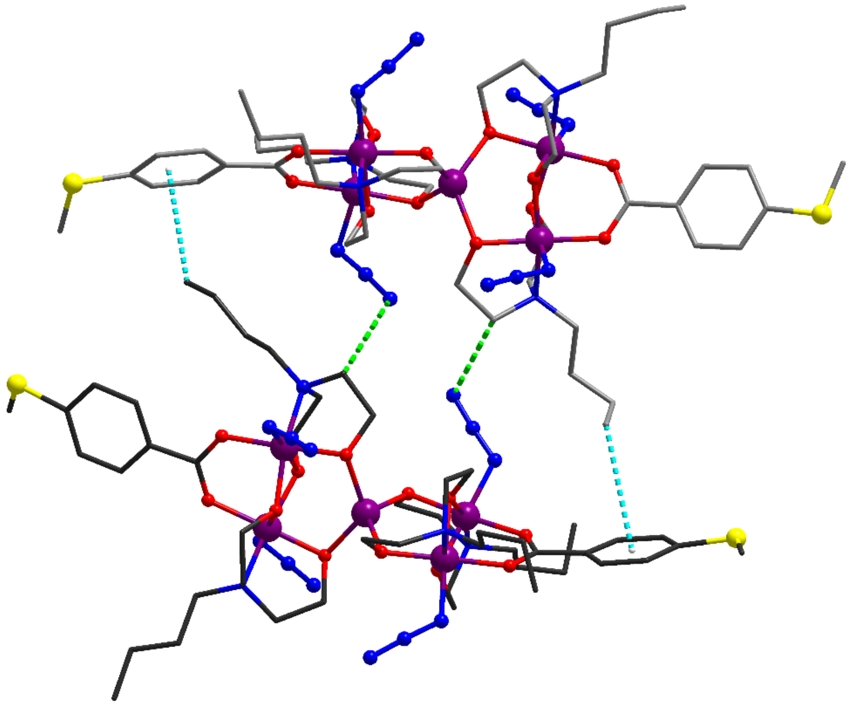

Figure S5. Connection between the closest [$Co^{II}Co^{III}_4(N_3)_4$(*N*-*n*-bda)$_4$(bza·SMe)$_2$] complexes from the neighboring chains in **1**. Color code as in **Figure S3**.

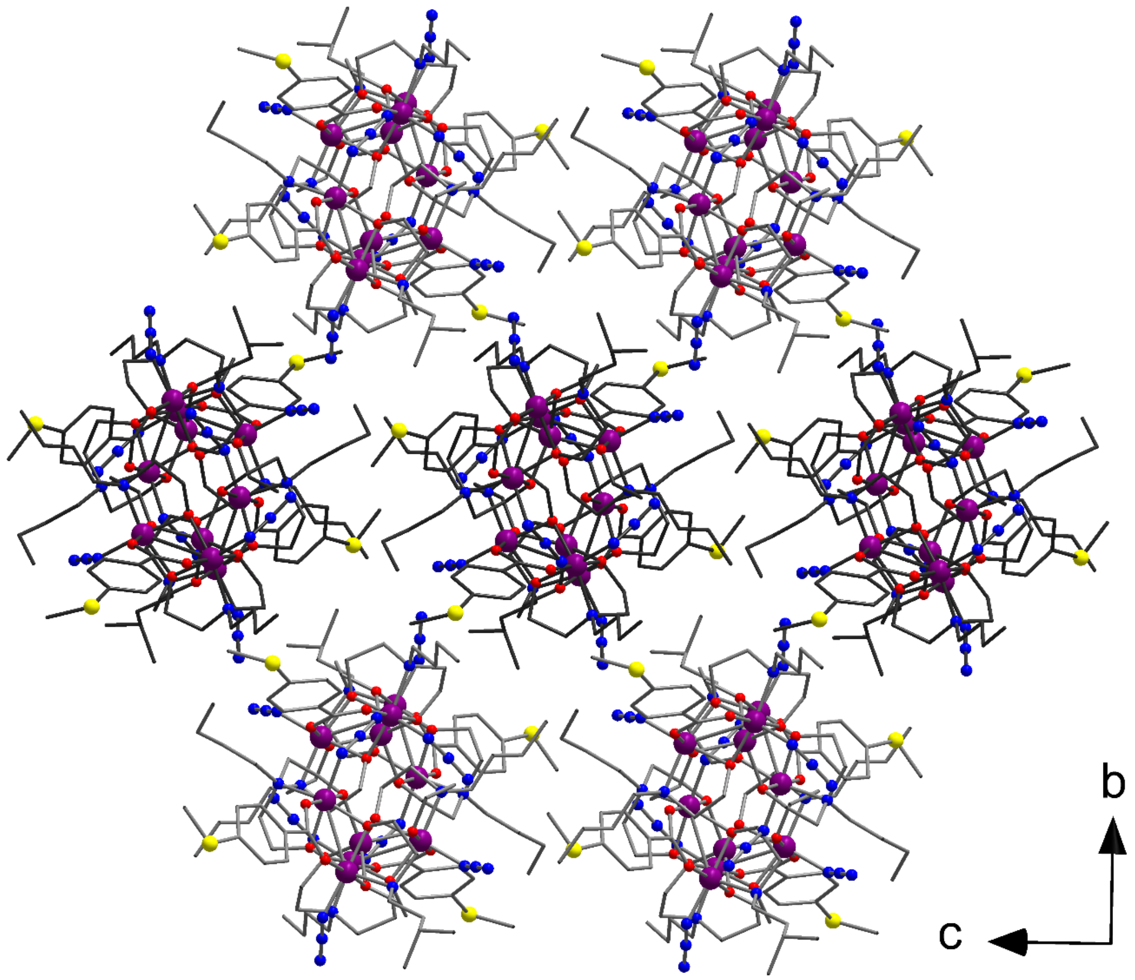

Figure S6. The packing of the chains in **1**, view along the crystallographic *a* axis. Color code as in **Figure S3**. Solvent molecules are omitted for clarity.

In the crystals of $[Co_6(N_3)_4(N\text{-}n\text{-bda})_2(bza{\cdot}SMe)_5(MeOH)_4]Cl$ (**2**) there is a developed network of week intermolecular C−H···π interactions between CH_3 groups and the aromatic rings of the 4-(methylthio)benzoate ligands (see dashed cyan-blue lines in **Figures S7** and **S8**). The distances between the C atom of the CH_3 group and the center of the corresponding π-system are in the range from 3.601 Å to 4.121 Å. There are also relatively short contacts between the S atoms of the 4-(methylthio)benzoate ligands and the C−H bonds of the aromatic rings in the neighboring molecule along the crystallographic *b* axis (C···S 3.550 Å, see **Figure S7**). In addition, there are close intermolecular S···S contacts (3.530 Å) directed along the crystallographic *a* axis, highlighted by pink dashed lines in **Figure S9**. The Cl^- ions and solvent molecules are placed in the voids between $[Co^{II}_4Co^{III}_2(N_3)_4(N\text{-}n\text{-bda})_2(bza{\cdot}SMe)_5(MeOH)_4]^+$ complexes.

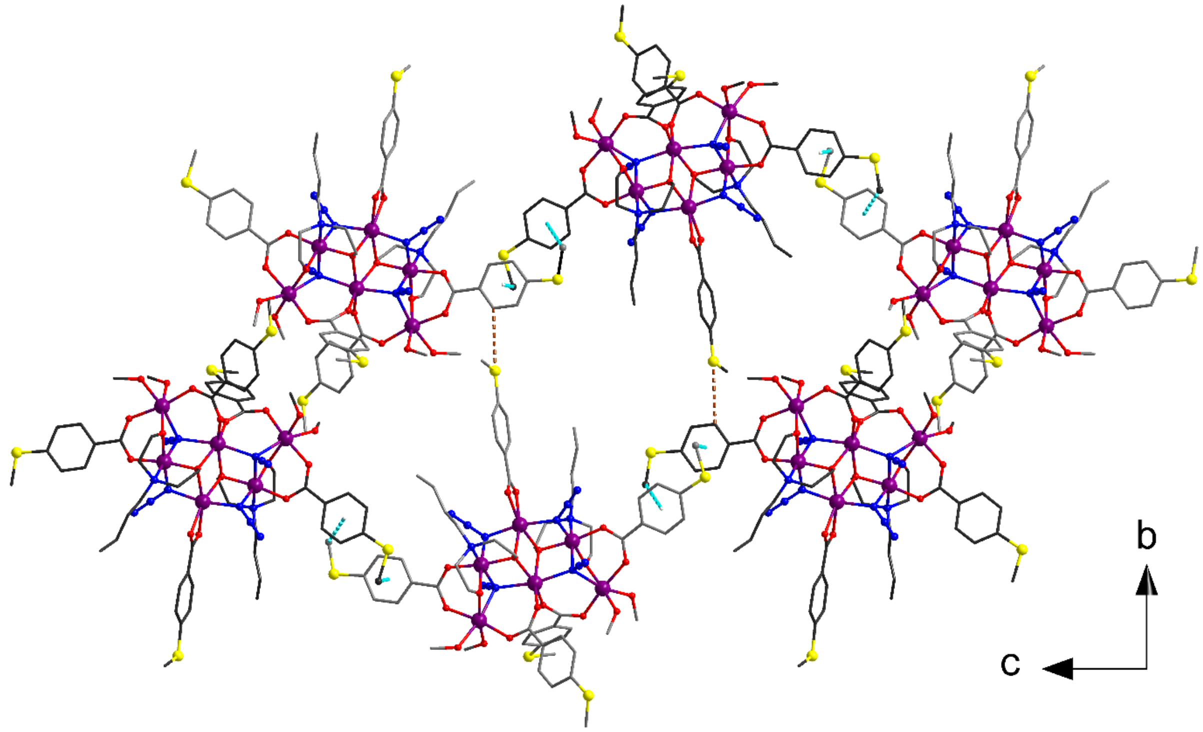

Figure S7. A layer of the $[Co^{II}_4Co^{III}_2(N_3)_4(N\text{-}n\text{-bda})_2(bza{\cdot}SMe)_5(MeOH)_4]^+$ complexes formed along the crystallographic *a* axis. Color code: Co = violet; O = red; N = blue; S = yellow spheres; C = black or gray (to better distinguish the organic groups of the neighboring species). C−H···π and C−H···S interactions are highlighted by dashed cyan-blue and brown lines, respectively. Cl^- ions and solvent methanol molecules are omitted for clarity.

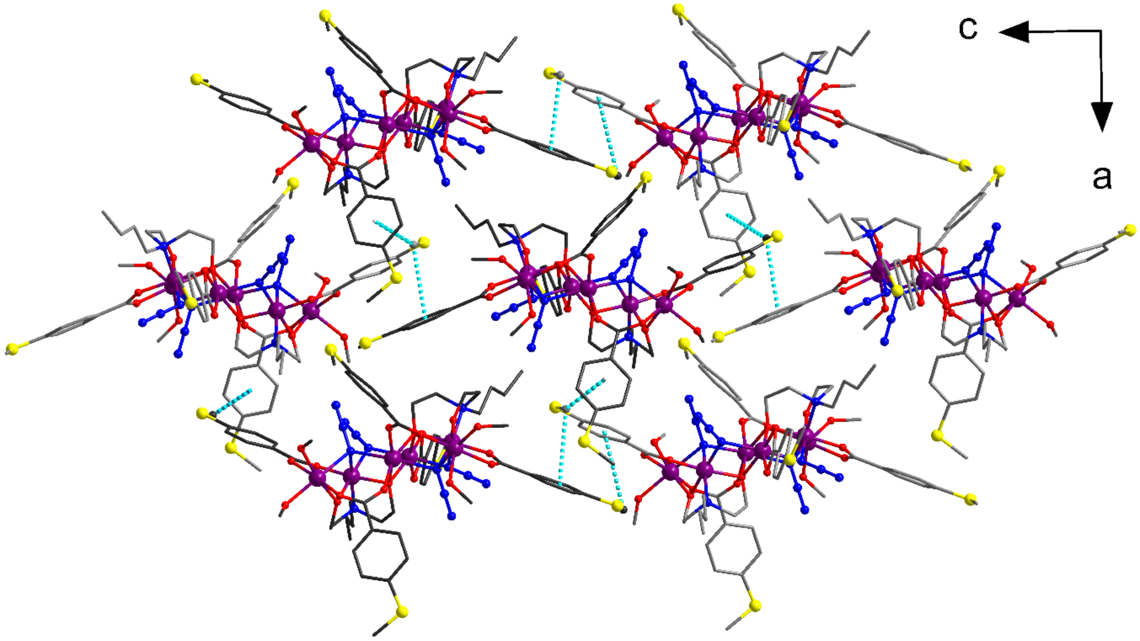

Figure S8. A layer of the $[Co^{II}_4Co^{III}_2(N_3)_4(N\text{-}n\text{-bda})_2(\text{bza}\cdot\text{SMe})_5(\text{MeOH})_4]^+$ complexes formed along the crystallographic *b* axis. Color code as in **Figure S7**. C–H···π interactions are highlighted by dashed cyan-blue lines. Cl^- ions and solvent methanol molecules are omitted for clarity.

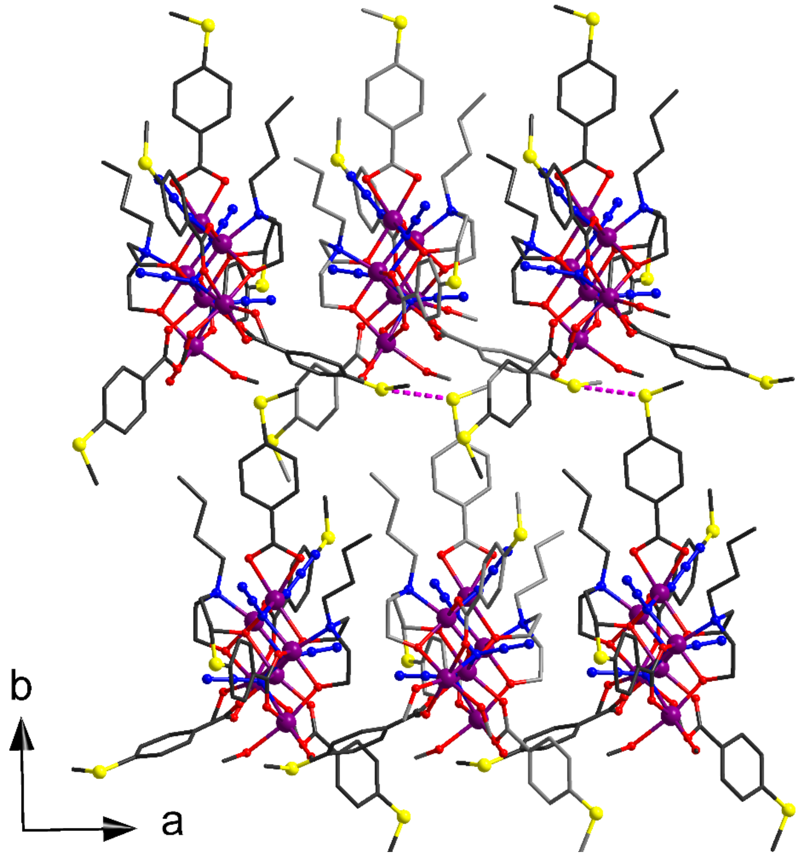

Figure S9. A layer of the $[Co^{II}_4Co^{III}_2(N_3)_4(N\text{-}n\text{-bda})_2(\text{bza}\cdot\text{SMe})_5(\text{MeOH})_4]^+$ complexes formed along the crystallographic *c* axis. Color code as in **Figure S7**. Close S···S contacts (3.530 Å) are highlighted by dashed pink lines. Cl^- ions and solvent methanol molecules are not shown.

In the crystal packing of $[Co_{10}(N_3)_2(N\text{-}n\text{-bda})_6(bza{\cdot}SMe)_6]$ (**3**) one can distinguish imaginary zigzag layers (**Figure S10**) which are arranged along the crystallographic *a* axis. The connection of the molecules within the layers are achieved due to C–H···π interactions between CH_3 and C–H groups and the aromatic rings of the 4-(methylthio)benzoate ligands (see dashed cyan-blue lines in **Figure S10**) for which the distances between the corresponding C atom and the center of the benzene ring are in the range from 3.009 to 3.919 Å. The interactions between the layers is very complex and include (1) H bonds between the terminal N atoms of the azide moieties and H atoms of the CH_2 groups of the N-n-bda^{2-} ligands (C···N 3.353 Å, brown dashed lines in **Figure S11**); (2) C–H···π interactions between the aromatic rings of the 4-(methylthio)benzoate ligands and the CH_3 and CH groups of the same type ligands as well as CH_2 groups of the N-n-bda^{2-} units (C···center of the benzene ring 3.350–3.919 Å, dashed cyan-blue lines in **Figure S11**), and (3) the relatively close S···S contacts of 4.387 Å (dashed pink lines in **Figure S11**).

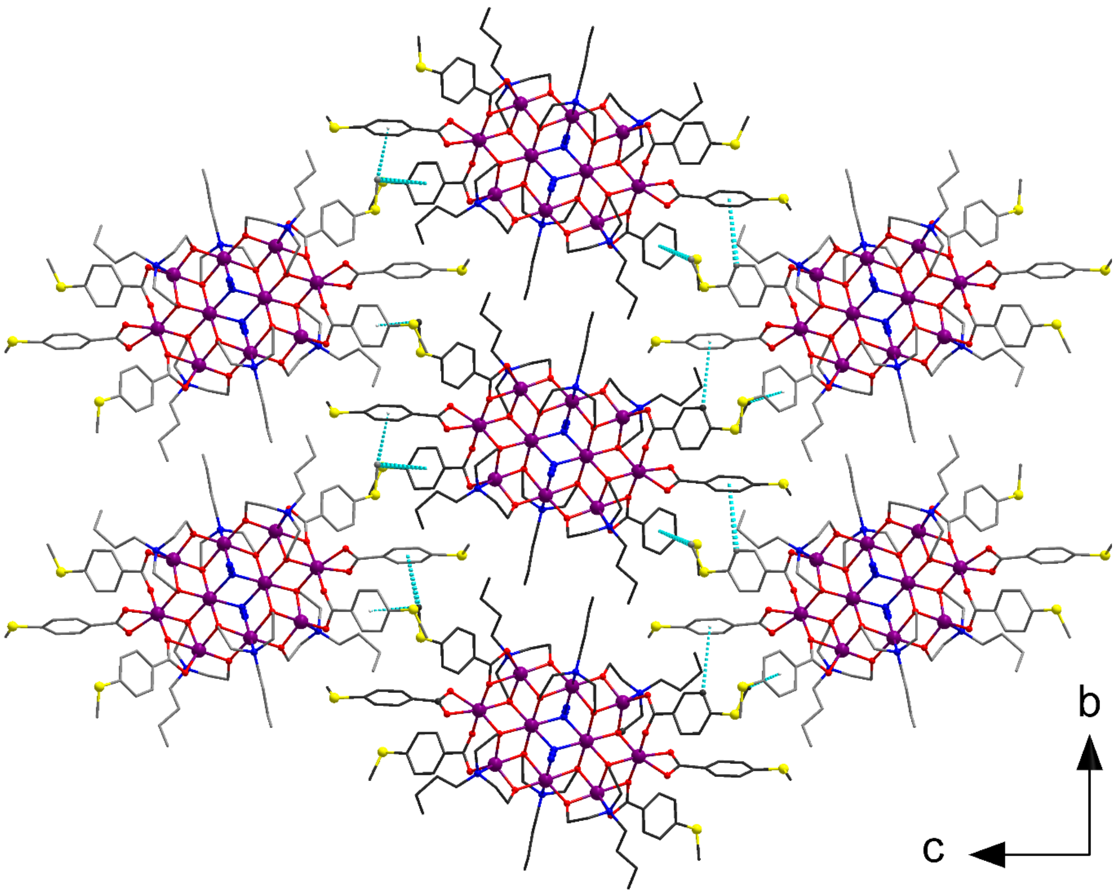

Figure 10. A zigzag layer of the $[Co^{II}_{10}(N_3)_2(N\text{-}n\text{-bda})_6(bza{\cdot}SMe)_6]$ complexes formed along the crystallographic *a* axis. Color code: Co = violet; O = red; N = blue; S = yellow spheres; C = black or gray (to better distinguish the organic groups of the neighboring molecules). C–H···π interactions are highlighted by dashed cyan-blue lines.

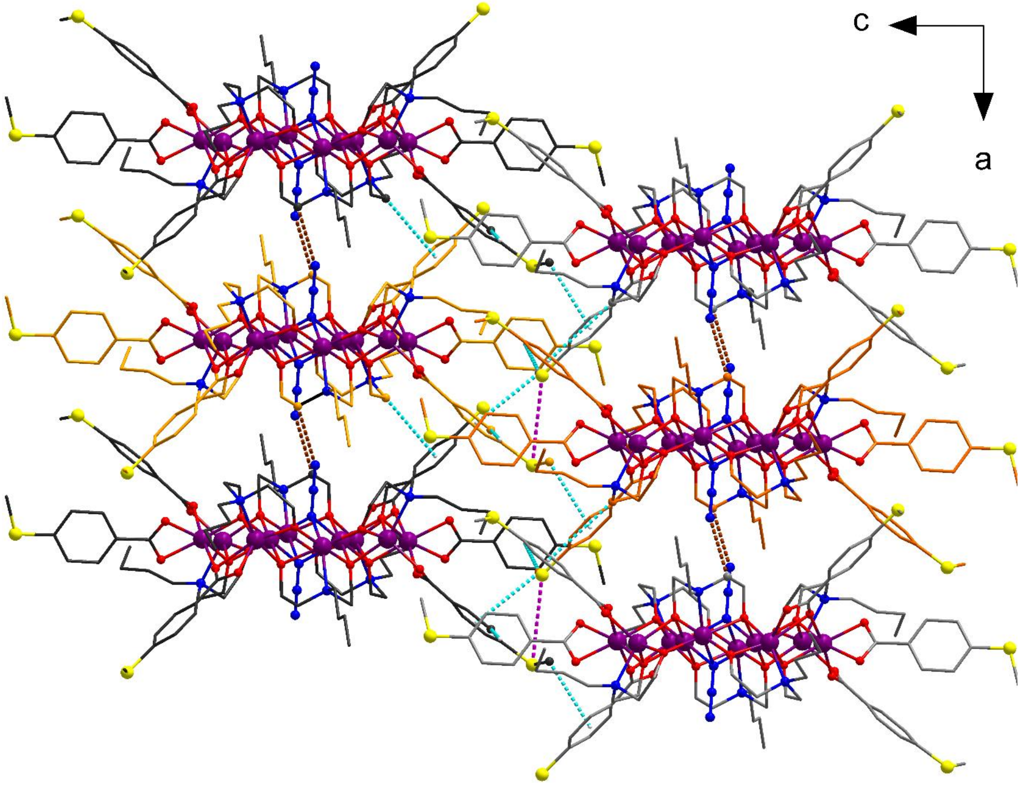

Figure S11. Crystal packing of **3** along the crystallographic *b* axis. Color code as in **Figure S10**. C atoms of the alternating zigzag layers are shown in gray / black and yellow / orange for better clarity. C−H···π and C−H···N interactions are highlighted by dashed cyan-blue and brown lines, respectively. The relatively close S···S contacts are shown as dashed pink lines.

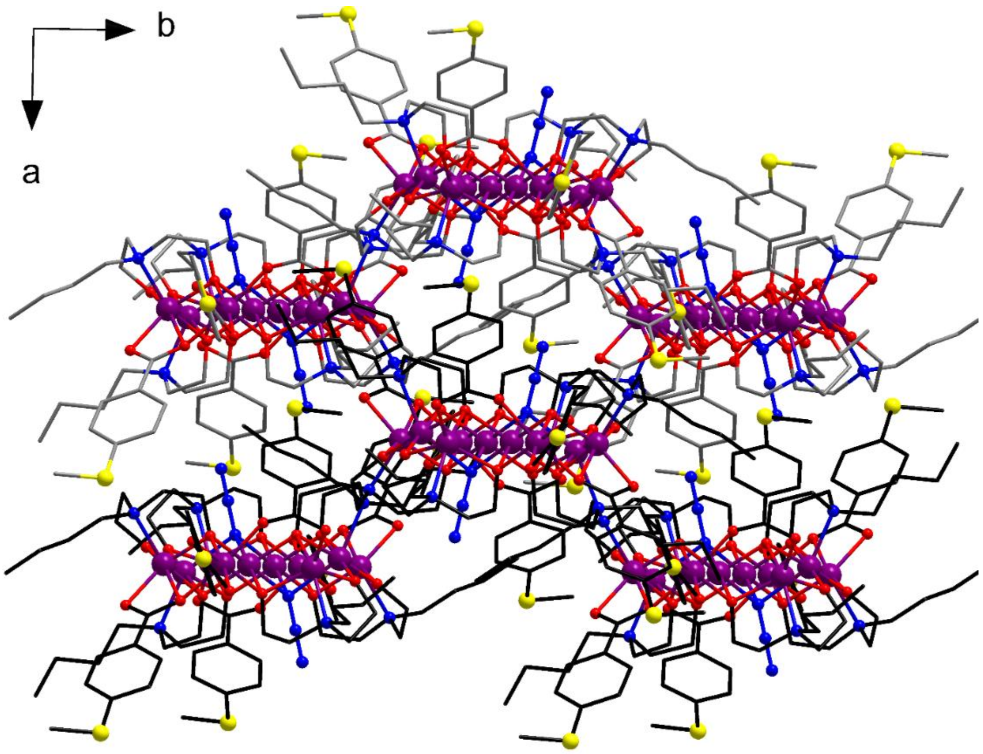

Figure S12. Crystal packing of **3** along the crystallographic *c* axis. Color code as in **Figure S10**. C atoms of the neighboring zigzag layers are shown in gray and black for better clarity.

A comparison of the structure of compound 1 with those reported in the literature

Overall, {Co_5} coordination clusters comprising of two corner-sharing Co_3 triangles date back to 1987 when Englert and Strahle reported[1] a [$Co_3^{II}Co_2^{III}(OH)_2(NO_2)_2(acac)_8$] complex (acac = acetylacetonate). The latter was obtained from a solution of cobalt(II) acetylacetonate ($Co(acac)_2$) in CH_2Cl_2, which was reacted incompletely with NO to $Co(acac)_2(NO)$. This reaction solution further reacted with wet air to form the product. The obtained metal core structure differs from the one of compound **1** in the oxidation states ({$Co_3^{II}Co_2^{III}$} vs. {$Co^{II}Co_4^{III}$} in **1**) and in the coordination modes of the cobalt ions (all octahedral vs. four octahedral and one tetrahedral in **1**).

In 2006 Ferguson *et al*. described a [$Co^{II}Co^{III}_4(HL)_2(H_2L)_2$] complex with H_5L = 2-[bis(2-hydroxyethyl)amino]-2-(hydroxymethyl)propane-1,3-diol as a ligand.[2] This complex was obtained in a 6 % yield (compared to 42 % for **1**) from the reaction of $CoCl_2 \cdot 6H_2O$, H_5L and sodium methoxide in MeOH in a 1:1:4 ratio under aerobic conditions. Here the metal core is very similar to the one in compound **1**. Interestingly, $CoCl_2 \cdot 6H_2O$ under the reaction conditions used in our work led to the formation of compound **2**.

In 2015 Funes *et al*. reported a carboxylate aminoalcohol-based {Co_5} complex isolated as [$Co^{II}Co^{III}_4(piv)_4(teaH)_2(bicH)_2(OH)_2$]$\cdot 4H_2O \cdot CH_3CN$ (Hpiv = pivalic acid, H_3tea = triethanolamine and H_3bic = bicine, 2-(bis-(2-hydroxyethyl)amino)acetic acid).[3] It was obtained from the reaction of the precursor [$Co_2(piv)_4(Hpiv)_4(H_2O)$] and triethanolamine in an 1:4 molar ratio in MeCN under ambient conditions. This mixed-valent {$Co^{II}Co^{III}_4$} complex was isolated as red needle-shaped crystals in 50 % yield after 3–4 weeks. Its metal-core with the distorted tetrahedral Co(II) center and the four octahedral coordinated Co(III) ions is similar to that of **1**. Contrary to **1**, Funes *et al*. used a precursor complex as metal source instead of a simple metal salt and one of the ligands formed *in situ*.

In 2017 Li *et al*. published[4] a mixed-valent {$Co^{II}Co^{III}_4$} complex, [$Co^{II}Co^{III}_4(mda)_4(N_3)_4(piv)_2$] ($H_2$mda = *N*-methyldiethanolamine), which is similar to the one of Funes *et al*. This complex was obtained from the reaction of cobalt(II) perchlorate-hexahydrate ($Co(ClO_4)_2 \cdot 6H_2O$), [$Co_2(piv)_4(Hpiv)_4(H_2O)$], NaN_3 and H_2mda in a 2.0:1.0:1.7:6.9 molar ratio in MeCN under ambient conditions. The product was isolated as dark red crystals in a yield of 28 % after 4 days. (Notably, both the complexes of Funes *et al*. and Li *et al*. are red colored, whereas the [$Co^{II}Co^{III}_4(N_3)_4$(*N*-*n*-bda)$_4$(bza·SMe)$_2$] complex (**1**) is dark green.) Contrary to Li *et al*. who

employed the $[Co_2(piv)_4(Hpiv)_4(H_2O)]$ complex as an additional metal source for two carboxylate ligands in the $[Co^{II}Co^{III}_4(mda)_4(N_3)_4(piv)_2]$ complex, the carboxylate ligands of **1** come from the carboxylic acid introduced in the reaction solution, and no metal complex-precursor was used.

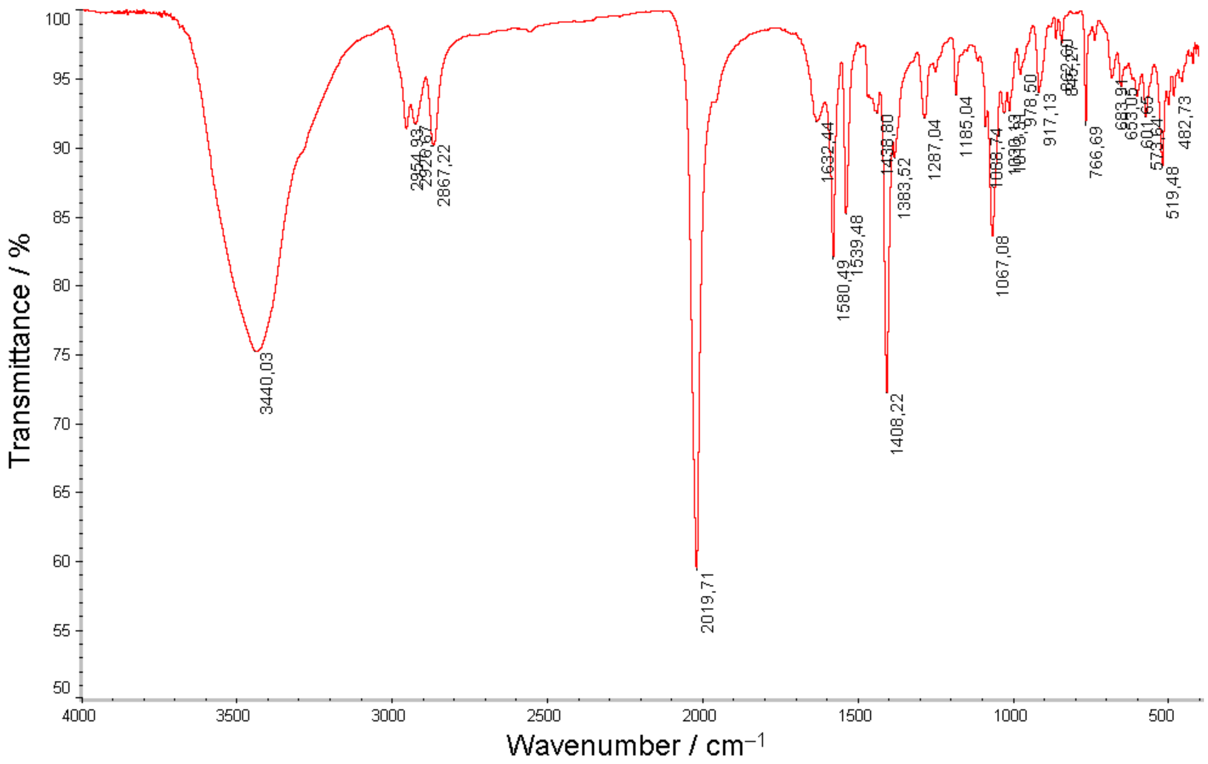

Figure S13. IR spectrum of compound **1** measured in the 450–4000 cm^{-1} region, using a KBr pellet.

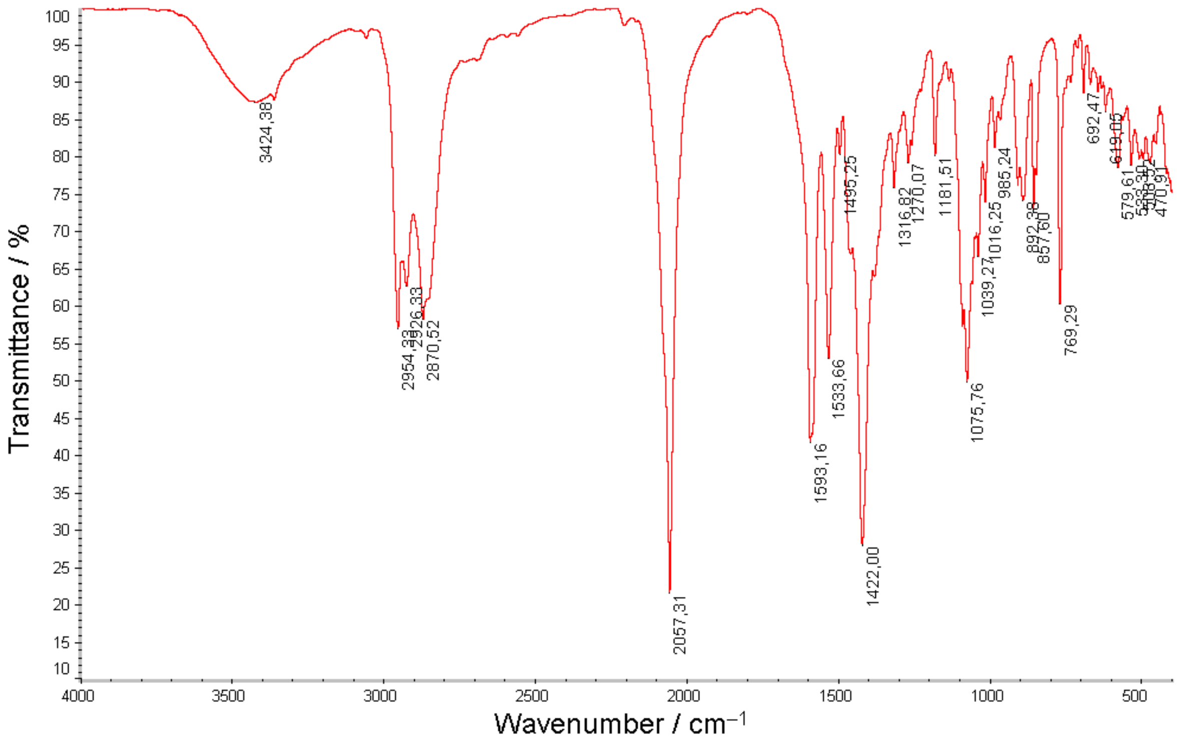

Figure S14. IR spectrum of compound **1A** measured in the 450–4000 cm^{-1} region, using a KBr pellet.

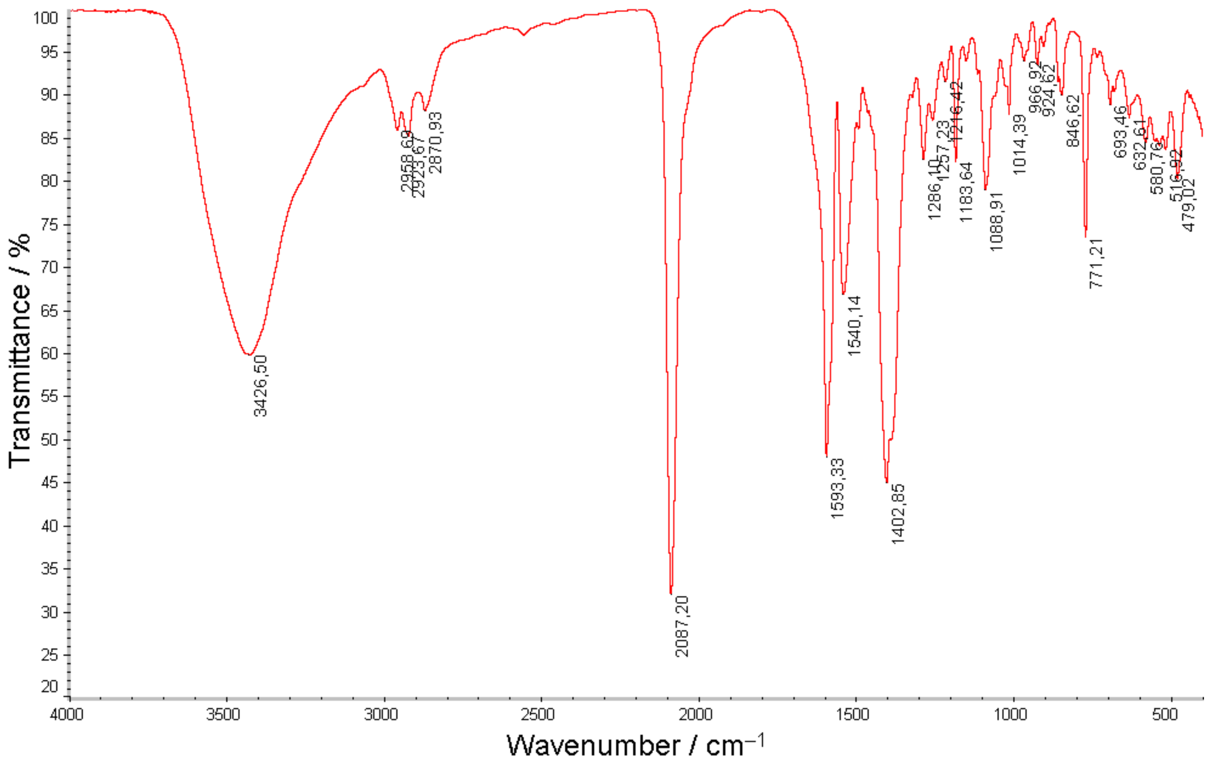

Figure S15. IR spectrum of compound **2** measured in the 450–4000 cm^{-1} region, using a KBr pellet.

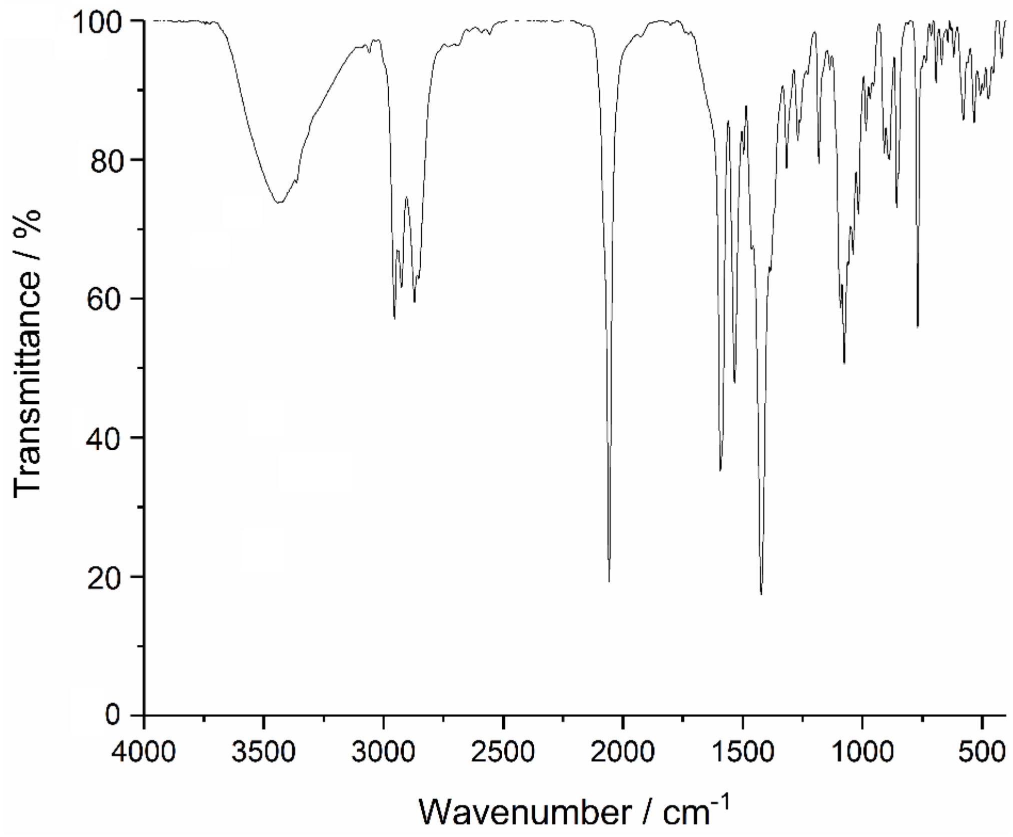

Figure S16. IR spectrum of compound **3** measured in the 450–4000 cm^{-1} region, using a KBr pellet.

Discussion of IR spectra of 1 and 1-A

The IR spectrum of freshly prepared dark-green compound **1** shows typical asymmetric and symmetric C–H stretching vibration bands in the 2955–2867 cm^{-1} range and their asymmetric and symmetric deformation vibrations in the region of 1439–1384 cm^{-1}, which overlap with the sym. stretching vibration band of the COO^- group at 1408 cm^{-1}. The very strong vibration band at 2020 cm^{-1} belongs to the asymmetric stretching vibration of $–N{=}N^+{=}N^-$ from the terminal-coordinated azide groups of the complex. Their symmetric stretching vibrations are observed at 1287 cm^{-1} (weak). In the 1632–1539 cm^{-1} range the C=C stretching vibration of the aromatic ring of bza·SMe^- and the asymmetric stretching vibration of its COO^- group are present. The characteristic C–C–O stretching vibration of the primary alcohol-groups of the ethoxide groups of *N*-*n*-bda^{2-} occurs at 1067 cm^{-1}.

In comparison, the IR spectrum of freshly prepared dark-violet compound **1-A** shows the typical asymmetric and symmetric C–H stretching vibration bands in nearly the same range 2954–2870 cm^{-1}. The intense vibration band of the asymmetric stretching vibration of $–N{=}N^+{=}N^-$ is however shifted to 2057 cm^{-1} ($\Delta\tilde{\nu}$ = 37 cm^{-1}), which indicates that after the thermal treatment not all azide ligands are de-coordinated and that their terminal coordination mode at the Co(III) ions is changed to a bridging mode. This is in line with the observations made for the N_3^- coordination in the reported cobalt complexes (terminal azides: 2010 cm^{-1}, bridging azides: 2054 cm^{-1}, $\Delta\tilde{\nu}$ = 44 cm^{-1} // terminal azides: 2060–2050 cm^{-1}, bridging azides: 2095–2090 cm^{-1}, $\Delta\tilde{\nu}$ = 30–45 cm^{-1}).[5,6]

This assumption is underlined by the shift and the split of the symmetric stretching vibration of the N_3^- groups from 1287 cm^{-1} to 1317 cm^{-1} and 1270 cm^{-1}. The asymmetric and symmetric stretching vibration bands of the COO^- group are slightly shifted from 1580 cm^{-1} to 1593 cm^{-1} and from 1408 cm^{-1} to 1422 cm^{-1}. The characteristic C–C–O stretching vibration of the primary alcohol-groups of the ethoxide rests of the *N*-*n*-bda^{2-} are detected at 1076 cm^{-1} (before: 1067 cm^{-1}). The similarity of the IR spectra of **1** and **1-A** indicates that **1-A** is a polynuclear cobalt complex formed during the thermal treatment of **1**.

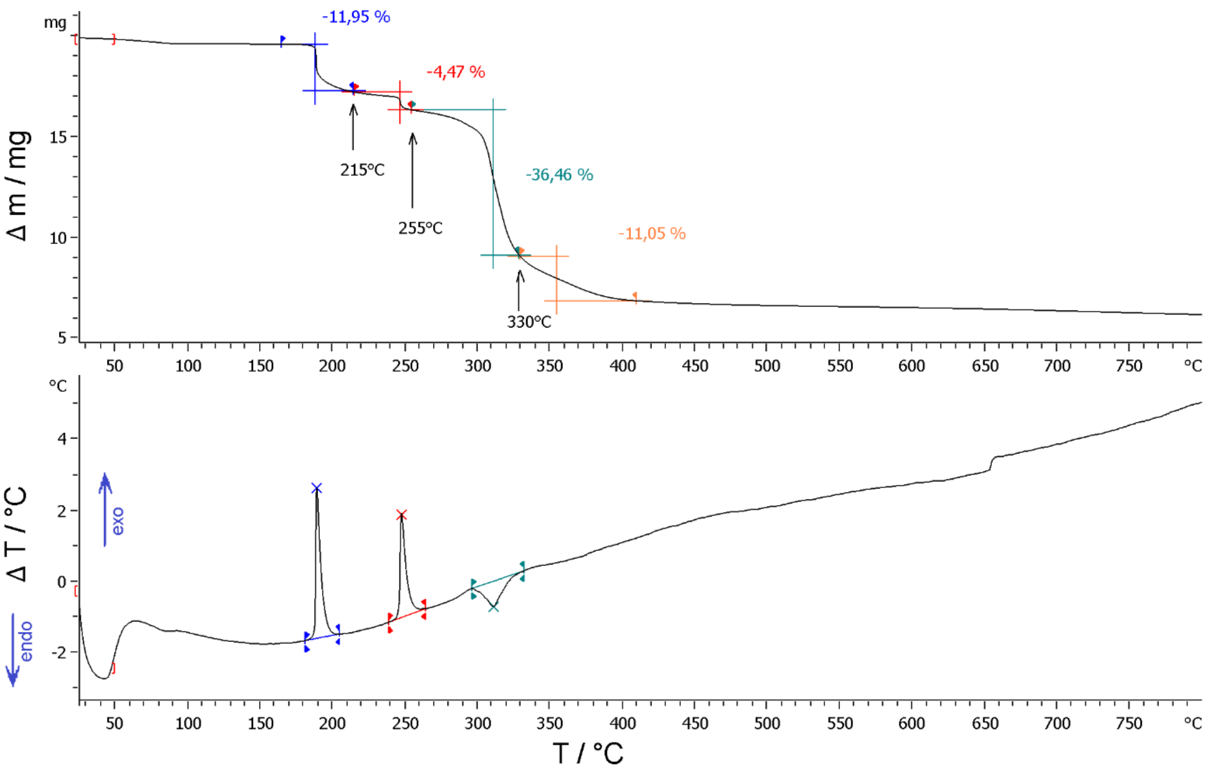

Figure S17. TG-DTA thermogram of compound **1** under nitrogen atmosphere.

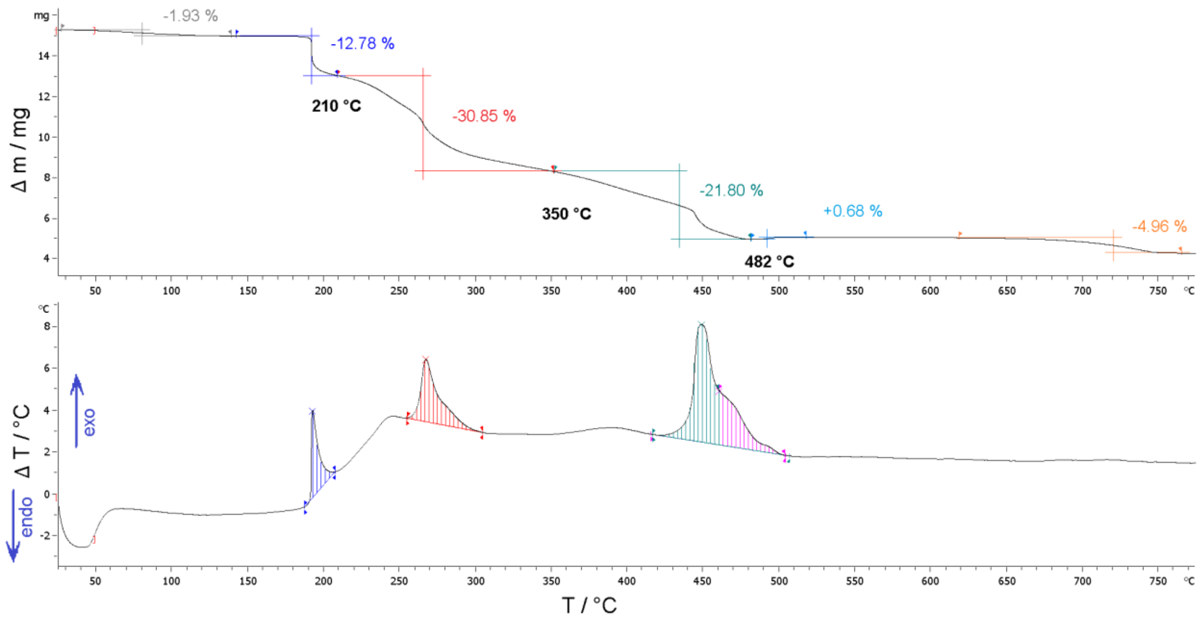

Figure S18. TG-DTA thermogram of compound **1** in dry air.

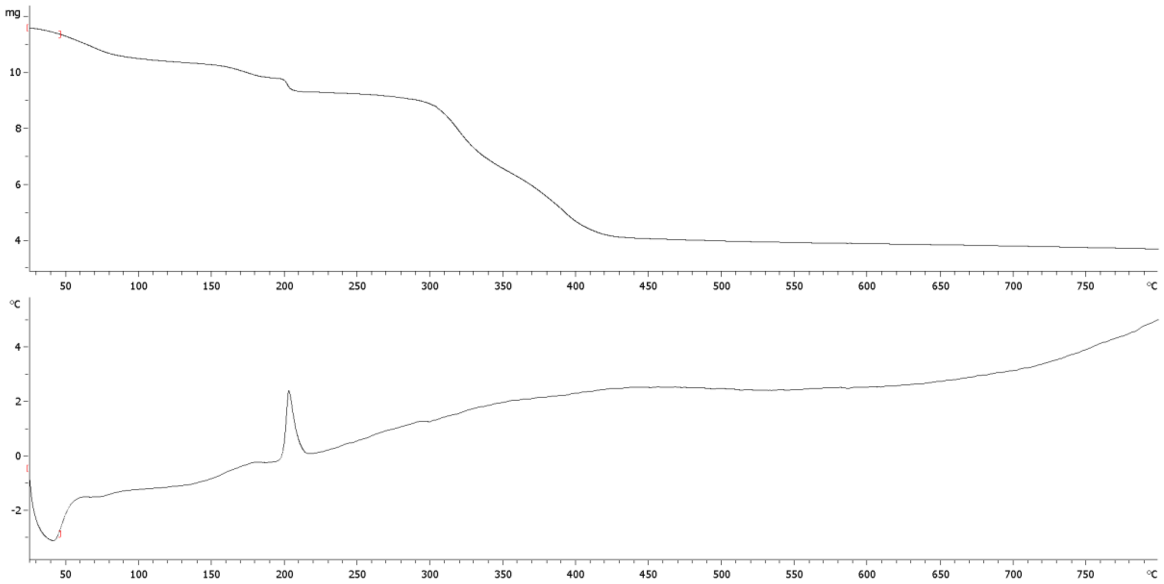

Figure S19. TG-DTA thermogram of compound **2** under nitrogen atmosphere.

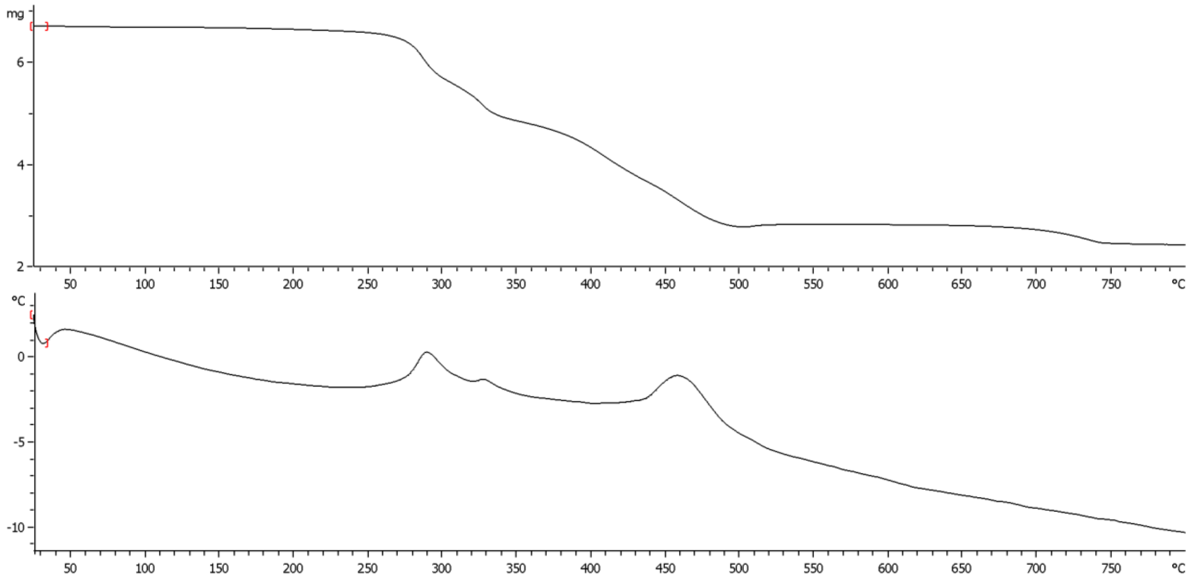

Figure S20. TG-DTA thermogram of compound **3** in dry air.

According to thermogravimetric analyses (TGA), complexes **1** and **2** exhibit a similar thermal stability against degradation under N_2 and in dry air. The TGA thermogram of compound **1** points out three differently sized steps. The first step in the range of 170–220 °C is exothermic and corresponds presumably to an 11.95 % weight loss of two $C_2H_3O^-$ groups (two ethynol and H_2) from the *N*-*n*-bda^{2-} ligands and two azide groups ($\Delta m_{calcd.}$ = 11.86 %). The second exothermic step in the range of 220–255 °C shows a 4.47 % weight loss. The third step is endothermic and the largest one with a weight loss of *ca.* 48 % in the range of 255–440 °C, where the complex fully decomposes.

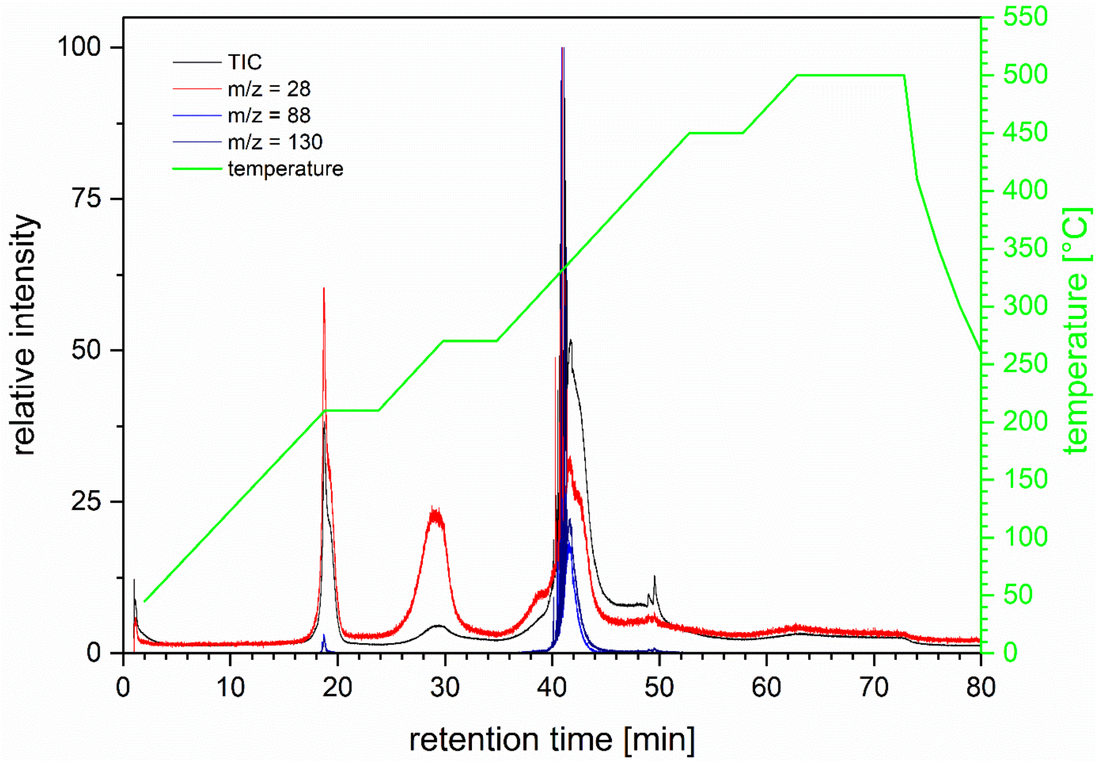

Figure S21. Thermally dependent EI-MS spectrum of compound **1**.

Heating up **1** while performing electron ionization mass spectrometry (EI-MS) of the gas flow shows that obtained weight loss in the TGA curve between 190 and 210 °C (in dry air) does, as expected, belong not only to the loss of nitrogen (*m/z* = 28). Additional mass peaks are observed at *m/z* = 88 (morpholinium) and *m/z* = 130 (2-(butyl(methyl)amino)ethan-1-ol), which emphasizes the assumption that not only azide ligands decompose at the first step in the TGA curve. Another argument for this is, that a nitrogen loss (*m/z* = 28) is detected *at ca.* 240 °C and at *ca.* 330 °C, which partially comes from the remaining azide ligands.

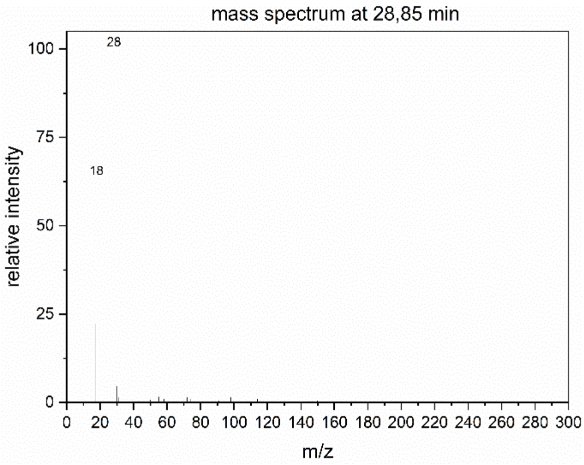

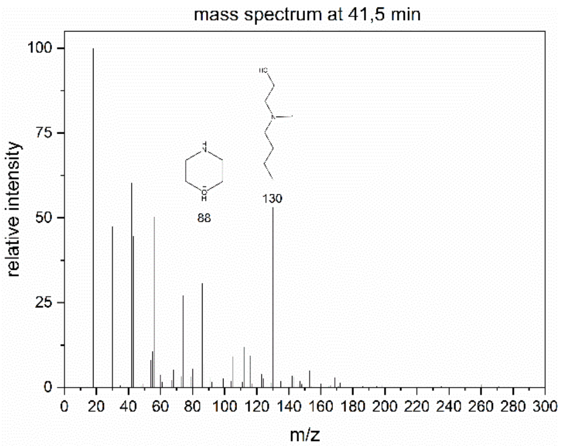

Table S2. ESI-MS data of compounds **1** and **2** obtained in MeOH in the positive ion mode.

Measured [Da]	Calculated [Da]	Sum formula	Fragment ion
		Compound **1**	
1456.2314	1456.2292	$C_{48}Co_5H_{82}N_{16}NaO_{12}S_2^+$	$[NaCo^{II}Co^{III}_4(N_3)_4(N\text{-}n\text{-bda})_4(bza{\cdot}SMe)_2]^+$
381.2976	381.2992	$C_{48}Co_5H_{82}N_{16}Na_4O_{12}S_2^{4+}$	$[Na_4Co^{II}Co^{III}_4(N_3)_4(N\text{-}n\text{-bda})_4(bza{\cdot}SMe)_2]^{4+}$
647.5589	647.5735	$C_{40}Co_5H_{70}N_{15}O_{11}S_2^{2+}$	$[Co^{II}_4Co^{III}(N_3)_4(N\text{-}n\text{-bda})_3(bza{\cdot}SMe)_2(H_2O)(H^+)_3]^{2+}$
1391.2323	1391.2302	$C_{48}Co_5H_{82}N_{13}O_{12}S_2^+$	$[Co^{II}Co^{III}_4(N_3)_3(N\text{-}n\text{-bda})_4(bza{\cdot}SMe)_2]^+$
1349.2225	1349.2210	$C_{48}Co_5H_{82}N_{10}O_{12}S_2^+$	$[Co^{II}_2Co^{III}_3(N_3)_2(N\text{-}n\text{-bda})_4(bza{\cdot}SMe)_2]^+$
1307.2125	1307.2117	$C_{48}Co_5H_{82}N_7O_{12}S_2^+$	$[Co^{II}_3Co^{III}_2(N_3)(N\text{-}n\text{-bda})_4(bza{\cdot}SMe)_2]^+$
1265.2025	1265.2025	$C_{48}Co_5H_{82}N_4O_{12}S_2^+$	$[Co^{II}_4Co^{III}(N\text{-}n\text{-bda})_4(bza{\cdot}SMe)_2]^+$
		Compound **2**	
1675.0194	1674.9708	$C_{56}Co_6H_{69}N_{14}O_{14}S_5^+$	$[Co^{II}_4Co^{III}_2(N_3)_4(N\text{-}n\text{-bda})_2(bza{\cdot}SMe)_5]^+$

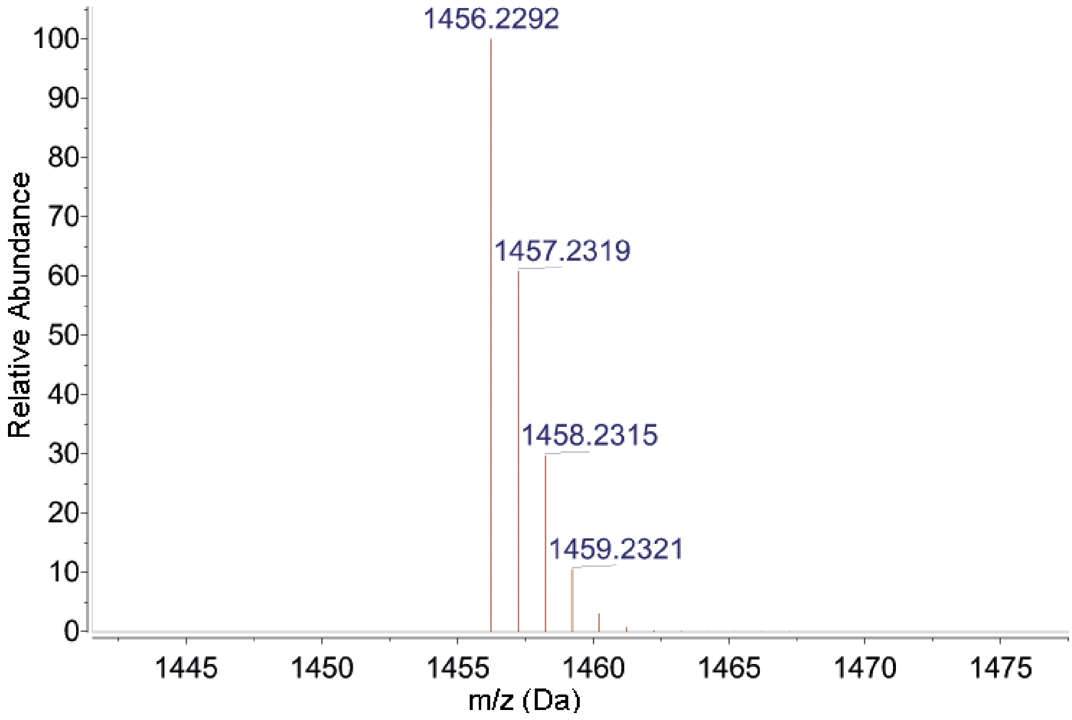

Figure S22. The simulated molecular ion peak of $[NaCo^{II}Co^{III}_4(N_3)_4(N\text{-}n\text{-bda})_4(bza{\cdot}SMe)_2]^+$ (= $C_{48}Co_5H_{82}N_{16}NaO_{12}S_2^+$) in compound **1**.

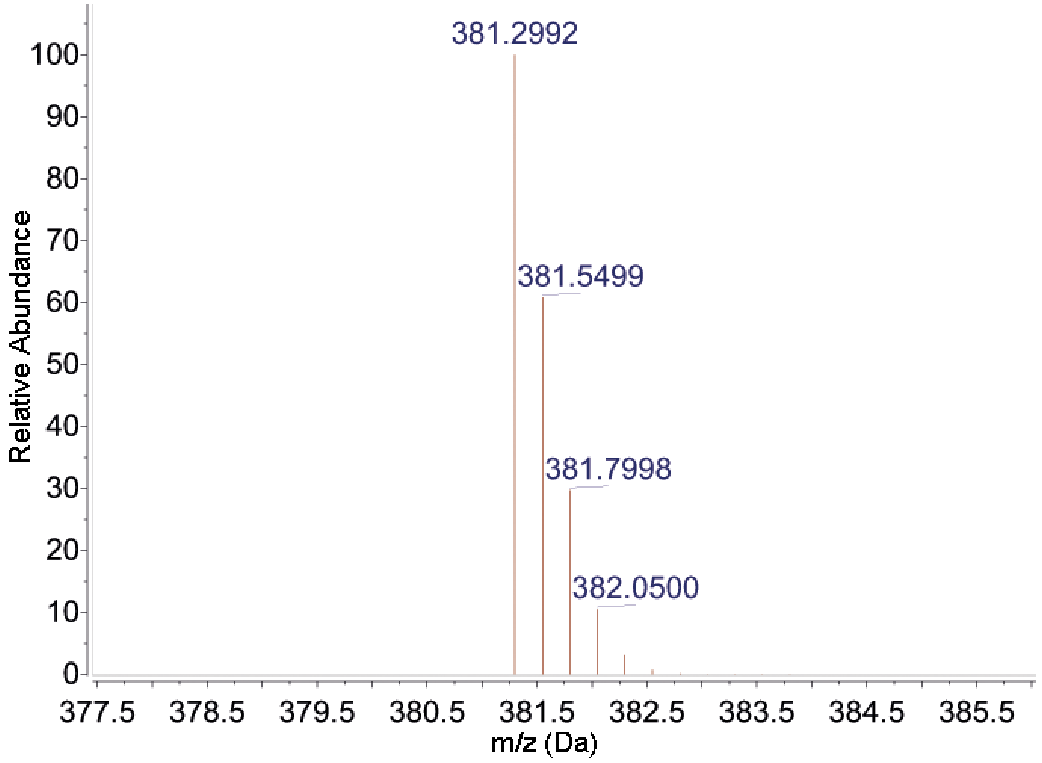

Figure S23. The simulated molecular ion peak of $[Na_4Co^{II}Co^{III}_4(N_3)_4(N\text{-}n\text{-bda})_4(\text{bza}\cdot\text{SMe})_2]^{4+}$ (= $C_{48}Co_5H_{82}N_{16}Na_4O_{12}S_2^{4+}$) in compound **1**.

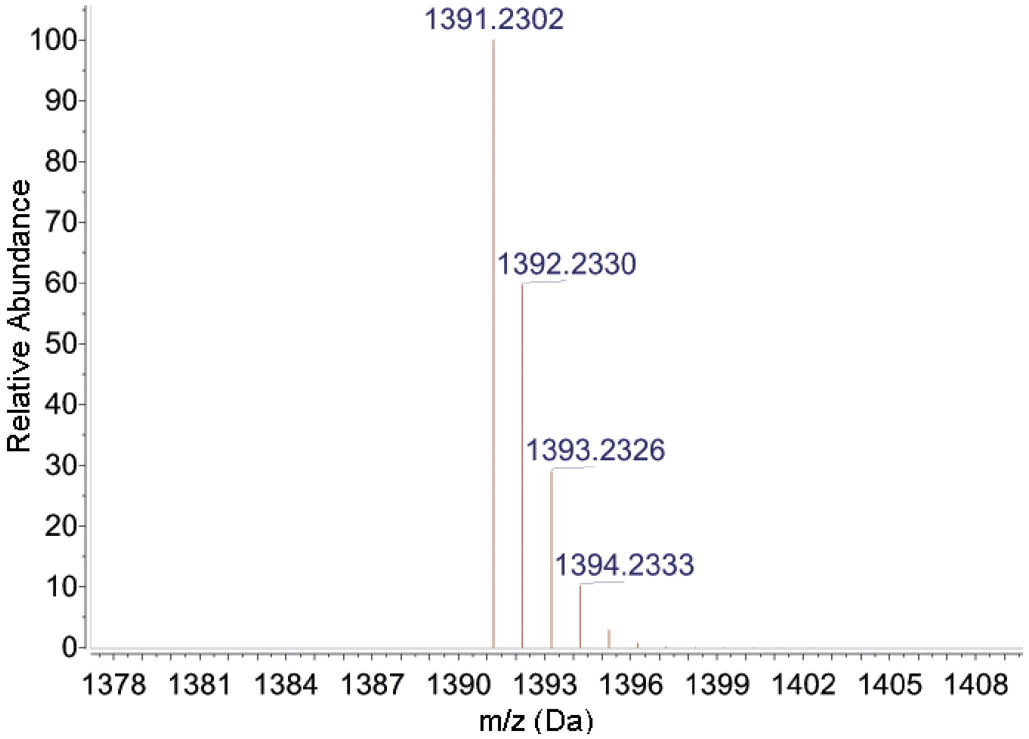

Figure S24. The simulated molecular ion peak of $[Co^{II}Co^{III}_4(N_3)_3(N\text{-}n\text{-bda})_4(\text{bza}\cdot\text{SMe})_2]^{+}$ (= $C_{48}Co_5H_{82}N_{13}O_{12}S_2^{+}$) in compound **1**.

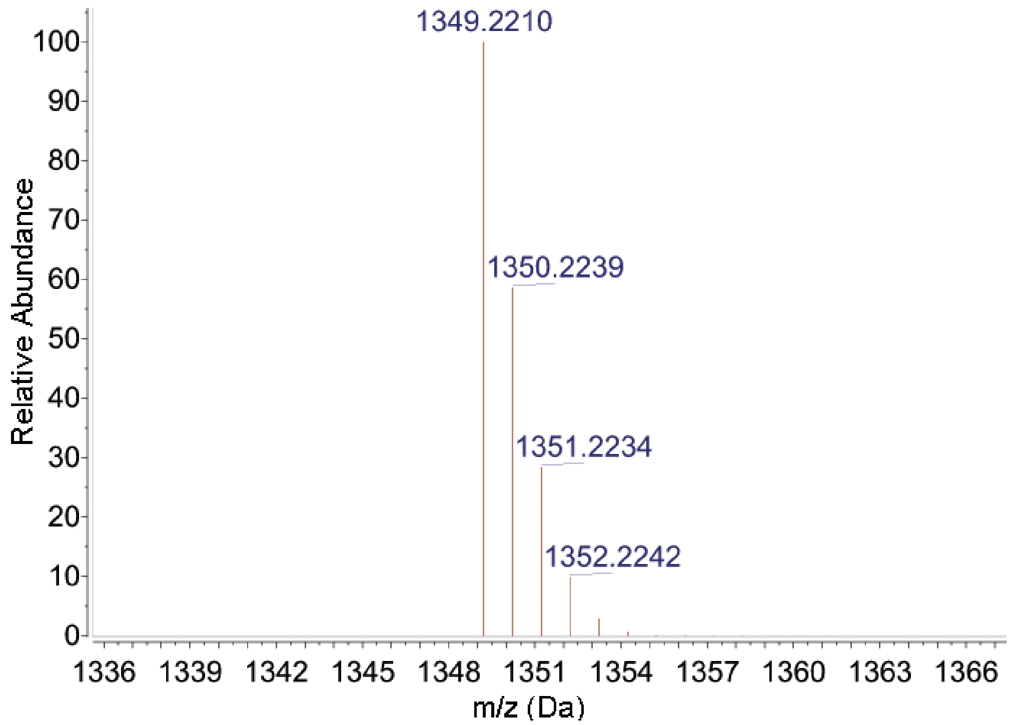

Figure S25. The simulated molecular ion peak of $[Co^{II}_2Co^{III}_3(N_3)_2(N\text{-}n\text{-bda})_4(bza{\cdot}SMe)_2]^+$ (= $C_{48}Co_5H_{82}N_{10}O_{12}S_2^+$) in compound **1**.

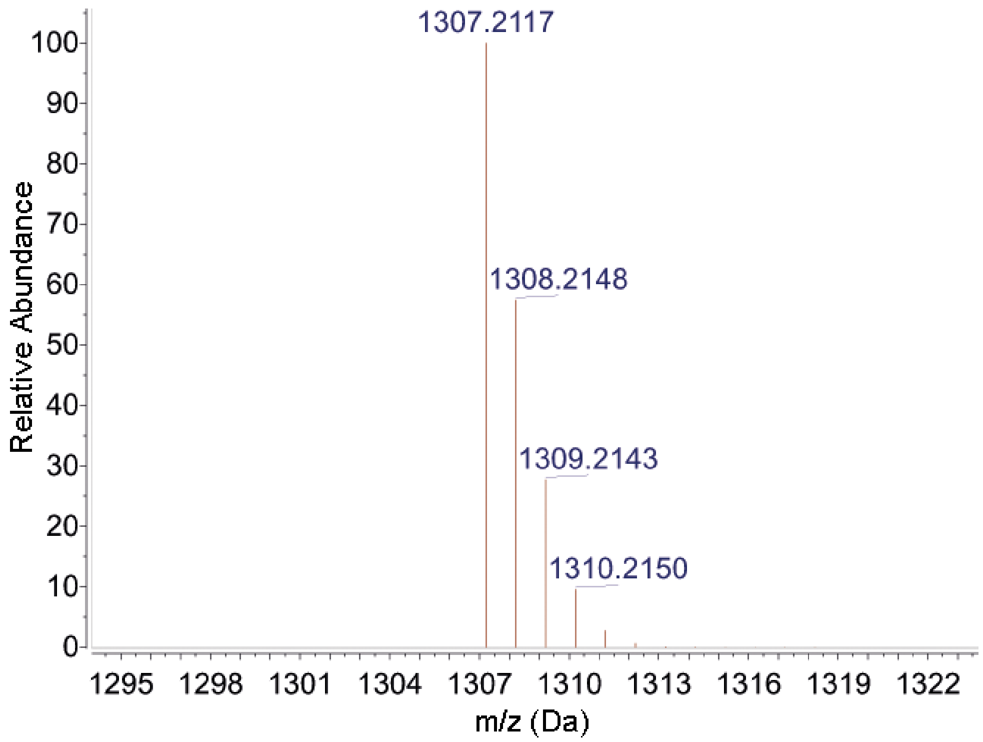

Figure S26. The simulated molecular ion peak of $[Co^{II}_3Co^{III}_2(N_3)(N\text{-}n\text{-bda})_4(bza{\cdot}SMe)_2]^+$ (= $C_{48}Co_5H_{82}N_7O_{12}S_2^+$) in compound **1**.

References

1) U. Englert and J. Strahle, *Z. Naturforsch*. **1987**, *42b*, 959–966.

2) A. Ferguson, A. Parkin and M. Murrie, *Dalton Trans*. **2006**, 3627–3628.

3) A. V. Funes, L. Carrella, L. Sorace, E. Rentschler and P. Alborés, *Dalton Trans.* **2015**, *44*, 2390–2400.

4) Y.-W. Li, L.-Y. Guo, L. Feng, Z. Jagličić, S.-Y. Zeng and D. Sun, *CrystEngComm* **2017**, *19*, 5897–5906.

5) S. Mandal, S. Mondal, C. Rajnák, J. Titiš, R. Boča and S. Mohanta, *Dalton Trans*. **2017**, *46*, 13135–13144.

6) U. Ray, B. Chand, G. Mostafa, J. Cheng, T.-H. Lu and C. Sinha, *Polyhedron* **2003**, *22*, 2587–2594.
