# Supplementary material for: Conductive Self-Assembled Monolayers of Paramagnetic {CoIICo4III} and {Co4IICo2III} Coordination Clusters on Gold Surfaces
Source: Front Chem. 2019 Nov 5;7:681. doi: 10.3389/fchem.2019.00681 (PMC6848059; doi:10.3389/fchem.2019.00681)

checkCIF/PLATON report

Structure factors have been supplied for datablock(s) S1948b

THIS REPORT IS FOR GUIDANCE ONLY. IF USED AS PART OF A REVIEW PROCEDURE FOR PUBLICATION, IT SHOULD NOT REPLACE THE EXPERTISE OF AN EXPERIENCED CRYSTALLOGRAPHIC REFEREE.

No syntax errors found. CIF dictionary Interpreting this report

Datablock: S1948b

Bond precision:	C-C = 0.0121 A		Wavelength=0.71073
Cell:	a=16.2281(2)	b=31.8717(8)	c=34.3157(8)
	alpha=90	beta=90	gamma=90
Temperature:	120 K		

	Calculated	Reported
Volume	17748.7(6)	17748.7(7)
Space group	P b c a	P b c a
Hall group	-P 2ac 2ab	-P 2ac 2ab
Moiety formula	4(C60 H77 Co6 N14 O18 S5), 9(C O), 4(Cl)	?
Sum formula	C249 H308 Cl4 Co24 N56 O81 S20	C62.25 H94 Cl Co6 N14 O20.25 S5
Mr	7578.85	1911.84
Dx,g cm-3	1.418	1.431
Z	2	8
Mu (mm-1)	1.311	1.312
F000	7756.0	7892.0
F000’	7781.52	
h,k,lmax	19,37,40	19,37,40
Nref	15672	15629
Tmin,Tmax	0.721,0.932	0.604,0.875
Tmin’	0.699	

Correction method= # Reported T Limits: Tmin=0.604 Tmax=0.875
AbsCorr = ANALYTICAL

Data completeness= 0.997 Theta(max)= 25.027

R(reflections)= 0.0786(9158) wR2(reflections)= 0.2116(15629)

S = 1.043 Npar= 1013

The following ALERTS were generated. Each ALERT has the format
test-name_ALERT_alert-type_alert-level.
Click on the hyperlinks for more details of the test.

Alert level B

PLAT315_ALERT_2_B Singly Bonded Carbon Detected (H-atoms Missing). C22M Check

Author Response: Hydrogen atoms of disordered -CH3 groups were not located.

PLAT315_ALERT_2_B Singly Bonded Carbon Detected (H-atoms Missing). C21M Check

Author Response: Hydrogen atoms of disordered -CH3 groups were not located.

PLAT315_ALERT_2_B Singly Bonded Carbon Detected (H-atoms Missing). C1M Check

Author Response: Hydrogen atoms of disordered -CH3 groups were not located.

PLAT315_ALERT_2_B Singly Bonded Carbon Detected (H-atoms Missing). C2M Check

Author Response: Hydrogen atoms of disordered -CH3 groups were not located.

PLAT315_ALERT_2_B Singly Bonded Carbon Detected (H-atoms Missing). C3M Check

Author Response: Hydrogen atoms of disordered -CH3 groups were not located.

PLAT315_ALERT_2_B Singly Bonded Carbon Detected (H-atoms Missing). C4M Check

Author Response: Hydrogen atoms of disordered -CH3 groups were not located.

PLAT430_ALERT_2_B Short Inter D...A Contact O1M ..O31M . 2.66 Ang.
x,y,z = 1_555 Check

Author Response: The relatively short O...O contacts arise due to hydrogen bonding.

PLAT430_ALERT_2_B Short Inter D...A Contact O2M ..O3M . 2.69 Ang.
-x,2-y,1-z = 5_576 Check

Author Response: The relatively short O...O contacts arise due to hydrogen bonding.

PLAT430_ALERT_2_B Short Inter D...A Contact O2M ..O21M . 2.71 Ang.
x,y,z = 1_555 Check

Author Response: The relatively short O...O contacts arise due to hydrogen bonding.

PLAT430_ALERT_2_B Short Inter D...A Contact O4M ..O11M . 2.68 Ang.
x,y,z = 1_555 Check

Author Response: The relatively short O...O contacts arise due to hydrogen bonding.

```
PLAT910_ALERT_3_B Missing # of FCF Reflection(s) Below Theta(Min).        43 Note
```

Author Response: Missing reflections are in diffractometer restricted areas like beamstop. Nevertheless, the total amount of collected reflections well exceeds that required for statistically relevant refinement to assure true results.

Alert level C

```
RINTA01_ALERT_3_C  The value of Rint is greater than 0.12
            Rint given   0.150
PLAT020_ALERT_3_C The Value of Rint is Greater Than 0.12 .........      0.150 Report
PLAT041_ALERT_1_C Calc. and Reported SumFormula    Strings  Differ     Please Check
PLAT043_ALERT_1_C Calculated and Reported Mol. Weight Differ by ..      68.51 Check
PLAT068_ALERT_1_C Reported F000 Differs from Calcd (or Missing)...     Please Check
PLAT220_ALERT_2_C Non-Solvent  Resd 1  C   Ueq(max)/Ueq(min) Range        4.5 Ratio
PLAT220_ALERT_2_C Non-Solvent  Resd 1  O   Ueq(max)/Ueq(min) Range        3.4 Ratio
PLAT222_ALERT_3_C Non-Solv.  Resd 1  H   Uiso(max)/Uiso(min) Range        5.5 Ratio
PLAT234_ALERT_4_C Large Hirshfeld Difference O11M     --C11M     .       0.24 Ang.
PLAT234_ALERT_4_C Large Hirshfeld Difference O1L5     --C1L5     .       0.16 Ang.
PLAT309_ALERT_2_C Single Bonded Oxygen (C-O > 1.3 Ang) ..........         O1M Check
PLAT309_ALERT_2_C Single Bonded Oxygen (C-O > 1.3 Ang) ..........         O2M Check
PLAT309_ALERT_2_C Single Bonded Oxygen (C-O > 1.3 Ang) ..........         O3M Check
PLAT309_ALERT_2_C Single Bonded Oxygen (C-O > 1.3 Ang) ..........         O4M Check
PLAT341_ALERT_3_C Low Bond Precision on  C-C Bonds ..............    0.01209 Ang.
PLAT601_ALERT_2_C Structure Contains Solvent Accessible VOIDS of .         56 Ang**3
PLAT906_ALERT_3_C Large K Value in the Analysis of Variance ......      7.666 Check
PLAT906_ALERT_3_C Large K Value in the Analysis of Variance ......      2.013 Check
PLAT913_ALERT_3_C Missing # of Very Strong Reflections in FCF ....          4 Note
PLAT975_ALERT_2_C Check Calcd Resid. Dens.  0.71A   From C2M             1.09 eA-3
PLAT975_ALERT_2_C Check Calcd Resid. Dens.  1.07A   From O1M             0.81 eA-3
PLAT975_ALERT_2_C Check Calcd Resid. Dens.  1.03A   From C1M             0.71 eA-3
PLAT975_ALERT_2_C Check Calcd Resid. Dens.  0.59A   From C3M             0.60 eA-3
PLAT978_ALERT_2_C Number C-C Bonds with Positive Residual Density.          0 Info
```

Alert level G

```
FORMU01_ALERT_2_G  There is a discrepancy between the atom counts in the
            _chemical_formula_sum and the formula from the _atom_site* data.
            Atom count from _chemical_formula_sum:C62.25 H94 Cl1 Co6 N14 O20.25 S5
            Atom count from the _atom_site data:  C62.25 H77 Cl1 Co6 N14 O20.25 S5
CELLZ01_ALERT_1_G Difference between formula and atom_site contents detected.
CELLZ01_ALERT_1_G WARNING: H atoms missing from atom site list. Is this intentional?
           From the CIF: _cell_formula_units_Z    8
           From the CIF: _chemical_formula_sum  C62.25 H94 Cl Co6 N14 O20.25 S5
           TEST: Compare cell contents of formula and atom_site data
```

atom	Z*formula	cif sites	diff
C	498.00	498.00	0.00
H	752.00	616.00	136.00
Cl	8.00	8.00	0.00
Co	48.00	48.00	0.00
N	112.00	112.00	0.00
O	162.00	162.00	0.00
S	40.00	40.00	0.00

```
PLAT003_ALERT_2_G Number of Uiso or Uij Restrained non-H Atoms ...          2 Report
PLAT045_ALERT_1_G Calculated and Reported Z Differ by a Factor ...       0.25 Check
PLAT083_ALERT_2_G SHELXL Second Parameter in WGHT  Unusually Large      66.33 Why ?
PLAT171_ALERT_4_G The CIF-Embedded .res File Contains EADP Records          1 Report
PLAT186_ALERT_4_G The CIF-Embedded .res File Contains ISOR Records          1 Report
PLAT300_ALERT_4_G Atom Site Occupancy of O1M       Constrained at        0.75 Check
PLAT300_ALERT_4_G Atom Site Occupancy of C1M       Constrained at        0.75 Check
PLAT300_ALERT_4_G Atom Site Occupancy of O2M       Constrained at         0.5 Check
PLAT300_ALERT_4_G Atom Site Occupancy of C2M       Constrained at         0.5 Check
PLAT300_ALERT_4_G Atom Site Occupancy of O3M       Constrained at         0.5 Check
PLAT300_ALERT_4_G Atom Site Occupancy of C3M       Constrained at         0.5 Check
PLAT300_ALERT_4_G Atom Site Occupancy of O4M       Constrained at         0.5 Check
PLAT300_ALERT_4_G Atom Site Occupancy of C4M       Constrained at         0.5 Check
PLAT301_ALERT_3_G Main Residue  Disorder .............(Resd  1  )         1% Note
PLAT302_ALERT_4_G Anion/Solvent/Minor-Residue Disorder (Resd  2  )       100% Note
PLAT302_ALERT_4_G Anion/Solvent/Minor-Residue Disorder (Resd  3  )       100% Note
PLAT302_ALERT_4_G Anion/Solvent/Minor-Residue Disorder (Resd  4  )       100% Note
PLAT302_ALERT_4_G Anion/Solvent/Minor-Residue Disorder (Resd  5  )       100% Note
PLAT304_ALERT_4_G Non-Integer Number of Atoms in ...... Resd  2          1.50 Check
PLAT343_ALERT_2_G Unusual sp?      Angle Range in Main Residue for       C7L3 Check
PLAT431_ALERT_2_G Short Inter HL..A Contact  Cl1      ..O1M      .       3.00 Ang.
                                                      x,y,z  =      1_555 Check
PLAT431_ALERT_2_G Short Inter HL..A Contact  Cl1      ..O41M     .       3.08 Ang.
                                                      x,y,z  =      1_555 Check
PLAT431_ALERT_2_G Short Inter HL..A Contact  Cl1      ..O3M      .       3.12 Ang.
                                                      x,y,z  =      1_555 Check
PLAT431_ALERT_2_G Short Inter HL..A Contact  Cl1      ..O4M      .       3.12 Ang.
                                                 -x,2-y,1-z  =      5_576 Check
PLAT720_ALERT_4_G Number of Unusual/Non-Standard Labels ..........        133 Note
PLAT790_ALERT_4_G Centre of Gravity not Within Unit Cell: Resd.  #          2 Note
              C O
PLAT790_ALERT_4_G Centre of Gravity not Within Unit Cell: Resd.  #          4 Note
              C O
PLAT790_ALERT_4_G Centre of Gravity not Within Unit Cell: Resd.  #          5 Note
              C O
PLAT793_ALERT_4_G Model has Chirality at N3          (Centro SPGR)          R Verify
PLAT793_ALERT_4_G Model has Chirality at N5          (Centro SPGR)          R Verify
PLAT794_ALERT_5_G Tentative Bond Valency for Co1       (II)      .       1.92 Info
PLAT794_ALERT_5_G Tentative Bond Valency for Co3       (III)     .       3.11 Info
PLAT794_ALERT_5_G Tentative Bond Valency for Co4       (II)      .       1.91 Info
PLAT794_ALERT_5_G Tentative Bond Valency for Co5       (III)     .       3.15 Info
PLAT794_ALERT_5_G Tentative Bond Valency for Co6       (II)      .       2.03 Info
PLAT860_ALERT_3_G Number of Least-Squares Restraints .............         12 Note
PLAT883_ALERT_1_G No Info/Value for _atom_sites_solution_primary .     Please Do !
PLAT909_ALERT_3_G Percentage of I>2sig(I) Data at Theta(Max) Still        33% Note
PLAT933_ALERT_2_G Number of OMIT Records in Embedded .res File ...          1 Note
```

---

  0 **ALERT level A** = Most likely a serious problem - resolve or explain
 11 **ALERT level B** = A potentially serious problem, consider carefully
 24 **ALERT level C** = Check. Ensure it is not caused by an omission or oversight
 42 **ALERT level G** = General information/check it is not something unexpected

  7 ALERT type 1 CIF construction/syntax error, inconsistent or missing data
 31 ALERT type 2 Indicator that the structure model may be wrong or deficient
 11 ALERT type 3 Indicator that the structure quality may be low
 23 ALERT type 4 Improvement, methodology, query or suggestion
  5 ALERT type 5 Informative message, check

It is advisable to attempt to resolve as many as possible of the alerts in all categories. Often the minor alerts point to easily fixed oversights, errors and omissions in your CIF or refinement strategy, so attention to these fine details can be worthwhile. In order to resolve some of the more serious problems it may be necessary to carry out additional measurements or structure refinements. However, the purpose of your study may justify the reported deviations and the more serious of these should normally be commented upon in the discussion or experimental section of a paper or in the "special_details" fields of the CIF. checkCIF was carefully designed to identify outliers and unusual parameters, but every test has its limitations and alerts that are not important in a particular case may appear. Conversely, the absence of alerts does not guarantee there are no aspects of the results needing attention. It is up to the individual to critically assess their own results and, if necessary, seek expert advice.

Publication of your CIF in IUCr journals

A basic structural check has been run on your CIF. These basic checks will be run on all CIFs submitted for publication in IUCr journals (*Acta Crystallographica*, *Journal of Applied Crystallography*, *Journal of Synchrotron Radiation*); however, if you intend to submit to *Acta Crystallographica Section C* or *E* or *IUCrData*, you should make sure that full publication checks are run on the final version of your CIF prior to submission.

Publication of your CIF in other journals

Please refer to the *Notes for Authors* of the relevant journal for any special instructions relating to CIF submission.

PLATON version of 03/05/2019; check.def file version of 29/04/2019

Datablock S1948b - ellipsoid plot

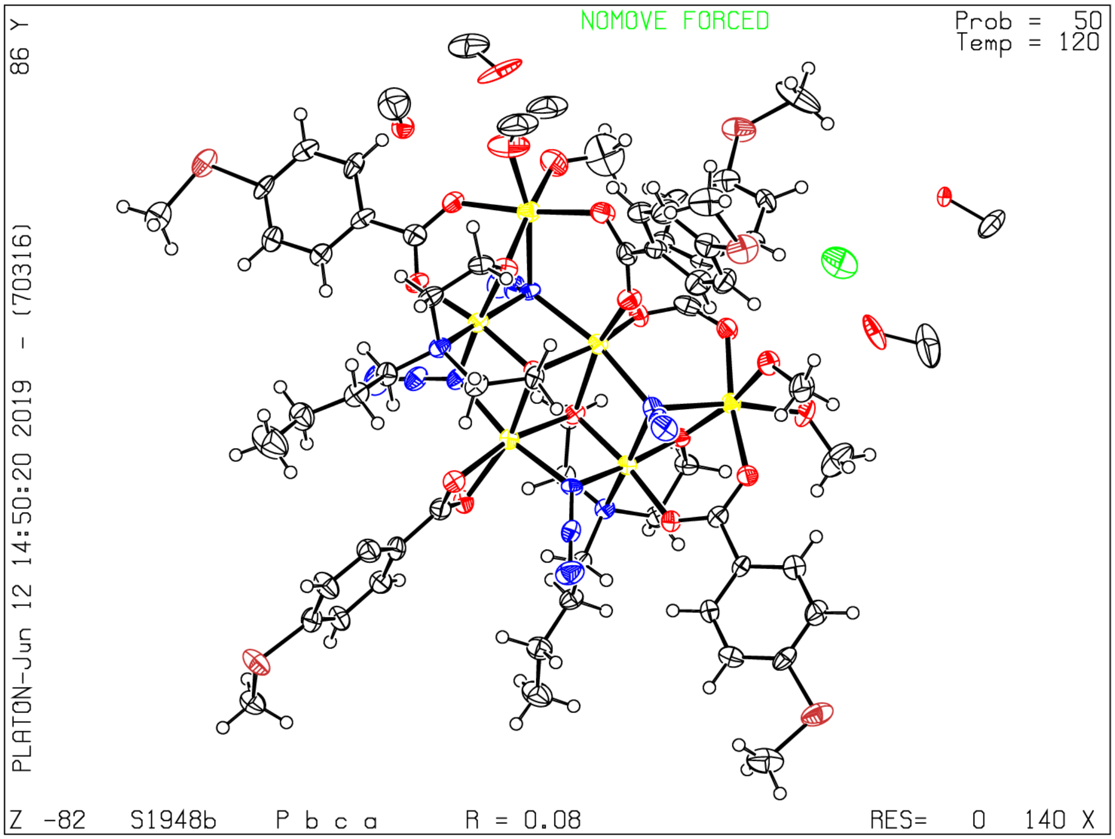

Supplement: Supplementary file 2 [file Data_Sheet_1.ZIP › checkcif_Co6.pdf]
