# Supplementary material for: Conductive Self-Assembled Monolayers of Paramagnetic {CoIICo4III} and {Co4IICo2III} Coordination Clusters on Gold Surfaces
Source: Front Chem. 2019 Nov 5;7:681. doi: 10.3389/fchem.2019.00681 (PMC6848059; doi:10.3389/fchem.2019.00681)

checkCIF/PLATON report

Structure factors have been supplied for datablock(s) Co10

THIS REPORT IS FOR GUIDANCE ONLY. IF USED AS PART OF A REVIEW PROCEDURE FOR PUBLICATION, IT SHOULD NOT REPLACE THE EXPERTISE OF AN EXPERIENCED CRYSTALLOGRAPHIC REFEREE.

No syntax errors found.   CIF dictionary   Interpreting this report

Datablock: Co10

```
Bond precision:  C-C = 0.0074 A          Wavelength=0.71073

Cell:            a=8.9930(6)     b=16.5801(11)      c=37.429(3)
                 alpha=90        beta=90.333(1)      gamma=90
Temperature:     100 K

                Calculated                 Reported
Volume          5580.8(7)                  5580.7(6)
Space group     P 21/n                     P 21/n
Hall group      -P 2yn                     -P 2yn
Moiety formula  C96 H144 Co10 N12 O24 S6   ?
Sum formula     C96 H144 Co10 N12 O24 S6   C96 H144 Co10 N12 O24 S6
Mr              2631.90                    2631.88
Dx,g cm-3       1.566                      1.566
Z               2                          2
Mu (mm-1)       1.631                      1.631
F000            2724.0                     2724.0
F000'           2733.31
h,k,lmax        10,20,45                   10,20,45
Nref            10264                      9754
Tmin,Tmax       0.838,0.878                0.558,0.745
Tmin'           0.676

Correction method= # Reported T Limits: Tmin=0.558 Tmax=0.745
AbsCorr = MULTI-SCAN

Data completeness= 0.950          Theta(max)= 25.398

R(reflections)= 0.0423( 5273)     wR2(reflections)= 0.0828( 9754)

S = 0.776                  Npar= 667
```

The following ALERTS were generated. Each ALERT has the format
test-name_ALERT_alert-type_alert-level.
Click on the hyperlinks for more details of the test.

Alert level B

```
PLAT029_ALERT_3_B _diffrn_measured_fraction_theta_full value Low .      0.951 Why?
```

Author Response: The issue arose from the rather poor diffraction of the crystals at higher angles. Several crystals have been checked at low temperature using a Mo microsource tube and the best of them was selected for the data collections. In any case the quality of the obtained data allows for unambigous structure solution and refinment.

```
PLAT220_ALERT_2_B Non-Solvent  Resd 1  C   Ueq(max)/Ueq(min) Range        6.2 Ratio
```

Author Response: The difference arises from different degree of movement freedom for various carbon atoms in the structure. Thus, for example, the CH3 groups of -S-CH3 grops of 4-(methylthio)benzoates and N-butyldiethanolamine can move much easier than tightly frixed carbons of phenyl rings.

```
PLAT232_ALERT_2_B Hirshfeld Test Diff (M-X)  Co4      --O31S_a   .       14.5 s.u.
```

Author Response: In this case there could not be any wrong atom assignment as the nature of the atom is 100 % clear from the ligand structure and composition. In fact, the failury of the test most likel arises from the relative weakness of the corresponding Co-O bond (2.296(3) angstr.) and thus, a relative freedom for the movement of this O atom.

Alert level C

```
GOODF01_ALERT_2_C  The least squares goodness of fit parameter lies
            outside the range 0.80 <> 2.00
            Goodness of fit given =      0.776
RINTA01_ALERT_3_C  The value of Rint is greater than 0.12
            Rint given   0.125
PLAT213_ALERT_2_C Atom C18             has ADP max/min Ratio .....        3.8 prolat
PLAT220_ALERT_2_C Non-Solvent  Resd 1  O   Ueq(max)/Ueq(min) Range        3.2 Ratio
```

Author Response: The difference arises from different degree of movement freedom for various carbon atoms in the structure. Thus, for example, the CH3 groups of -S-CH3 grops of 4-(methylthio)benzoates and N-butyldiethanolamine can move much easier than tightly frixed carbons of phenyl rings.

```
PLAT222_ALERT_3_C Non-Solv.  Resd 1  H   Uiso(max)/Uiso(min) Range        7.6 Ratio
PLAT230_ALERT_2_C Hirshfeld Test Diff for    N11      --N12      .        6.3 s.u.
PLAT230_ALERT_2_C Hirshfeld Test Diff for    C5N3     --C6N3     .        6.6 s.u.
PLAT230_ALERT_2_C Hirshfeld Test Diff for    C6N3     --C7N3     .        5.2 s.u.
PLAT234_ALERT_4_C Large Hirshfeld Difference C7N1     --C8N1     .       0.18 Ang.
PLAT241_ALERT_2_C High   'MainMol' Ueq as Compared to Neighbors of       O21S Check
PLAT341_ALERT_3_C Low Bond Precision on  C-C Bonds ...............    0.00744 Ang.
PLAT360_ALERT_2_C Short  C(sp3)-C(sp3) Bond  C5N1     - C6N1     .       1.39 Ang.
```

```
PLAT413_ALERT_2_C Short Inter XH3 .. XHn     H18C     ..H27A     .       2.11 Ang.
                                         3/2-x,-1/2+y,1/2-z  =     2_645 Check
PLAT601_ALERT_2_C Structure Contains Solvent Accessible VOIDS of .         62 Ang**3
PLAT911_ALERT_3_C Missing FCF Refl Between Thmin & STh/L=    0.600        493 Report
```

Alert level G

```
PLAT003_ALERT_2_G Number of Uiso or Uij Restrained non-H Atoms ...          6 Report
PLAT020_ALERT_3_G The Value of Rint is Greater Than 0.12 .........      0.125 Report
PLAT177_ALERT_4_G The CIF-Embedded .res File Contains DELU Records          3 Report
PLAT178_ALERT_4_G The CIF-Embedded .res File Contains SIMU Records          3 Report
PLAT186_ALERT_4_G The CIF-Embedded .res File Contains ISOR Records          1 Report
PLAT232_ALERT_2_G Hirshfeld Test Diff (M-X)  Co1      --O12S     .        5.5 s.u.
```

Author Response: In this case there could not be any wrong atom assignment as the nature of the atom is 100 % clear from the ligand structure and composition. In fact, the failury of the test most likel arises from the relative weakness of the corresponding Co-O bond (2.296(3) angstr.) and thus, a relative freedom for the movement of this O atom.

```
PLAT232_ALERT_2_G Hirshfeld Test Diff (M-X)  Co4      --O32S_a   .        6.5 s.u.
```

Author Response: In this case there could not be any wrong atom assignment as the nature of the atom is 100 % clear from the ligand structure and composition. In fact, the failury of the test most likel arises from the relative weakness of the corresponding Co-O bond (2.296(3) angstr.) and thus, a relative freedom for the movement of this O atom.

```
PLAT720_ALERT_4_G Number of Unusual/Non-Standard Labels ..........         81 Note
PLAT794_ALERT_5_G Tentative Bond Valency for Co1       (II)      .       2.02 Info
PLAT794_ALERT_5_G Tentative Bond Valency for Co2       (II)      .       1.84 Info
PLAT794_ALERT_5_G Tentative Bond Valency for Co3       (II)      .       1.85 Info
PLAT794_ALERT_5_G Tentative Bond Valency for Co4       (II)      .       1.98 Info
PLAT794_ALERT_5_G Tentative Bond Valency for Co5       (II)      .       1.82 Info
PLAT860_ALERT_3_G Number of Least-Squares Restraints .............         33 Note
PLAT883_ALERT_1_G No Info/Value for _atom_sites_solution_primary .     Please Do !
PLAT910_ALERT_3_G Missing # of FCF Reflection(s) Below Theta(Min).          3 Note
PLAT912_ALERT_4_G Missing # of FCF Reflections Above STh/L=  0.600         14 Note
PLAT978_ALERT_2_G Number C-C Bonds with Positive Residual Density.          1 Info
```

```
   0 ALERT level A = Most likely a serious problem - resolve or explain
   3 ALERT level B = A potentially serious problem, consider carefully
  15 ALERT level C = Check. Ensure it is not caused by an omission or oversight
  18 ALERT level G = General information/check it is not something unexpected

   1 ALERT type 1 CIF construction/syntax error, inconsistent or missing data
  16 ALERT type 2 Indicator that the structure model may be wrong or deficient
   8 ALERT type 3 Indicator that the structure quality may be low
   6 ALERT type 4 Improvement, methodology, query or suggestion
   5 ALERT type 5 Informative message, check
```

It is advisable to attempt to resolve as many as possible of the alerts in all categories. Often the minor alerts point to easily fixed oversights, errors and omissions in your CIF or refinement strategy, so attention to these fine details can be worthwhile. In order to resolve some of the more serious problems it may be necessary to carry out additional measurements or structure refinements. However, the purpose of your study may justify the reported deviations and the more serious of these should normally be commented upon in the discussion or experimental section of a paper or in the "special_details" fields of the CIF. checkCIF was carefully designed to identify outliers and unusual parameters, but every test has its limitations and alerts that are not important in a particular case may appear. Conversely, the absence of alerts does not guarantee there are no aspects of the results needing attention. It is up to the individual to critically assess their own results and, if necessary, seek expert advice.

Publication of your CIF in IUCr journals

A basic structural check has been run on your CIF. These basic checks will be run on all CIFs submitted for publication in IUCr journals (*Acta Crystallographica*, *Journal of Applied Crystallography*, *Journal of Synchrotron Radiation*); however, if you intend to submit to *Acta Crystallographica Section C* or *E* or *IUCrData*, you should make sure that full publication checks are run on the final version of your CIF prior to submission.

Publication of your CIF in other journals

Please refer to the *Notes for Authors* of the relevant journal for any special instructions relating to CIF submission.

PLATON version of 03/05/2019; check.def file version of 29/04/2019

Datablock Co10 - ellipsoid plot

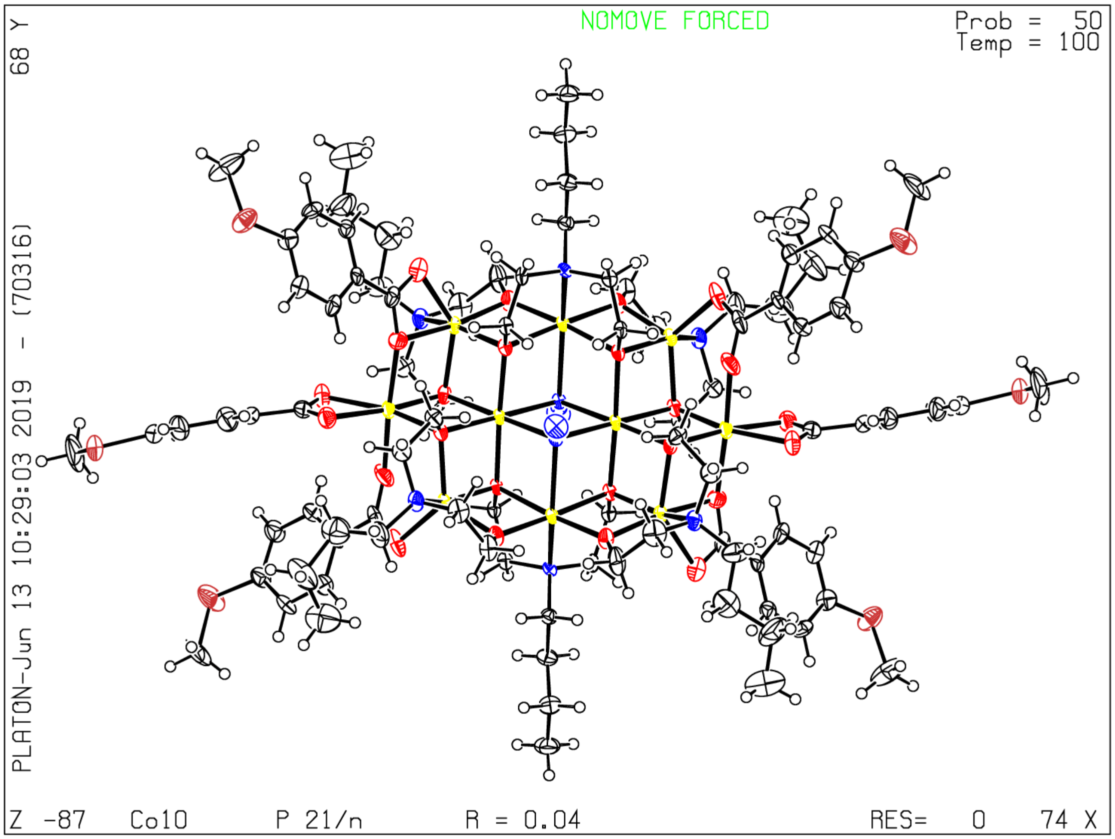

Supplement: Supplementary file 2 [file checkcif_Co10.pdf › checkcif_Co10.pdf]
