# Supplementary material for: Conductive Self-Assembled Monolayers of Paramagnetic {CoIICo4III} and {Co4IICo2III} Coordination Clusters on Gold Surfaces
Source: Front Chem. 2019 Nov 5;7:681. doi: 10.3389/fchem.2019.00681 (PMC6848059; doi:10.3389/fchem.2019.00681)

checkCIF/PLATON report

Structure factors have been supplied for datablock(s) S1769

THIS REPORT IS FOR GUIDANCE ONLY. IF USED AS PART OF A REVIEW PROCEDURE FOR PUBLICATION, IT SHOULD NOT REPLACE THE EXPERTISE OF AN EXPERIENCED CRYSTALLOGRAPHIC REFEREE.

No syntax errors found. CIF dictionary Interpreting this report

Datablock: S1769

```
Bond precision:  C-C = 0.0079 A          Wavelength=0.71073

Cell:           a=20.2361(2)    b=22.46255(17)     c=14.04427(13)
                alpha=90        beta=104.889(1)    gamma=90
Temperature:    120 K

                Calculated                 Reported
Volume          6169.55(10)                6169.55(10)
Space group     P 21/c                     P 21/c
Hall group      -P 2ybc                    -P 2ybc
Moiety formula  C48 H76 Co5 N16 O12 S2, O  ?
Sum formula     C48 H76 Co5 N16 O13 S2     C48 H84 Co5 N16 O13 S2
Mr              1444.02                    1452.08
Dx,g cm-3       1.555                      1.563
Z               4                          4
Mu (mm-1)       1.456                      1.456
F000            2988.0                     3020.0
F000'           2996.87
h,k,lmax        28,31,19                   28,31,19
Nref            17600                      16943
Tmin,Tmax       0.658,0.812                0.503,0.723
Tmin'           0.634

Correction method= # Reported T Limits: Tmin=0.503 Tmax=0.723
AbsCorr = ANALYTICAL

Data completeness= 0.963          Theta(max)= 29.756

R(reflections)= 0.0660( 11919)    wR2(reflections)= 0.1888( 16943)

S = 1.026                   Npar= 765
```

The following ALERTS were generated. Each ALERT has the format
test-name_ALERT_alert-type_alert-level.
Click on the hyperlinks for more details of the test.

Alert level B

PLAT220_ALERT_2_B Non-Solvent Resd 1 C Ueq(max)/Ueq(min) Range 7.6 Ratio

Author Response: The relatively large difference in the Ueeq values of C atoms in cluster could be due to significantly different motion freedom for these atoms. For example, C atoms of phenyl rings are rather tightly fixed in comparison with the methyl groups attached to the S atoms in 4-(methylthio)-benzoate ligands showing correspondingly much higher anisotropic displacement parameters. In any case there are no any alternative for assignments of the electron density maxima in these positions taking into consideration the common sense and the composition of the precursors used for the synthesis of the cluster.

PLAT315_ALERT_2_B Singly Bonded Carbon Detected (H-atoms Missing). C8N5 Check

Author Response: Hydrogen atoms of disordered -CH3 groups were not located.

PLAT315_ALERT_2_B Singly Bonded Carbon Detected (H-atoms Missing). C9N5 Check

Author Response: Hydrogen atoms of disordered -CH3 groups were not located.

PLAT910_ALERT_3_B Missing # of FCF Reflection(s) Below Theta(Min). 15 Note

Author Response: Missing reflections are in diffractometer restricted areas like beamstop. Nevertheless, the total amount of collected reflections well exceeds that required for statistically relevant refinement to assure true results.

Alert level C

PLAT041_ALERT_1_C Calc. and Reported SumFormula Strings Differ Please Check
PLAT043_ALERT_1_C Calculated and Reported Mol. Weight Differ by .. 8.06 Check
PLAT068_ALERT_1_C Reported F000 Differs from Calcd (or Missing)... Please Check
PLAT220_ALERT_2_C Non-Solvent Resd 1 N Ueq(max)/Ueq(min) Range 3.8 Ratio

Author Response: The relatively large difference in the Ueeq values of C atoms in cluster could be due to significantly different motion freedom for these atoms. For example, C atoms of phenyl rings are rather tightly fixed in comparison with the methyl groups attached to the S atoms in 4-(methylthio)-benzoate ligands showing correspondingly much higher anisotropic displacement parameters. In any case there are no any alternative for assignments of the electron density maxima in these positions taking into consideration the common sense and the composition of the precursors used for the synthesis of the cluster.

```
PLAT222_ALERT_3_C Non-Solv.  Resd 1  H   Uiso(max)/Uiso(min) Range        8.6 Ratio
PLAT230_ALERT_2_C Hirshfeld Test Diff for    C2B1     --C7B1     .        5.5 s.u.
PLAT230_ALERT_2_C Hirshfeld Test Diff for    C7N1     --C8N1     .        5.2 s.u.
PLAT230_ALERT_2_C Hirshfeld Test Diff for    C7N2     --C8N2     .        7.0 s.u.
PLAT230_ALERT_2_C Hirshfeld Test Diff for    C7N4     --C8N4     .        5.2 s.u.
PLAT230_ALERT_2_C Hirshfeld Test Diff for    C6N5     --C7N5     .        6.7 s.u.
PLAT242_ALERT_2_C Low    'MainMol' Ueq as Compared to Neighbors of         S1 Check
PLAT341_ALERT_3_C Low Bond Precision on  C-C Bonds ...............    0.00788 Ang.
PLAT905_ALERT_3_C Negative K value in the Analysis of Variance ...     -1.138 Report
PLAT911_ALERT_3_C Missing FCF Refl Between Thmin & STh/L=    0.600          7 Report
PLAT971_ALERT_2_C Check Calcd Resid. Dens.  1.37A   From S2              1.57 eA-3
PLAT972_ALERT_2_C Check Calcd Resid. Dens.  0.67A   From S2             -1.59 eA-3
PLAT973_ALERT_2_C Check Calcd Positive Resid. Density on       Co5       1.23 eA-3
PLAT973_ALERT_2_C Check Calcd Positive Resid. Density on       Co4       1.15 eA-3
PLAT973_ALERT_2_C Check Calcd Positive Resid. Density on       Co1       1.15 eA-3
PLAT973_ALERT_2_C Check Calcd Positive Resid. Density on       Co2       1.10 eA-3
PLAT973_ALERT_2_C Check Calcd Positive Resid. Density on       Co3       1.07 eA-3
PLAT977_ALERT_2_C Check Negative Difference Density on H1SC             -0.31 eA-3
PLAT977_ALERT_2_C Check Negative Difference Density on H5NC             -0.61 eA-3
PLAT978_ALERT_2_C Number C-C Bonds with Positive Residual Density.          0 Info
```

Alert level G

```
FORMU01_ALERT_2_G  There is a discrepancy between the atom counts in the
            _chemical_formula_sum and the formula from the _atom_site* data.
            Atom count from _chemical_formula_sum:C48 H84 Co5 N16 O13 S2
            Atom count from the _atom_site data:  C48 H76 Co5 N16 O13 S2
CELLZ01_ALERT_1_G Difference between formula and atom_site contents detected.
CELLZ01_ALERT_1_G WARNING: H atoms missing from atom site list. Is this intentional?
           From the CIF: _cell_formula_units_Z    4
           From the CIF: _chemical_formula_sum  C48 H84 Co5 N16 O13 S2
           TEST: Compare cell contents of formula and atom_site data

           atom    Z*formula  cif sites diff
           C        192.00    192.00    0.00
           H        336.00    304.00   32.00
           Co        20.00     20.00    0.00
           N         64.00     64.00    0.00
           O         52.00     52.00    0.00
           S          8.00      8.00    0.00
PLAT003_ALERT_2_G Number of Uiso or Uij Restrained non-H Atoms ...          8 Report
PLAT083_ALERT_2_G SHELXL Second Parameter in WGHT  Unusually Large      22.35 Why ?
PLAT142_ALERT_4_G s.u. on b - Axis Small or Missing ..............    0.00017 Ang.
PLAT143_ALERT_4_G s.u. on c - Axis Small or Missing ..............    0.00013 Ang.
PLAT171_ALERT_4_G The CIF-Embedded .res File Contains EADP Records          2 Report
PLAT177_ALERT_4_G The CIF-Embedded .res File Contains DELU Records          1 Report
PLAT178_ALERT_4_G The CIF-Embedded .res File Contains SIMU Records          1 Report
PLAT186_ALERT_4_G The CIF-Embedded .res File Contains ISOR Records          1 Report
PLAT301_ALERT_3_G Main Residue  Disorder ..............(Resd  1  )         1% Note
PLAT302_ALERT_4_G Anion/Solvent/Minor-Residue Disorder (Resd  2  )       100% Note
PLAT302_ALERT_4_G Anion/Solvent/Minor-Residue Disorder (Resd  3  )       100% Note
PLAT302_ALERT_4_G Anion/Solvent/Minor-Residue Disorder (Resd  4  )       100% Note
PLAT304_ALERT_4_G Non-Integer Number of Atoms in ...... Resd  2          0.39 Check
PLAT304_ALERT_4_G Non-Integer Number of Atoms in ...... Resd  3          0.32 Check
PLAT304_ALERT_4_G Non-Integer Number of Atoms in ...... Resd  4          0.29 Check
PLAT311_ALERT_2_G Isolated Disordered Oxygen Atom (No H's ?) .....       O1W1 Check
PLAT311_ALERT_2_G Isolated Disordered Oxygen Atom (No H's ?) .....       O2W1 Check
PLAT311_ALERT_2_G Isolated Disordered Oxygen Atom (No H's ?) .....       O3W1 Check
PLAT367_ALERT_2_G Long?  C(sp?)-C(sp?) Bond  C6N5     - C7N5     .       1.52 Ang.
PLAT432_ALERT_2_G Short Inter X...Y Contact  S1       ..C9N5             2.57 Ang.
                                                   -1+x,y,z  =      1_455 Check
```

```
PLAT720_ALERT_4_G Number of Unusual/Non-Standard Labels ..........        138 Note
PLAT793_ALERT_4_G Model has Chirality at N1         (Centro SPGR)          R Verify
PLAT793_ALERT_4_G Model has Chirality at N2         (Centro SPGR)          R Verify
PLAT793_ALERT_4_G Model has Chirality at N4         (Centro SPGR)          R Verify
PLAT793_ALERT_4_G Model has Chirality at N5         (Centro SPGR)          R Verify
PLAT794_ALERT_5_G Tentative Bond Valency for Co1      (III)     .       3.10 Info
PLAT794_ALERT_5_G Tentative Bond Valency for Co2      (III)     .       3.08 Info
PLAT794_ALERT_5_G Tentative Bond Valency for Co3      (II)      .       1.80 Info
PLAT794_ALERT_5_G Tentative Bond Valency for Co4      (III)     .       3.12 Info
PLAT794_ALERT_5_G Tentative Bond Valency for Co5      (III)     .       3.10 Info
PLAT860_ALERT_3_G Number of Least-Squares Restraints .............         56 Note
PLAT883_ALERT_1_G No Info/Value for _atom_sites_solution_primary .     Please Do !
PLAT912_ALERT_4_G Missing # of FCF Reflections Above STh/L=  0.600        634 Note
PLAT913_ALERT_3_G Missing # of Very Strong Reflections in FCF ....          2 Note
PLAT933_ALERT_2_G Number of OMIT Records in Embedded .res File ...         12 Note
```

```
  0 ALERT level A = Most likely a serious problem - resolve or explain
  4 ALERT level B = A potentially serious problem, consider carefully
 24 ALERT level C = Check. Ensure it is not caused by an omission or oversight
 38 ALERT level G = General information/check it is not something unexpected

  6 ALERT type 1 CIF construction/syntax error, inconsistent or missing data
 29 ALERT type 2 Indicator that the structure model may be wrong or deficient
  8 ALERT type 3 Indicator that the structure quality may be low
 18 ALERT type 4 Improvement, methodology, query or suggestion
  5 ALERT type 5 Informative message, check
```

It is advisable to attempt to resolve as many as possible of the alerts in all categories. Often the minor alerts point to easily fixed oversights, errors and omissions in your CIF or refinement strategy, so attention to these fine details can be worthwhile. In order to resolve some of the more serious problems it may be necessary to carry out additional measurements or structure refinements. However, the purpose of your study may justify the reported deviations and the more serious of these should normally be commented upon in the discussion or experimental section of a paper or in the "special_details" fields of the CIF. checkCIF was carefully designed to identify outliers and unusual parameters, but every test has its limitations and alerts that are not important in a particular case may appear. Conversely, the absence of alerts does not guarantee there are no aspects of the results needing attention. It is up to the individual to critically assess their own results and, if necessary, seek expert advice.

Publication of your CIF in IUCr journals

A basic structural check has been run on your CIF. These basic checks will be run on all CIFs submitted for publication in IUCr journals (*Acta Crystallographica*, *Journal of Applied Crystallography*, *Journal of Synchrotron Radiation*); however, if you intend to submit to *Acta Crystallographica Section C* or *E* or *IUCrData*, you should make sure that full publication checks are run on the final version of your CIF prior to submission.

Publication of your CIF in other journals

Please refer to the *Notes for Authors* of the relevant journal for any special instructions relating to CIF submission.

PLATON version of 03/05/2019; check.def file version of 29/04/2019

Datablock S1769 - ellipsoid plot

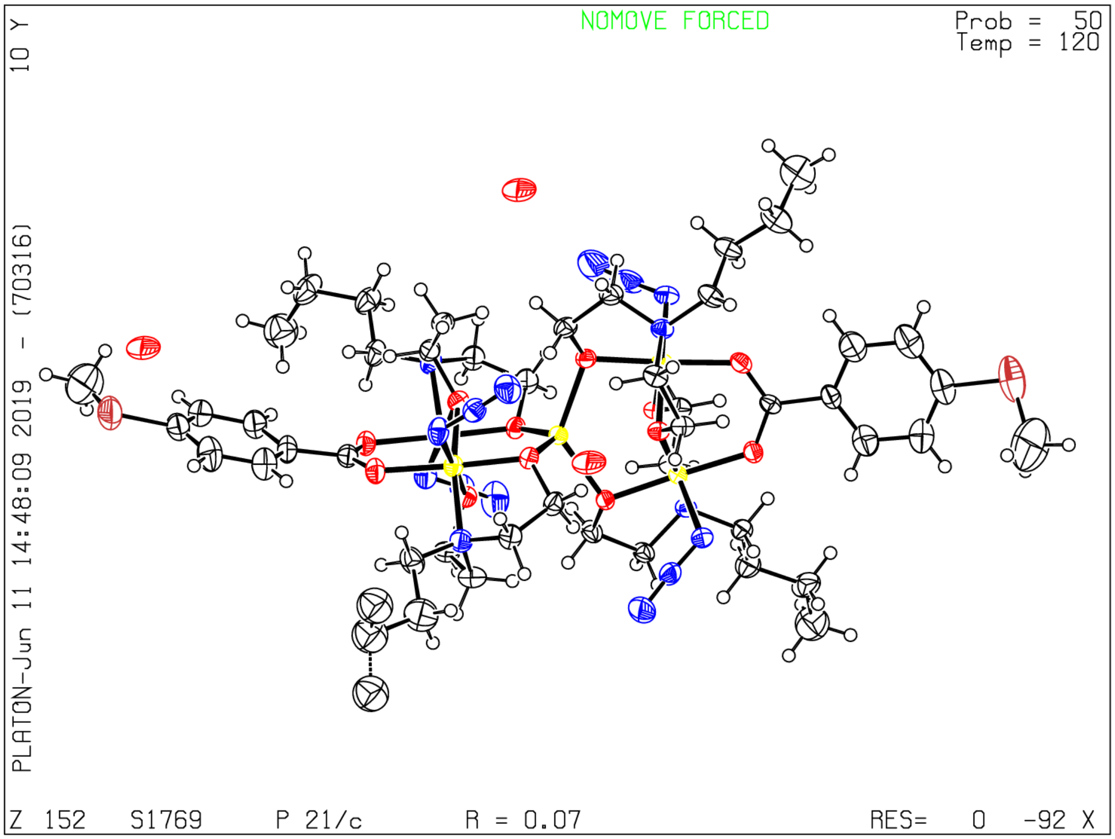

Supplement: Supplementary file 2 [file checkcif_Co5.pdf › checkcif_Co5.pdf]
